# Embryonic abnormalities and genotoxicity induced by 2,4-dichlorophenoxyacetic acid during indirect somatic embryogenesis in *Coffea*

**DOI:** 10.1038/s41598-023-36879-7

**Published:** 2023-06-15

**Authors:** João Paulo de Morais Oliveira, Alex Junior da Silva, Mariana Neves Catrinck, Wellington Ronildo Clarindo

**Affiliations:** 1 Laboratório de Citogenética e Cultura de Tecidos Vegetais, Centro de Ciências Agrárias e Engenharias, Universidade Federal do Espírito Santo, Alegre, ES 29.500-000 Brazil; 2 Laboratório de Citogenética e Citometria, Departamento de Biologia Geral, Universidade Federal de Viçosa, Viçosa, MG 36.570-900 Brazil

**Keywords:** DNA methylation, Plant morphogenesis, Auxin

## Abstract

Indirect somatic embryogenesis (ISE) is a morphogenetic pathway in which somatic cells form callus and, later, somatic embryos (SE). 2,4-dichlorophenoxyacetic acid (2,4-D) is a synthetic auxin that promotes the proliferation and dedifferentiation of somatic cells, inducing the ISE. However, 2,4-D can cause genetic, epigenetic, physiological and morphological disorders, preventing the regeneration and/or resulting abnormal somatic embryos (ASE). We aimed to evaluate the toxic 2,4-D effect during the *Coffea arabica* and *C. canephora* ISE, assessing the SE morphology, global 5-methylcytosine levels (5-mC%) and DNA damage. Leaf explants were inoculated in media with different 2,4-D concentrations. After 90 days, the friable calli were transferred to the regeneration medium, and the number of normal and abnormal SE was monthly counted. The increase of the 2,4-D concentration increased the number of responsive explants in both *Coffea*. At 9.06, 18.08 and 36.24 μM 2,4-D, *C. arabica* presented the highest values of responsive explants, differing from *C. canephora*. Normal and abnormal SE regeneration increased in relation to the time and 2,4-D concentration. Global 5-mC% varied at different stages of the ISE in both *Coffea*. Furthermore, the 2,4-D concentration positively correlated with global 5-mC%, and with the mean number of ASE. All ASE of *C. arabica* and *C. canephora* exhibited DNA damage and showed higher global 5-mC%. The allotetraploid *C. arabica* exhibited greater tolerance to the toxic effect of 2,4-D than the diploid *C. canephora*. We conclude that synthetic 2,4-D auxin promotes genotoxic and phytotoxic disorders and promotes epigenetic changes during *Coffea* ISE.

## Introduction

Indirect somatic embryogenesis (ISE) is a morphogenetic pathway that involves the callus formation and, later, somatic embryos (SE) regeneration. ISE is established in a sterile and appropriate in vitro condition, from the inoculation of tissue from explant donors^1^. The first in vitro approach involving *Coffea* was carried out by Staritsky^2^, which established somatic embryogenesis. The ability of a given tissue to generate SE is a characteristic restricted to a limited fraction of the cells^3^. For differentiation, cells need to acquire competence and follow a new morphogenetic pathway^1^. However, a new hypothesis has been proposed by Campos et al.^4^, in which meristematic cells are able to differentiate into SE without cell dedifferentiation. This debate is based on in vitro approaches that have elucidated the biological issues involved in the dedifferentiation, proliferation, and regeneration of callus, SE and plantlets. These issues provide the detailed analysis of the “omics” at different levels, as well as their interaction^5–11^.

2,4-dichlorophenoxyacetic (2,4-D) growth regulator is widely used in direct embryogenesis and in ISE, playing a crucial role in the cell dedifferentiation and SE recovering^12^. This growth regulator is related to the cell division at the S input and the G_2_-M transition^13^, and also to the cell expansion processes^14^. So, 2,4-D is the most used in *Coffea* ISE, which requires the initial presence of this auxin in the pre-incubation period or induction stages^8–10,15,16^. However, the high explant exposure to 2,4-D results in the formation of abnormal somatic embryos (ASE), due to the interruption of the genetic and physiological processes of the cells. This disruption causes a rapid efflux of protons, enzymatic activation, protein transcription and translation, and polysaccharide synthesis, resulting in loss of cell wall stability, depletion of reserve substances and inactivation of cell repair mechanisms^12,17^.

2,4-D can also cause genetic and epigenetic changes^17^. This is undesirable in *Coffea* propagation due to loss of genetic fidelity and ASE formation, compromising superior genotypes^18–20^. Phenotypic variants of *C. arabica* trees, which were regenerated in vitro, were induced by aneuploidy^19^. *C. arabica* plants regenerated in vitro showed genetic differences in relation to plant explant donors, since the somaclonal variation was revealed by differential fragments amplified by sequence-related amplified polymorphism (SRAP) primers^20^. Landey et al.^18^ reported that is low the frequency of genetic and epigenetic changes during somatic embryogenesis in *C. arabica*. According to these authors, the aneuploidy is main genome modification in most phenotypic variants, indicating that missegregation during the anaphase plays a major role in somaclonal variation in *C. arabica*.

Cell reprogramming is complex and often concomitant with significant changes in chromatin status. Chromatin change is characterized by DNA methylation and histone chemical modifications (mainly methylation or acetylation)^21^. In *Coffea*, epigenetic changes occur during somatic embryogenesis characterized by cytosine methylation^6,8–10^. These epigenetic changes occur due to the in vitro environment, promoting an important role to regulate and control genes involved with the morphogenetic pathway^6^. Epigenetic variations can be transient and altered through DNA methylation, as well as acetylation, phosphorylation, methylation and ubiquitination of the histones^1^. However, cell proliferation and SE and plantlets recovering are influenced by coordination of genetic and epigenetic factors^22^. When these factors are not coordinated, the main consequence is non-establishment of the ISE or the large-scale production of ASE^17^. The main abnormalities exhibited in SE are fusion of two or more embryos, absence of apical and root meristems, translucent embryos, multiple cotyledons and loss of bipolarity^10,17^. Generally, embryonic anomalies are associated with point mutations^19,20^, aneuploidy^18^ and/or epigenetic changes^10^. 2,4-D is one of the precursors for the emergence of these abnormalities because this herbicide can be toxic even in minimal concentrations^17^. Therefore, it is essential to quantify global methylation levels (5-mC%) during ISE and to evaluate the toxic effect of 2,4-D on SE regeneration in *Coffea*.

Comet Assay evidences DNA single-strand and/or double-strand breaks in individual cells^23^. For this reason, Comet Assay has been applied to assess the genotoxic effect of chemical, physical and biological agents. For example, Özkul et al.^24^ verified the 2,4-D genotoxic effect on *Allium cepa* L. roots from seeds germinated in semisolid medium supplement with ~ 18.08 μM 2,4-D for 48 h. However, the Comet Assay has not applied to assess DNA integrity in SE regenerated via ISE, in which 2,4-D is used to induce friable callus proliferation. From *C. arabica* and *C. canephora*, we aimed to: (a) induce ISE from different 2,4-D concentrations, (b) quantify the 5-mC% during ISE, (c) verify the effect of the 2,4-D and 5-mC% during ISE, and (d) assess the genotoxicity and phytotoxicity of the remaining 2,4-D on regenerated SE.

## Results

### Callogenesis

**Figure 1:**
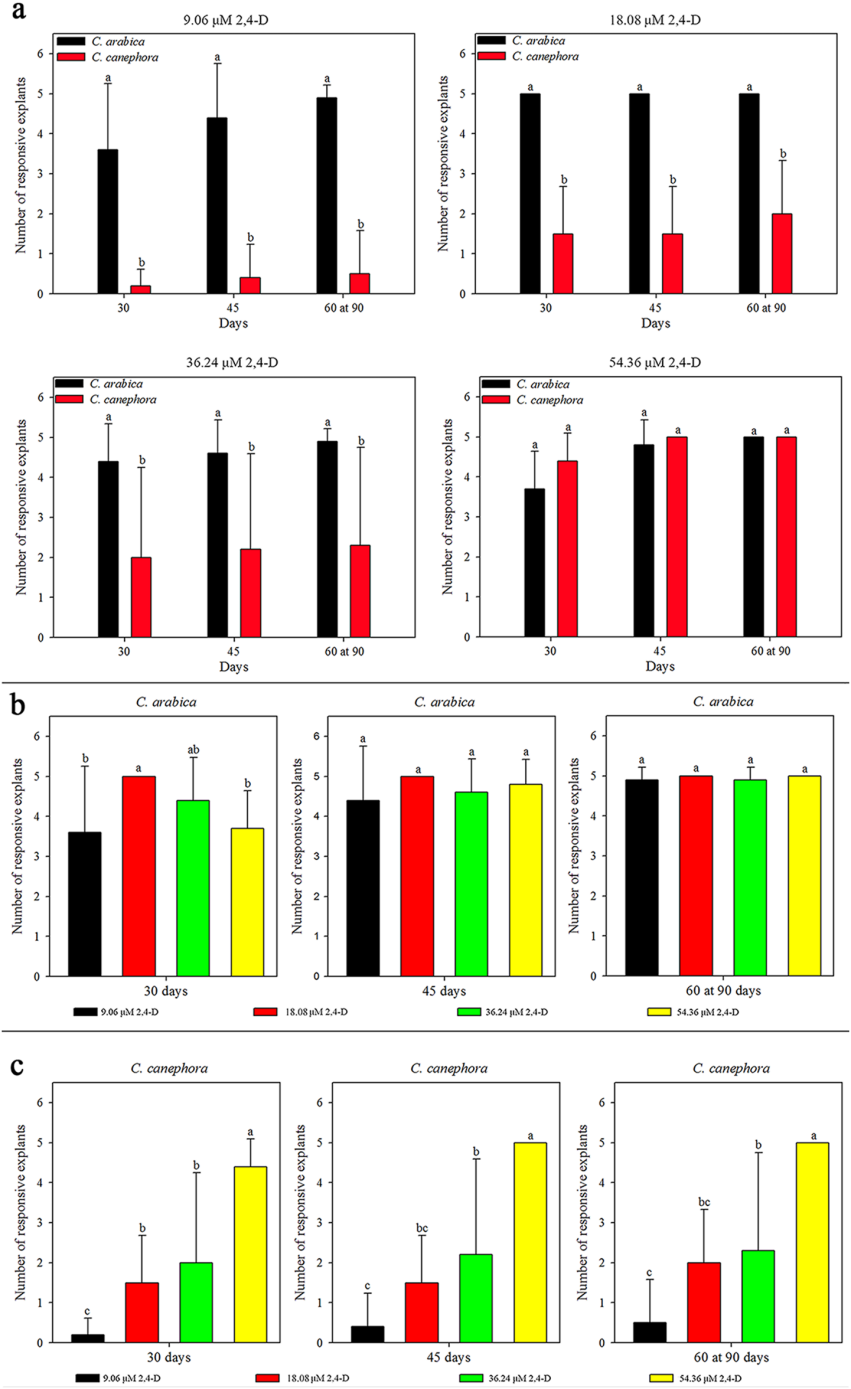
Friable callus induction (mean values and standard deviation) from *C*. *arabica* and *C*. *canephora* leaf explants in different 2,4-D concentrations over 90 days. *C. arabica* and *C. canephora* exhibited distinct values of responsive explants in 9.06–54.36 μM 2,4-D over 90 days (**a**). 2,4-D concentrations influenced the callus inductions over time in *C. arabica* (**b**) and in *C. canephora* (**c**). *Mean values followed by the same letter do not present a significant difference.

*C. arabica* and *C. canephora* exhibited a distinct mean number of responsive explants (leaf fragment with callus) influenced by 2.4-D (Fig. 1, SI Fig. 1, SI Table 1). The first responsive explants of *C. arabica* and *C. canephora* were observed at 30 days for all media. *C. arabica* exhibited the higher mean number of responsive explants than *C. canephora* in 9.06 – 36.24 μM 2,4-D at all times. In 54.36 μM 2,4-D, *C. arabica* and *C. canephora* showed the same mean number of responsive explants (Fig. 1a).

Considering the 2,4-D concentration, 18.08 µM after 30 days provided the highest mean number of *C. arabica* responsive explants than 9.06 and 54.36 μM. The concentration of 36.24 μM 2,4-D resulted the same mean number of responsive explants than the other concentrations. After 30 days, the mean number of *C. arabica* responsive explants was not influenced by 2,4-D (Fig. 1b). For *C. canephora*, 54.36 μM 2,4-D had the highest mean number of responsive explants at all times. 18.08 and 36.24 μM 2,4-D were statistically equal at 30 days, as well as these concentrations were superior to the 9.06 μM 2,4-D. After 30 days, 36.24 μM 2,4-D exhibited more responsive explants than 9.06 μM 2,4-D. In the same period, 36.24 μM 2,4-D was equal to 18.08 μM 2,4-D, which showed the same mean number than 9.06 μM (Fig. 1c).

The mean number of responsive explants of *C. arabica* increased over time in 9.06, 36.24 and 54.36 μM 2,4-D (SI Fig. 1a). In *C. canephora*, the mean number of responsive explants increased over time for all 2,4-D concentrations (SI Fig. 1b). All callus showed a pale yellow and friable appearance (SI Fig. 1). Briefly, induction media with different 2,4-D concentrations in *C. arabica* influenced the callus induction only up to 30 days. In *C. canephora*, the best medium to induce responsive explants was with 54.36 μM 2,4-D.

### Somatic embryo regeneration

**Figure 2:**
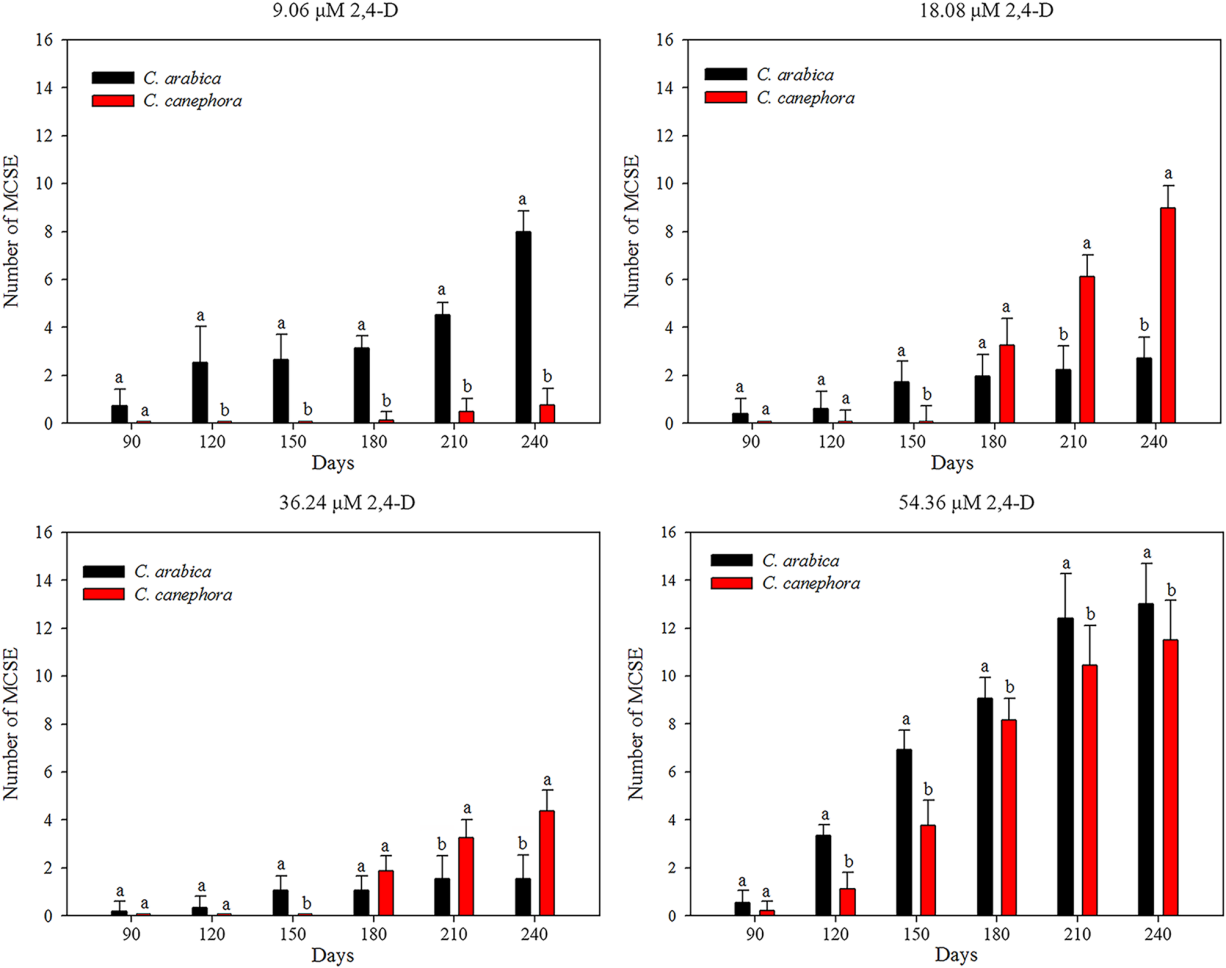
The origin of the friable callus (9.06–54.36 μM 2,4-D) influenced the MCSE regeneration in *C. arabica* and *C. canephora* over 240 days. *C. arabica* and *C. canephora* exhibited distinct MCSE values (mean values and standard deviation), considering 2,4-D concentrations and time. *Mean values followed by the same letter do not present a significant difference.

*C. arabica* and *C. canephora* exhibited different mean numbers of normal mature cotyledon somatic embryo (MCSE). The origin of the friable callus (9.06, 18.08, 36.24 or 54.36 μM 2,4-D) and time (30, 60, 90, 120, 150, 180, 210 and 240 days) influenced the MCSE regeneration (SI Table 2). The first MCSE were observed at 90 days for *C. arabica* friable callus induced in all 2,4-D concentrations, at 90 days for *C. canephora* friable callus in 54.36 μM 2,4-D, and at 150 days for 9.06, 18.08 and 36.24 μM 2,4-D. *C. arabica* friable callus originated in 9.06 and 54.36 μM 2,4-D regenerated more MCSE than *C. canephora* friable callus at all evaluated times. *C. arabica* friable callus originated in 18.08 and 36.24 μM 2,4-D regenerated more MCSE than *C. canephora* at 150 days, and less after 180 days (Fig. 2).

**Figure 3:**
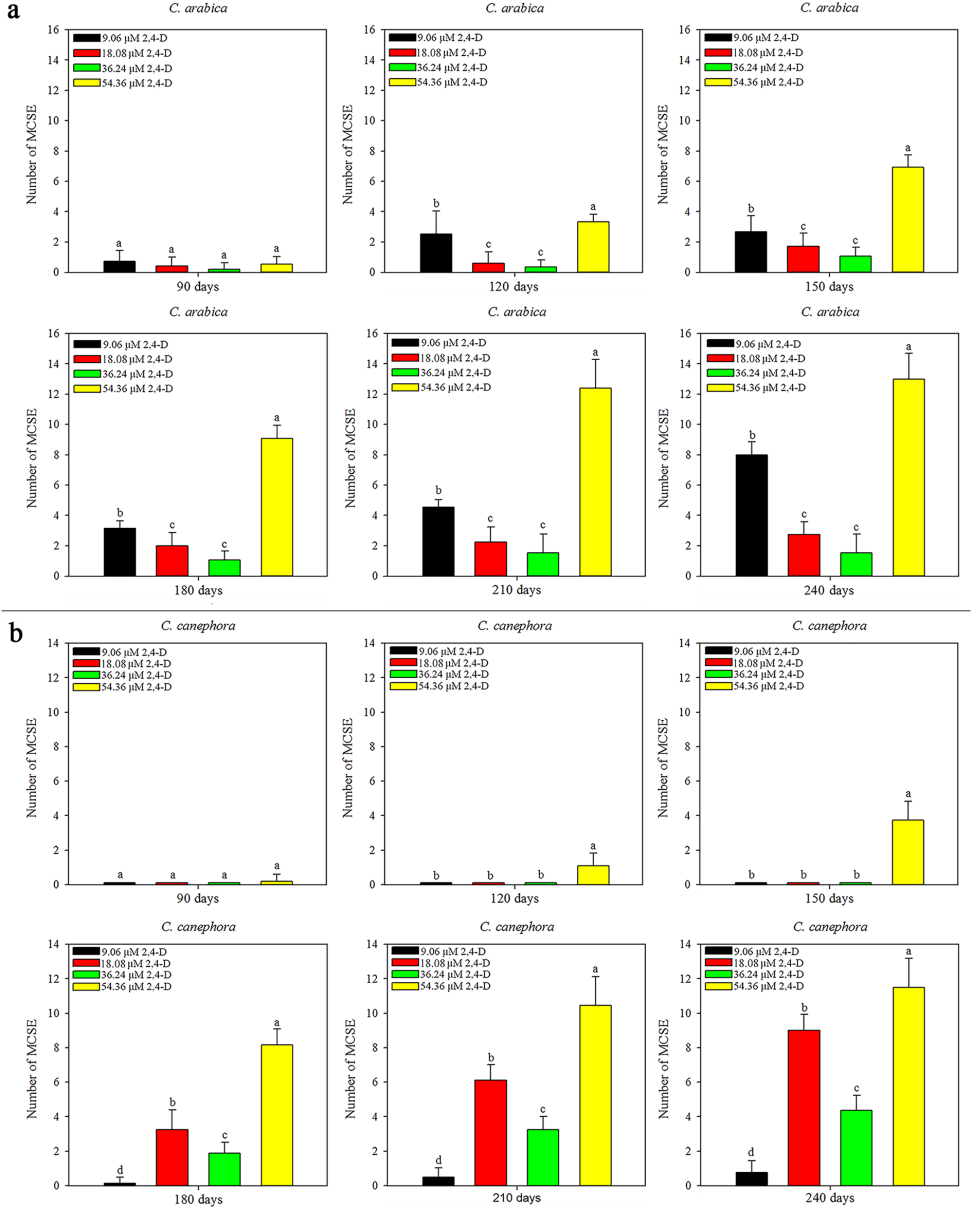
MCSE regeneration from *C. arabica* and *C. canephora* friable callus (mean values and standard deviation). The 2,4-D concentrations, which gave rise to friable calli, influenced MCSE regeneration during the ISE in *C. arabica* (**a**) and in *C. canephora* (**b**). *Mean values followed by the same letter do not present a significant difference.

*C. arabica* friable callus origin influenced the MCSE regeneration, since after 90 days the friable callus from 54.36 μM 2,4-D resulted in the higher mean MCSE number, followed by 9.06 μM 2,4-D and, later, 18.08 and 36.24 μM 2,4-D (Fig. 3a). In *C. canephora*, the origin of friable callus at different concentrations of 2,4-D influenced the regeneration of MCSE after 90 days. The greatest regeneration of MCSE was observed in friable callus induced in 54.36 μM 2,4-D. Differently, the MCSE regeneration was identical at 120 and 150 days for friable callus originating at 9.06, 18.08 and 36.24 μM 2,4-D. After 150 days, the greatest MCSE regeneration was observed in friable callus from 18.08 μM, followed by 36.24 μM and finally 9.06 μM 2,4-D (Fig. 3b). Therefore, MCSE regeneration increased over time, and the best friable calli were induced in 54.36 μM 2,4-D for both species (SI Fig. 2).

**Figure 4:**
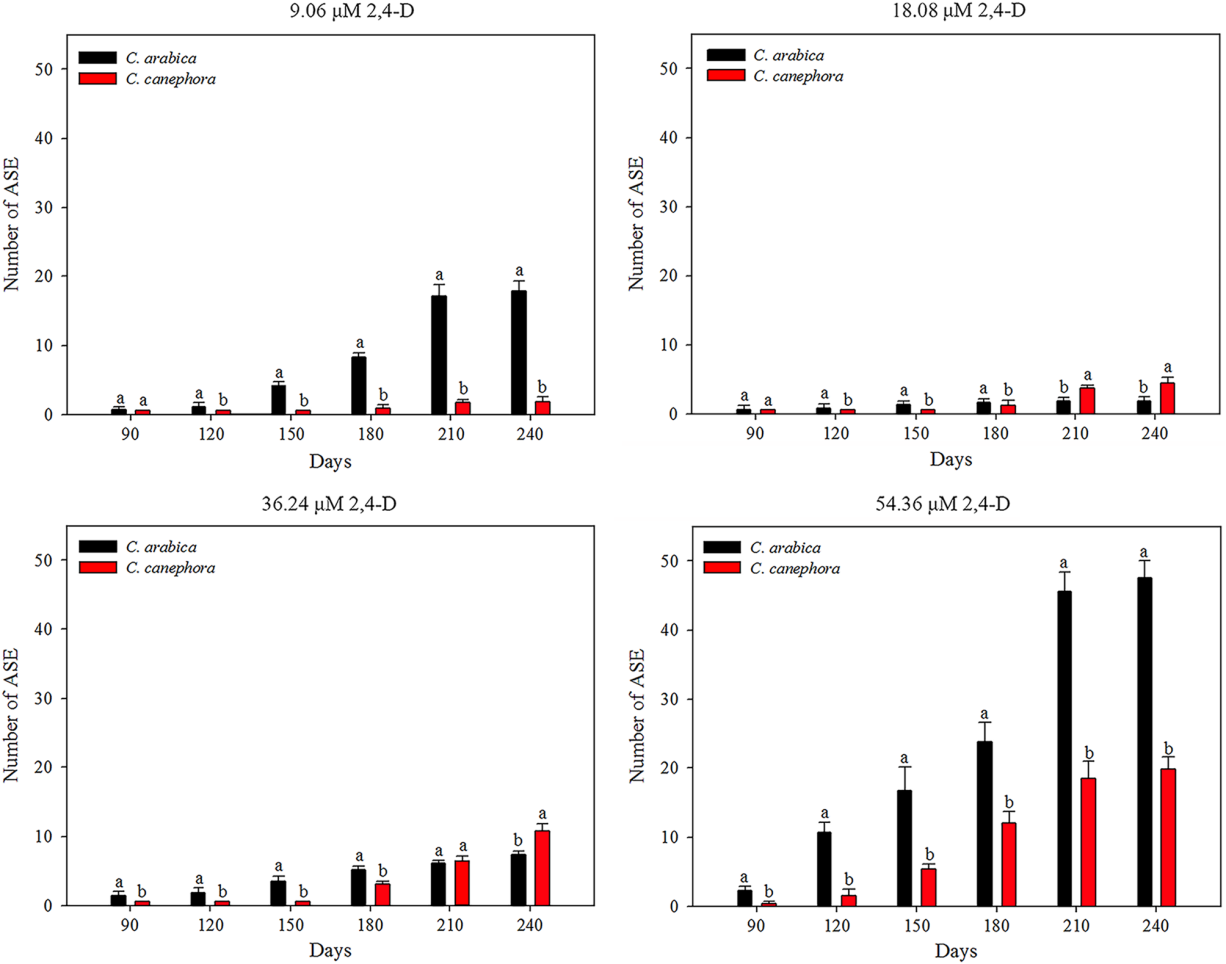
The origin of the friable callus (9.06–54.36 μM 2,4-D) influenced the ASE regeneration of *C. arabica* and *C. canephora* over 240 days. *C. arabica* and *C. canephora* exhibited distinct ASE values (mean values and standard deviation), considering 2,4-D concentrations and time. *Mean values followed by the same letter do not present a significant difference.

*C. arabica* and *C. canephora* exhibited different mean numbers of ASE. The origin of the friable callus (9.06, 18.08, 36.24 or 54.36 μM 2,4-D) and time (30, 60, 90, 120, 150, 180, 210 and 240 days) influenced the ASE regeneration (SI Table 3). The first ASE were observed at 90 days for *C. arabica* friable callus induced in all 2,4-D concentrations and for friable callus of *C. canephora* at 54.36 μM 2,4-D, and at 180 days for 9.06, 18.08 and 36.24 μM 2,4-D. *C. arabica* friable callus from 9.06 to 54.36 μM 2,4-D regenerated more ASE than *C. canephora* friable callus at all times evaluated. Friable callus of *C. arabica* from 18.08 μM 2,4-D regenerated more ASE than of *C. canephora* during 180 days. After 180 days, *C. canephora* friable callus provided more ASE. The *C. arabica* friable callus from 36.24 μM 2,4-D regenerated more ASE than *C. canephora* friable callus during 180 days. At 210 days, ASE regeneration was equal and, at 240 days, ASE regeneration was higher in the friable callus of *C. canephora* (Fig. 4).

**Figure 5:**
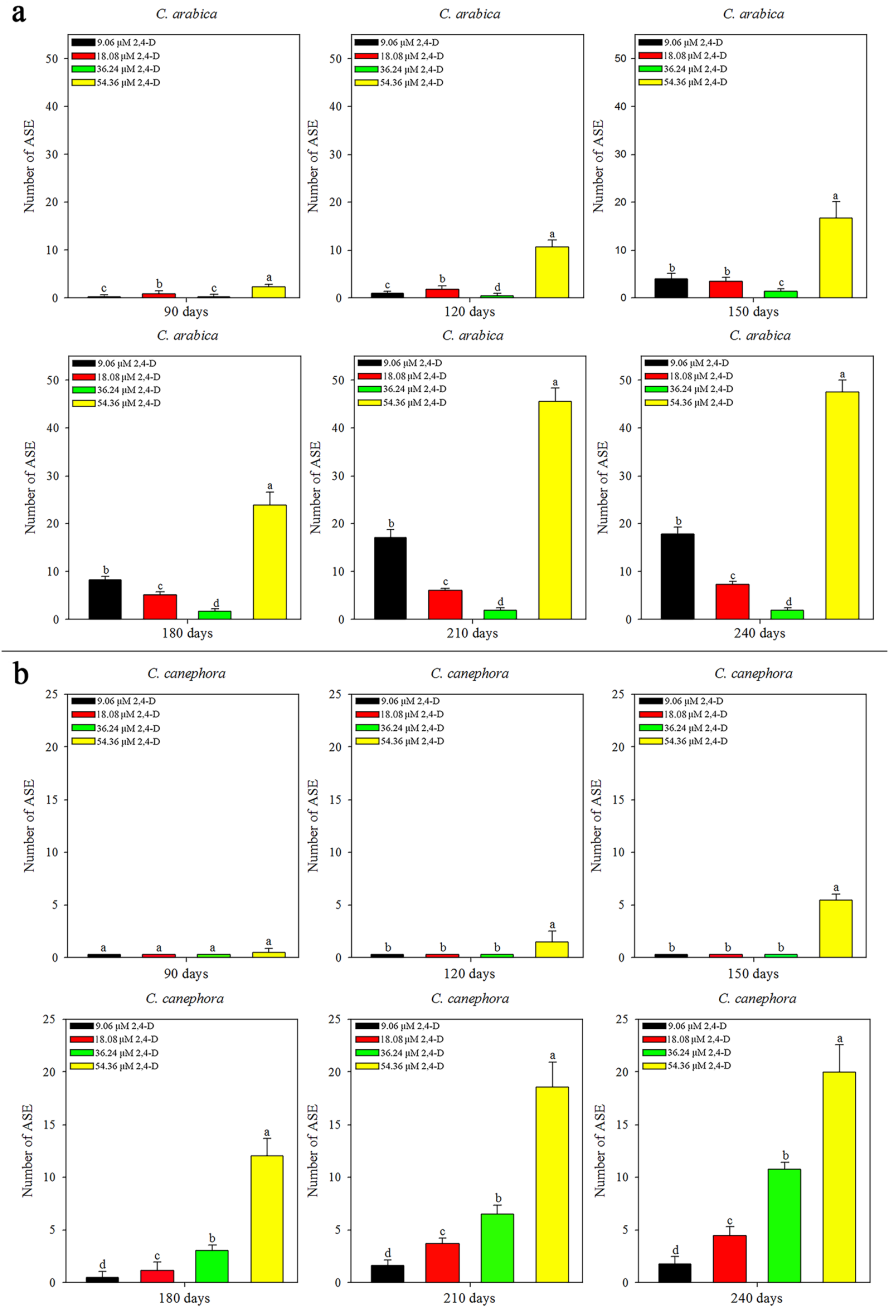
ASE regeneration from *C. arabica* and *C. canephora* friable callus (mean values and standard deviation). The 2,4-D concentrations that gave rise to friable calli influenced ASE regeneration during ISE in *C. arabica* (**a**) and in *C. canephora* (**b**). *Mean values followed by the same letter do not present a significant difference.

In *C. arabica*, the friable callus of 54.36 μM 2,4-D regenerated more ASE at all times evaluated. At 90 and 120 days, the friable callus of 18.08 μM 2,4-D regenerated more ASE than the 9.06 and 36.24 μM 2,4-D. ASE regeneration increased in the friable callus of 9.06 μM 2,4-D, surpassing the friable callus of 36.24 μM 2,4-D at 120 days. At 150 days, ASE regeneration increased in friable callus of 9.06 μM 2,4-D, equaling with friable callus of 18.08 μM 2,4-D. After 150 days, ASE regeneration continued to increase in friable callus of 9.06 μM 2,4-D, overcoming the friable callus of 18.08 μM 2,4-D. Also, the friable callus of 18.08 μM 2,4-D resulted more ASE than friable callus of 36.24 μM 2,4-D in all time evaluated (Fig. 5a). In *C. canephora*, ASE regeneration was equal at 90 days for all friable callus originated in the induction medium with 9.06, 18.08, 36.24 and 54.36 μM 2,4-D. After 90 days, ASE regeneration increased in friable callus of 54.36 μM 2,4-D, overcoming the others and differing over time. ASE regeneration in friable callus of 9.06, 18.08 and 36.24 μM 2,4-D was equal to 120 and 150 days. After 150 days, the friable callus of 36.24 μM 2,4-D regenerated more ASE than the friable callus of 18.08 μM 2,4-D. In the same time, the friable callus of 18.08 μM 2,4-D regenerated more ASE than friable callus of 9.06 µM 2,4-D (Fig. 5b). As well as observed for MCSE, the ASE regeneration increases over time, and more ASE was observed in friable callus from 54.36 μM 2,4-D for both species (SI Fig. 3).

### Global 5-mC%

**Figure 6:**
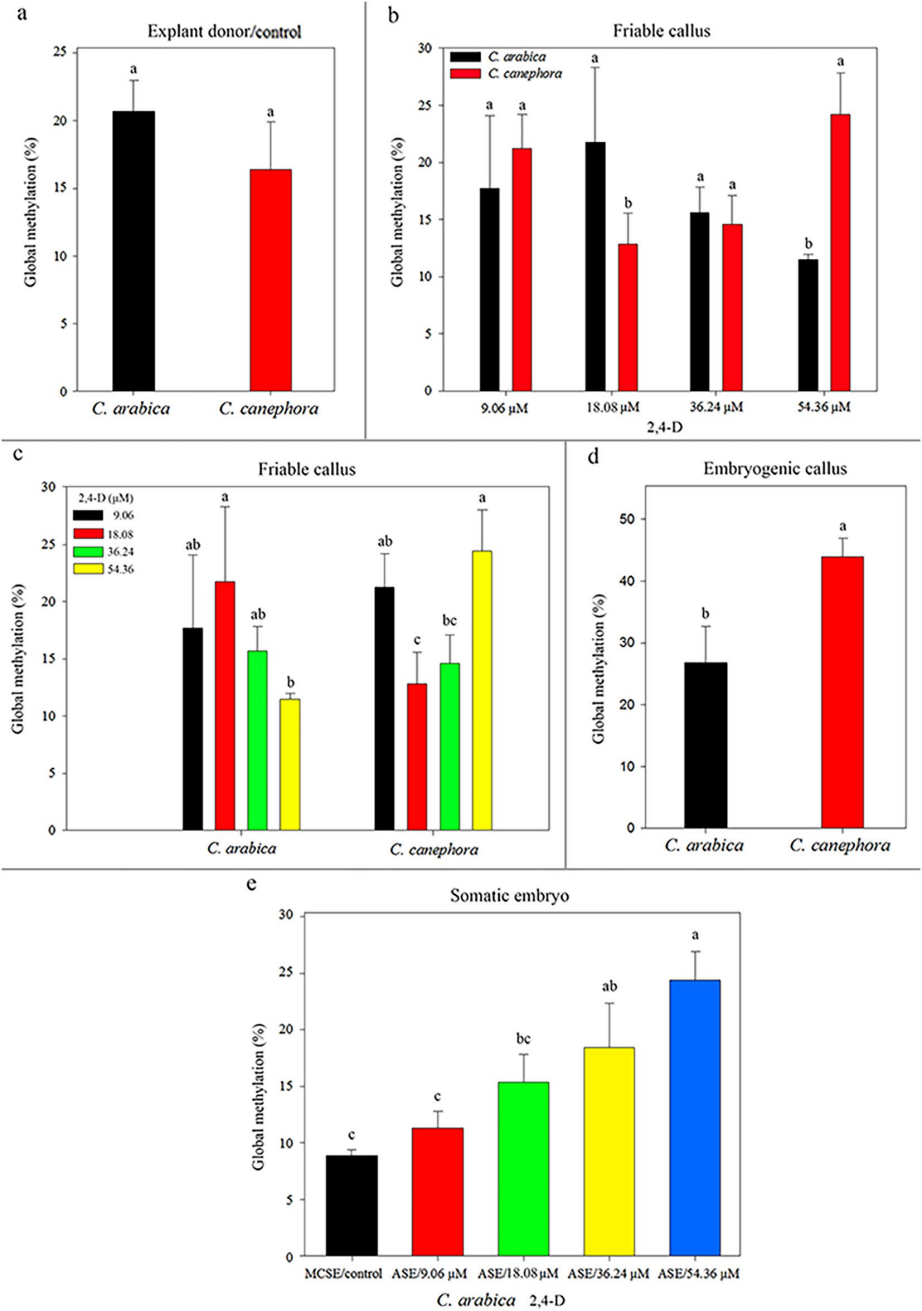
5-mC% during *C. arabica* and *C. canephora* ISE (mean values and standard deviation). Global 5-mC% in *C. arabica* and *C. canephora* leaf explant (control) donors (**a**). Comparison of the 5-mC% between the *C. arabica* and *C. canephora* friable callus originated in the induction medium with 9.06–54.36 μM 2,4-D (**b**). Comparison of the 5-mC% of the friable callus originated from 9.06 to 54.36 μM 2,4-D for *C. arabica* and *C. canephora* (**c**). 5-mC% in embryogenic callus of *C. arabica* and *C. canephora* (**d**). 5-mC% in MCSE (control) and ASE of *C. arabica* regenerated from friable callus originated in 9.06–54.36 μM of 2,4-D (**e**). *Mean values followed by the same letter do not present a significant difference.

Although statistically equal between the *Coffea* explant donors, the 5-mC% varied between *C. arabica* and *C. canephora* during the ISE (friable callus, embryogenic callus, ASE and MCSE) (SI Table 4). *C. canephora* and *C. arabica* explant donors (control) exhibited mean values of 20.60 and 16.37 5-mC% and did not differ statistically (Fig. 6a). Friable callus of *C. arabica* and *C. canephora* induced at different concentrations of 2,4-D exhibited distinct 5-mC%. In the induction media with 9.06 and 36.24 μM 2,4-D, friable callus of *C. arabica* and *C. canephora* did not differ statistically in relation to 5-mC%. However, in the induction media with 18.08 and 54.36 μM 2,4-D, the friable callus of *C. arabica* and *C. canephora* exhibited distinct values of 5-mC% (Fig. 6b). Friable callus of *C. arabica* showed a mean value of 21.76 5-mC% in induction medium with 18.08 μM 2,4-D and differed from friable callus of *C. canephora*, which showed a mean value of 12.84 5-mC%. In the induction medium with 54.36 μM 2,4-D, the opposite was observed. The friable callus of *C. canephora* exhibited a mean value of 24.20 5-mC%, differing from the friable callus of *C. arabica*, which showed a mean value of 11.50 5-mC%. 2,4-D concentrations influenced the 5-mC% in the friable calli of each species. In *C. arabica*, friable callus induced in culture medium with 18.08 μM 2,4-D exhibited a mean value of 21.76 5-mC% and differed from friable callus induced with 54.36 μM 2,4-D, which exhibited a mean value of 11.48 5-mC%. In *C. canephora*, the friable callus induced in a culture medium with 54.36 μM 2,4-D showed the highest 5-mC%, with a mean of 24.20, differing from the friable callus induced with 18.08 and 36.24 μM 2.4-D, with a mean of 12.80 and 14.60, respectively (Fig. 6c).

Global 5-mC% of *C. arabica* and *C. canephora* embryogenic calli were not influenced by 2,4-D concentrations. Furthermore, embryogenic callus of *C. canephora* had a mean value of 43.97 5-mC%, differing from *C. arabica* that exhibited a mean value of 26.78% (Fig. 6d). Regarding the 5-mC% of MCSE and ASE in *C. arabica*, the origin of friable callus influenced the response. The 5-mC% increases in the ASE according to the concentrations of 2,4-D that gave rise to friable callus. MCSE and ASE that were regenerated from friable callus of 9.06 μM 2,4-D exhibited a lower 5-mC%, with a mean of 8.91 and 11.33, and differed from ASE that were regenerated from friable callus of 36.24 and 54.36 μM 2,4-D, with a mean of 18.44 and 24.45 5-mC%, respectively. In addition, ASE regenerated from friable callus of 18.08 μM 2,4-D exhibited a mean value of 15.70 5-mC%, differing from ASE that were regenerated from friable callus of 54.36 μM 2,4-D (Fig. 6e). It was not possible to determine the level of 5-mC% in ASE of *C. canephora* that were regenerated from friable callus of 9.06, 18.08 and 36.24 μM 2,4-D, since the biological material was not sufficient (data are not shown).

**Figure 7:**
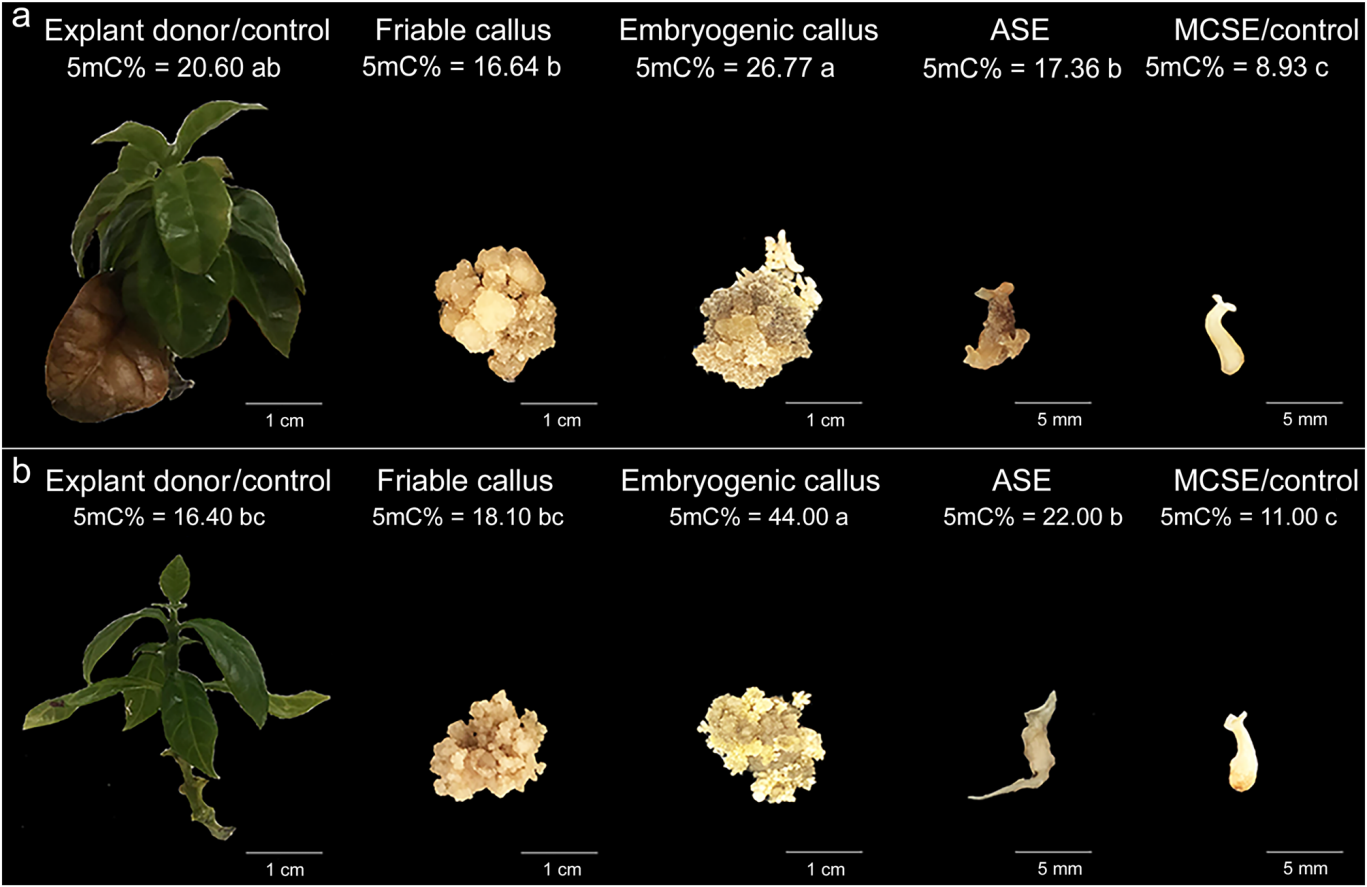
Comparison of the 5-mC% (mean values and standard deviation) in *C. arabica* (**a**) and in *C*. *canephora* (**b**) during ISE: explant donor (control), friable callus, embryogenic callus, ASE and MCSE (control in relation to the ASE). *Mean values followed by the same letter do not present a significant difference.

During ISE in *C. arabica* and *C. canephora*, in general, the level of 5-mC% varied in the different stages analyzed (friable callus, embryogenic callus, ASE and MCSE). In *C. arabica*, the explant donor (control) presented a mean value of 20.60 5-mC%. During the callus induction and proliferation step, it was observed that the 5-mC% was statistically equal, since friable callus exhibited a mean value of 16.64. In the SE induction step, a 5-mC% increase was observed as the embryogenic callus exhibited a mean value of 26.77. The regenerated ASE and MCSE (control in relation to ASE) also exhibited distinct values of 5-mC%. ASE showed a higher 5-mC%, with a mean of 17.36. Furthermore, ASE were identified due to their apical-basal pattern abnormalities, such as deformation and/or absence of the root apical meristem, hypocotyl, shoot apical meristem and/or cotyledons (Fig. 7a). In *C. canephora*, the explant donor (control) exhibited a mean value of 16.40 5-mC%. Global 5-mC% remained constant in the callus induction and proliferation stage, since the friable calluses had a mean value of 18.10. During the SE induction step there was an increase in the 5-mC%, since the embryogenic callus exhibited a mean value of 44.00. ASE exhibited higher 5-mC%, with a mean of 22.00, differing from MCSE (control in relation to ASE), which exhibited a mean value of 11.00. *C. canephora* ASE were also identified due to their apical-basal pattern abnormalities (Fig. 7b).

### Comet assay

**Figure 8:**
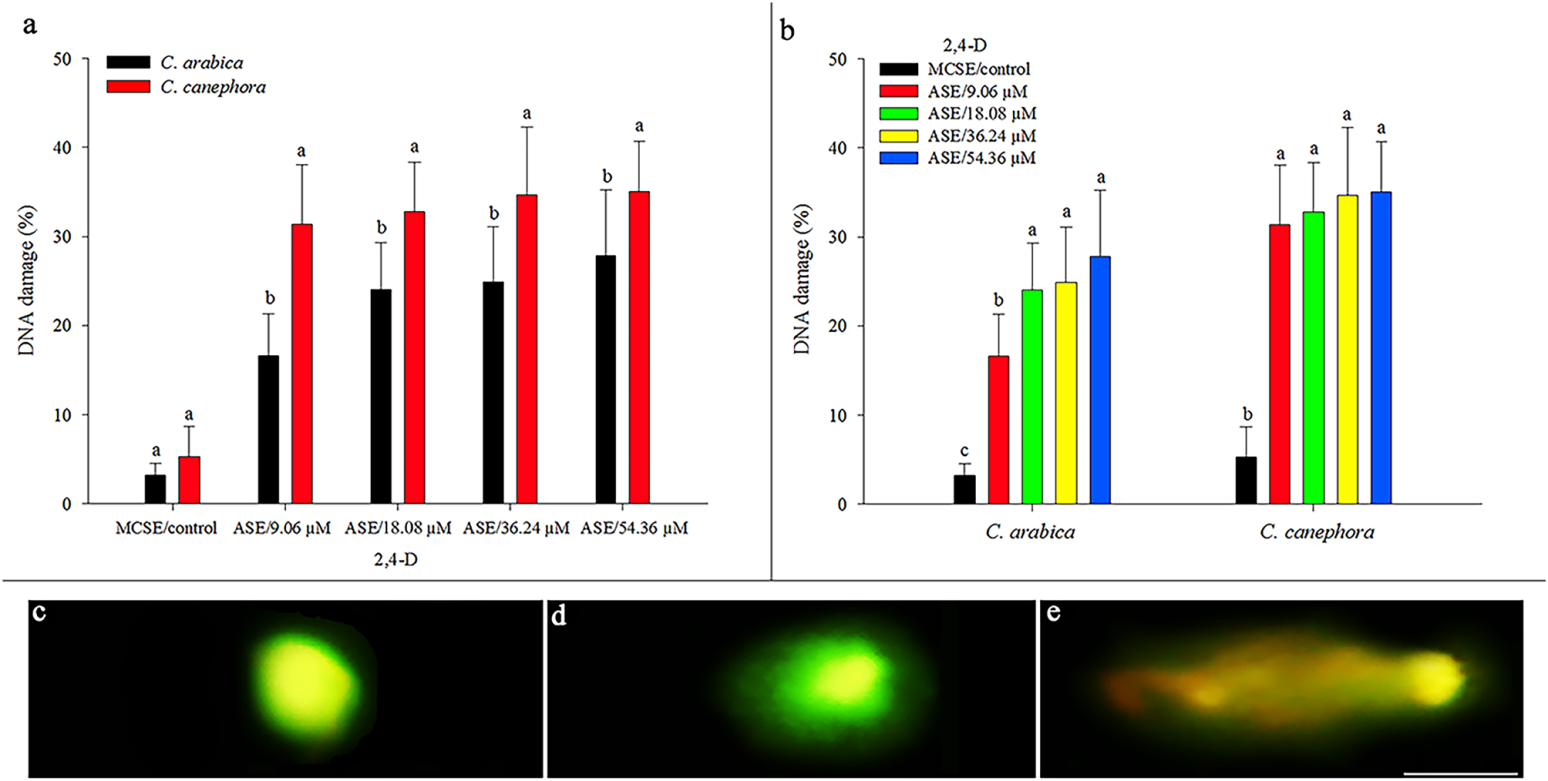
Percentage of DNA damage (mean values and standard deviation) in MCSE (control) and ASE of *C. arabica* and *C. canephora*, which are regenerated from friable callus originated from 9.06 to 54.36 μM 2,4-D. Comparison of the percentage of DNA damage between MCSE and ASE of *C. arabica* and *C. canephora* considering each 2,4-D concentration (**a**). Comparison of percentage of DNA damage in MCSE and ASE recovered from each 2,4-D concentration for *C. arabica* and *C. canephora* (**b**). Without DNA damage (**c**). With DNA damage (**d**,**e**). *Mean values followed by the same letter do not present a significant difference. Bar = 5 μm.

2,4-D had a genotoxic effect on ASE in *C. arabica* and *C. canephora*, due to the higher occurrence of DNA damage compared to MCSE (control) (Fig. 8, SI Table 5). *C. canephora* ASE showed more DNA damage than *C. arabica* ASE at all 2,4-D concentrations from which friable calluses originated. *C. canephora* was more sensitive to the 2,4-D than *C. arabica*, since *C. arabica* ASE regenerated from friable callus induced at 9.06 µM had less DNA damage (Fig. 8a). Furthermore, regenerated ASE of *C. canephora* showed the same mean DNA damage rate for friable callus induced in all 2,4-D concentrations (9.06 µM–54.36 µM). In *C. arabica*, ASE from the 9.06 μM 2,4-D exhibited less DNA damage compared to 18.08, 36.24 and 54.36 μM (Fig. 8b).

## Discussion

*C. arabica* and *C. canephora* ISE was established based on a tissue culture procedure proposed by Sanglard et al.^16^, involving the stages of callus induction and proliferation (callogenesis), followed by SE regeneration (embryogenesis). Basically, we modified the 2,4-D concentration, adding 18.08, 36.24 and 54.36 μM in the callus induction and proliferation medium. We verified the immediate 2,4-D effect in the induction and proliferation of the *C. arabica* and *C. canephora* friable callus, as well as its remaining genotoxic effect during the SE regeneration stage in the embryogenic callus from the mean number of MCSE and ASE. In addition, we measured, compared, and associated global 5-mC% with 2,4-D concentrations during the in vitro response. Our results showed that 2,4-D has a genotoxic and phytotoxic effects on SE regeneration in both species, however *C. canephora* is more sensitive to the action of 2,4-D, since ASE showed more DNA damage. Despite this, *C. canephora* requires relatively higher 2,4-D concentration than *C. arabica* to ISE establishment.

2,4-D is a synthetic auxin widely used in plant tissue culture to induce ISE^14,16^. This compound triggers the cellular transition processes from the differentiated cell to embryogenic cell. This process is complex and includes dedifferentiation and cell division, which are modulates to metabolic and developmental reprogramming, conferring competence to the cells of the explants^1^. The 2,4-D concentration and time influenced the rate and cell proliferation of friable callus of the two *Coffea*, as well as the global 5-mC%. 2,4-D induces ISE in *C. arabica* and *C. canephora* and genetic, epigenetic factors and endogenous auxin levels in the cell can influence establishment in vitro. According to Vondráková et al.^12^, endogenous auxin level in the cell and its homeostasis affect the exogenous auxin effectiveness in ISE. Homeostasis is controlled by several mechanisms, such as auxin biosynthesis, as well as its degradation, transport and conjugation^25^.

For *C. canephora*, exogenous auxin increases both free indole-3-acetic acid (IAA) and IAA amide conjugates during the callus induction, due to novel synthesis^26^. IAA is synthesized by the enzymes tryptophan aminotransferase of Arabidopsis (TAA) and YUCCA (YUC) from one of the tryptophan-dependent pathways. Uc-Chuc et al.^27^ identified 10 members of the *CcYUC* gene family in *C. canephora*. Quintana-Escobar et al.^28^ showed differences in the concentration of auxin homeostasis proteins in *C. arabica*, with a more significant accumulation of ABCs, GH3.17, UGT75C1 and IBR1 proteins. Karyotypic/genetic differences influence several traits of an individual, including the biosynthesis of auxins. *C. arabica* is an allotetraploid with 2C = 2.62 pg and 2n = 4x = 44 chromosomes. *C. canephora* is diploid with 2C = 1.41 pg and 2n = 2x = 22 chromosomes. We expected that *C. arabica* has more family members or copies of genes that encode proteins involved in auxin metabolism and, as a result, require fewer exogenous auxins. However, further investigations should be conducted to understand auxin biosynthesis in polyploids, as well as its spatial and temporal pattern of distribution during ISE.

The use of synthetic auxins, mainly 2,4-D, is necessary for the ISE induction and SE multiplication in *Coffea*, consequently potentiating the regeneration of plantlets in vitro^9,10,16^. 2,4-D can cause epigenetic and genetic changes in cells during ISE, even at very low concentrations^1,29,30^. In the present study, 2,4-D and, consequently, the global variations of 5-mC% influenced the in vitro response (acquisition of competence) in *C. arabica* and *C. canephora*. In addition, ASE regeneration was observed for both species. Corroborating with Oliveira et al.^9,10^, variations in 5-mC% in ISE in *Coffea* were associated with species and in vitro environment. The formation of ASE was related to the prolonged effect of 2,4-D, which influences the expression, development and maturation of the SE and its subsequent conversion into a plant. The long exposure or accumulation of 2,4-D in tissue interferes in establishing internal auxin gradients and inhibits cell polarization^31^. As a consequence, 2,4-D disrupts normal endogenous auxin balance and polar auxin transport, inducing embryonic abnormalities^32,33^.

In addition to 2,4-D, embryonic abnormalities may be associated with other chemical compounds required in plant tissue culture. These compounds may also cause mutations or epigenetic changes (somaclonal variation) and, consequently, influence embryonic development, SE morphology and, consequently, prevent plant recovering^17^. The formation of ASE has already been reported in *Coffea*^10,34^ and in different species such as *Medicago truncatula*^35^, *Theobroma cacao*^36^ and *Melia azedarach*^37^, being recurrent and common in plant tissue culture. For example, the ASE of the autoallohexaploid “Híbrido de Timor” (*Coffea*) exhibited the same ploidy level and chromosome number as the explant donor plants, and the high concentrations of activated charcoal in the regeneration medium has been appointed as one of the causes of these abnormalities due to phytotoxic effect^10^. The pattern of development of a normal SE in eudicotiledonea is characterized by the differentiation of a bipolar structure consisting of stem and root apex, passing through the stages of pre-embryonic and embryonic development, globular, cordiform, torpedo and cotyledonary^38^. However, ASE from *C. arabica* and *C. canephora* passed through these developmental stages, showing higher global 5-mC% and DNA damage due to the genotoxic action of 2,4-D.

DNA damage can be induced by exogenous chemical and physical factors by the action of genotoxic agents. Maintenance of the DNA integrity is necessary for the proper development of the organism and for the faithful transmission of the genetic information from one generation to the next^39^. Plants have specific mechanisms that repair DNA damage of the nuclear and organellar genomes. Our data show that *C. canephora* is more sensitive to 2,4-D, as ASE exhibited more DNA damage and higher values of 5-mC%. *C. arabica* is more tolerant to 2,4-D, probably because it is a polyploid. In addition, *C. canephora* ASE, which are regenerated from friable calli induced in all 2,4-D concentrations, showed the same mean DNA damage rate. Therefore, *Coffea* species react differently to the in vitro environment, especially to 2,4-D. As reviewed by Schifino-Wittmann^40^ and Sattler et al.^41^, polyploids have a greater buffering effect in relation to adaptability, as they have more genomic copies than diploids, and can accumulate more hidden variability. Based on this and our results, we hypothesized that the euploidy and its outcomes, as well as the evolution of the species, influence the in vitro response. However, this hypothesis should be verified in further studies. We show, for the first time, that 2,4-D promotes DNA damage during ISE and its short-term consequences in vitro, such as the formation of ASE that present morphological and/or physiological disturbances, preventing plantlet regeneration.

The addition of 2,4-D to the culture medium stimulates the induction and proliferation of friable callus in *C. arabica* and *C. canephora* and consequently increases the regeneration of SE. However, 2,4-D promotes morphological and epigenetic changes and still causes DNA damage, compromising the development of SE. Therefore, the establishment of somatic embryogenesis requires complex cellular, biochemical and molecular processes. Epigenetics plays a key role in somatic embryogenesis, as it is a genetic regulatory mechanism that influences morphogenetic processes in vitro. Maintaining the integrity of the genome of plantlets regenerated in vitro is desirable, as DNA damage can lead to loss of genetic fidelity, alter methylation patterns and cause oxidative stress, functional disturbances and cell death.

## Material and methods

*Coffea* plant materials were collected from in vitro plantlets in accordance to relevant guidelines and legislation. We declare that all methods were performed in accordance with the relevant guidelines.

### Biological material

Three plantlets of *C. arabica* and of *C. canephora* kept in vitro (Universidade Federal do Espírito Santo, Espírito Santo, Brazil) were used as explant donors. These plantlets have been cultivated in 4.3 g L^−1^ basal Murashige and Skoog (MS) salts, 10 mL L^−1^ B5 vitamins, 30 g L^−1^ sucrose, 2.8 g L^−1^ Phytagel and pH = 5.6, at 25 ± 2 °C under a 16/8 h light/dark regime with 36 μmol m^−2^ s^−1^ light radiation provided by two fluorescent lamps (20 W, Osram)^16^. These plantlets were used as control for global 5-mC%. Leaves were excised to establish the ISE and quantify 5-mC%.

### Callogenesis

Five leaf fragments (2 cm^2^) from *C. arabica* and from *C. canephora* explant donor plantlets were randomly inoculated in Petri dishes (60 × 15 mm, Olen) containing 15 mL friable callus induction medium constituted with 2.15 g L^−1^ 1/2 MS basal, 10 mL L^−1^ B5 vitamins, 30 g L^−1^ sucrose, 0.08 g L^−1^ L-cysteine, 0.4 g L^−1^ malt extract, 0.1 g L^−1^ hydrolyzed casein, 4.44 μM 6-benzylaminopurine (BAP), 2.8 g L^−1^ Phytagel, pH = 5.6, and supplemented with 9.06, 18.08, 36.24 or 54.36 μM 2,4-D. The 2,4-D concentrations were defined according to the studies about *Coffea* ISE^2,4–6,8–10,15,16,18,19^. Ten Petri dishes for each 2,4-D concentration were prepared for *C. arabica* and for *C. canephora*. The culture media were sterilized at 121 °C and 1.5 atm for 20 min. Petri dishes were maintained in the dark at 25 ± 2 °C for 90 days. Friable callus formation was evaluated biweekly, and after 90 days some samples of friable calli were collected to extract genomic DNA and determine the 5-mC%.

### Indirect somatic embryogenesis

Friable callus of *C. arabica* and *C. canephora* were individually inoculated in Petri dishes (60 × 15 mm, Olen) dishes containing 15 mL of SE regeneration medium identical to Sanglard et al.^16^, differing with 4 g L^−1^ activated charcoal, totaling 60 repetitions for *C. arabica* and 44 for *C. canephora*. Petri dishes were kept in the dark at 25 ± 2 °C for 240 days. The regeneration of MCSE and ASE was monthly evaluated. Samples of embryogenic callus, MCSE and ASE were collected to extract genomic DNA and determine the 5-mC%. In addition, the MCSE and ASE samples were used for the comet assay.

### Global 5-mC%

The collected samples (leaf from the explant donors, friable callus, embryogenic callus, MCSE and ASE) were macerated separately in the MagNALyser (Roche, Germany) for 60 s at 7000 rpm. Genomic DNA was extracted according to Doyle and Doyle^42^, with modifications adding 7.5 M ammonium acetate and excluding night precipitation. 30 μg of genomic DNA was diluted in 100 μL of dH_2_O and then acid hydrolysis was performed^9,10^. The pH of the hydrolysates was adjusted between 3 and 5 with KOH 1.0 mol L^−1^, and centrifuged at 10,000 rpm for 5 min. The supernatant was collected and transferred to a new microtube, placed in SpeedVac (Eppendorf) and subsequently stored in a refrigerator. To determine the 5-mC%, the lyophilized samples were suspended in 100 μL dH_2_O and analyzed in high performance liquid chromatography (HPLC Shimadzu, model LC-20AT) equipped with a photodiode matrix detector (SPD – M20A) using a column of reverse phase C_18_ based on silica (4.6 × 250 mm, 5 μm)^9,10^.

From 9.06, 18.08, 36.24 and 54.36 μM 2,4-D, the 5-mC% was measured for: (a) 20 samples of friable callus of *C. arabica* and *C. canephora*, totaling 5 repetitions for each treatment; (b) 12 samples of embryogenic callus of *C. arabica* and *C. canephora*, totaling 3 replicates for each treatment; (c) 12 ASE samples from *C. arabica*, totaling 3 replicates for each treatment; (d) 3 samples of *C. canephora* ASE from 54.36 μM 2,4-D because in other concentrations there was not enough biological material; (e) 3 MCSE samples from *C. arabica* and *C. canephora*; and (f) 3 samples of explant donors from *C. arabica* and *C. canephora*.

### Comet assay

Slides were immersed in normal 1% agarose at 50 °C, dried and stored in 4 °C for 24 h. The MCSE (control) and the ASE of *C. arabica* and *C. canephora* were individually collected and chopped with a razor blade in 300 μL of cell suspension solution (400 mM Tris–HCl pH 7.5, 20% polyethylene glycol). The resulting suspension was filtered through a 50, 30 and 20 µm nylon membrane. 40 µL of this cell suspension was soaked in 60 µL of low melting point agarose at a concentration of 1% at 37 °C and placed on a microscope slide pre-coated with agarose. Then, the slides were covered with a microscope coverslip (24 × 40 mm) previously cleaned and placed in a refrigeration system at 4 °C for 20 min. The coverslips were removed and incubated in a lysis solution (2.5 M NaCl; 100 mM EDTA; 10 mM Trizma base, pH = 10; 1% Triton X-100 and 10% dimethyl sulfoxide) for 1 h at 4 °C. The slides were transferred to a horizontal electrophoresis chamber containing alkaline buffer (250 mM Trizma base, 10 mM NaOH and 1 mM EDTA, pH > 13) at 4 °C, where they were incubated for 20 min for DNA unfolding and then subjected to electrophoresis at 18 V for 20 min. After electrophoresis, the slides were incubated in neutralization buffer (400 mM Tris–HCl, pH 7.5) for 15 min and then stained with 100 μL of 50 μM acridine orange for 15 min, and immersed three times in dH_2_O at 4 °C. The slides were analyzed under a 20 × objective accoupled in a fluorescence microscope Olympus BX-60 (Olympus, Japan) and nuclei images were captured with a Photometrics CoolSNAP Pro cf (Roper Scientific, Tucson, AZ). 300 nuclei per slide were evaluated, being four slides for the MCSE of *C. arabica* and *C. canephora* and 4 repetitions for the ASE of *C. arabica* and *C. canephora* regenerated from friable callus in 9.06, 18.08, 36.24 and 54.36 μM 2,4-D. *C. arabica* and *C. canephora* MCSE exhibited nuclei with DNA damage, but these observed damages, below 10%, are within the acceptable range for processing the core isolation.

### Statistical analysis

The ISE responses of *C. arabica* and *C. canephora* were compared during the callus formation and SE regeneration stages. The number of responsive explants was evaluated every 2 weeks for 90 days. The number of MCSE and ASE were evaluated monthly for 240 days. For both SE, 9.06, 36.24, 54.36 and 54.36 μM 2,4-D were considered. The data followed a normal pattern and were subjected to analysis of variance (ANOVA). The means were compared using the Tukey’s test (*P* < 0.05). A quadratic regression analysis was applied. To assess the 5-mC% and the genotoxic effect of 2,4-D during ISE, the normality of the data was verified. Then, an ANOVA was performed, taking into account the 9.06, 18.08, 36.24 and 54.36 μM 2,4-D. Mean values were compared by Tukey’s test (*P* < 0.05). For the 5-mC% and DNA damage statistical analyses, our controls were the *C. canephora* and *C. arabica* explant donors (5-mC%), and MCSE (5-mC% and DNA damage).

## Supplementary Information

Supplementary Figure 1.

Supplementary Figure 2.

Supplementary Figure 3.

Supplementary Table 1.

Supplementary Table 2.

Supplementary Table 3.

Supplementary Table 4.

Supplementary Table 5.

## Data Availability

All data generated and analyzed during this study are included in this published article and its supplementary information files (SI Table 1–SI Table 5).

## Abbreviations

ISE: Indirect somatic embryogenesis
SE: Somatic embryos
2,4-D: 2,4-Dichlorophenoxyacetic
ASE: Abnormal somatic embryos
5-mC%: Global 5-methylcytosine levels
SRAP: Sequence-related amplified polymorphism
MCSE: Mature cotyledon somatic embryo
MS: Murashige and Skoog
BAP: 6-Benzylaminopurine
