# Supplementary material for: Embryonic abnormalities and genotoxicity induced by 2,4-dichlorophenoxyacetic acid during indirect somatic embryogenesis in Coffea
Source: Sci Rep. 2023 Jun 15;13:9689. doi: 10.1038/s41598-023-36879-7 (PMC10272143; doi:10.1038/s41598-023-36879-7)
Supplement: Supplementary file 1 — Supplementary Figure 1. [file 41598_2023_36879_MOESM1_ESM.pdf]

ORIGINAL ARTICLE

Title: Embryonic abnormalities and genotoxicity induced by 2,4-dichlorophenoxyacetic acid during indirect somatic embryogenesis in *Coffea*

João Paulo de Morais Oliveira[1*], Alex Junior da Silva[2], Mariana Neves Catrinck[1], Wellington Ronildo Clarindo[2*]

[1]Laboratório de Citogenética e Cultura de Tecidos Vegetais, Centro de Ciências Agrárias e Engenharias, Universidade Federal do Espírito Santo. ZIP: 29.500-000 Alegre – ES, Brazil.
[2]Laboratório de Citogenética e Citometria, Departamento de Biologia Geral, Universidade Federal de Viçosa. ZIP: 36.570-900 Viçosa – MG, Brazil.

*Corresponding author: joaopaulo.ueg@gmail.com e well.clarindo@ufv.br

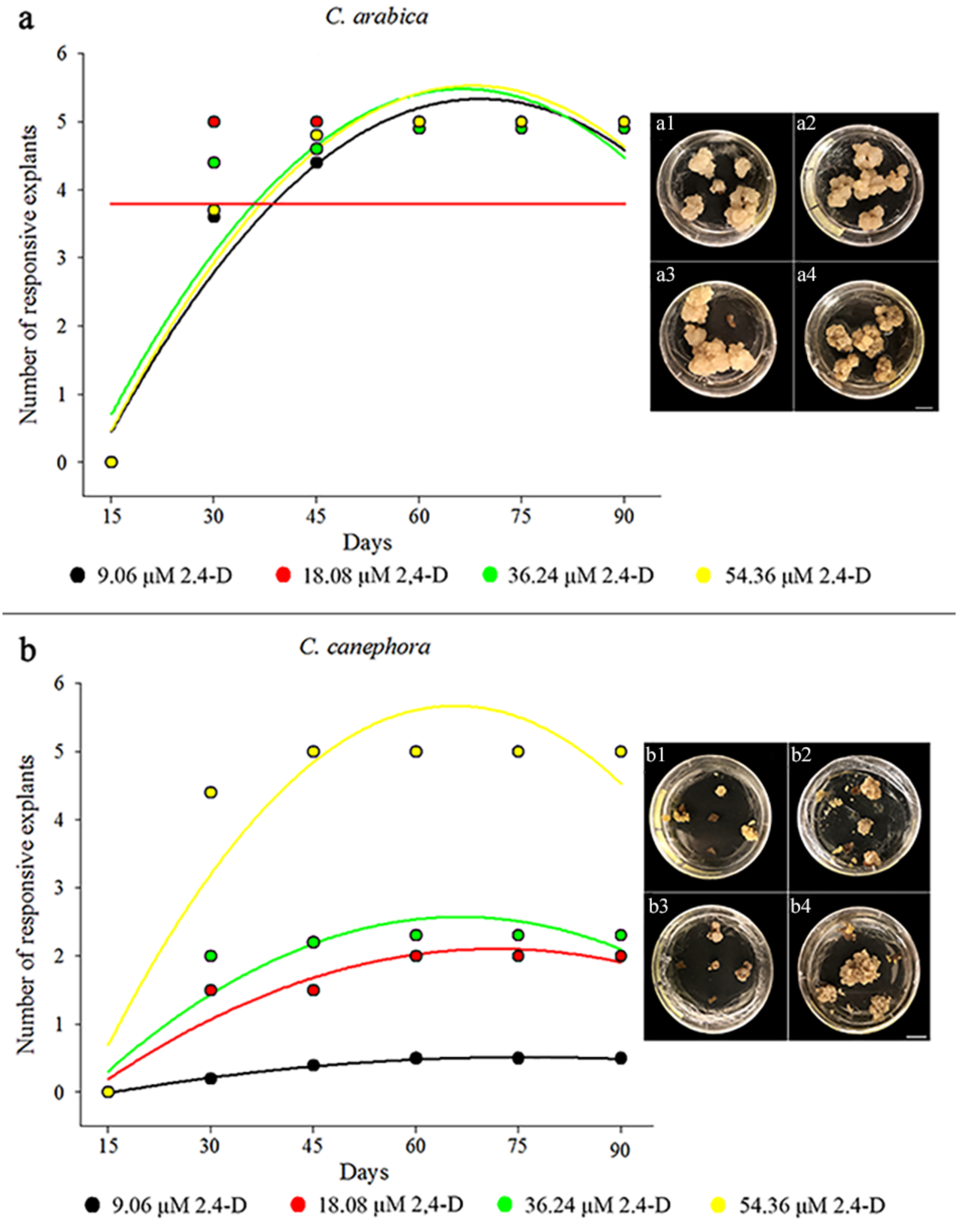

Supplementary Figure 1 – Friable callus induction in *C. arabica* and *C. canephora*. In *C. arabica*, the adjusted model was significant ($P < 0.05$) by the regression analysis for 9.06 ($Y = -0.3786X^2 + 3.4757X - 2.6400$, $R^2 = 93$), 36.24 ($Y = -0.4071X^2 + 3.6114X - 2.5800$, $R^2 = 85$) and 54.36 ($Y = -0.4089X^2 + 3.6939X - 2.8100$, $R^2 = 86$) µM 2,4-D (**a**). In *C. canephora*, the adjusted model was significant ($P < 0.05$) by the regression analysis for 9.06 ($Y = -0.0321X^2 + 0.3250X - 0.3000$, $R^2 = 99$), 18.08 ($Y = -0.1339X^2 + 1.2804X - 0.9500$, $R^2 = 91$), 36.24 ($Y = -0,1929X^2 + 1.7071X - 1.2000$, $R^2 = 87$) and 54.36 (Y = –

0,4357X^2 + 3.8157X – 2.6800, R^2 = 86) μM 2,4-D (**b**). All calli exhibited a pale-yellow color and friable appearance (**a1 – b4**). Bar = 1 cm.
