# Supplementary material for: Embryonic abnormalities and genotoxicity induced by 2,4-dichlorophenoxyacetic acid during indirect somatic embryogenesis in Coffea
Source: Sci Rep. 2023 Jun 15;13:9689. doi: 10.1038/s41598-023-36879-7 (PMC10272143; doi:10.1038/s41598-023-36879-7)
Supplement: Supplementary file 2 — Supplementary Figure 2. [file 41598_2023_36879_MOESM2_ESM.pdf]

**ORIGINAL ARTICLE**

# Title: Embryonic abnormalities and genotoxicity induced by 2,4-dichlorophenoxyacetic acid during indirect somatic embryogenesis in *Coffea*

João Paulo de Morais Oliveira[1*], Alex Junior da Silva[2], Mariana Neves Catrinck[1], Wellington Ronildo Clarindo[2*]

[1]Laboratório de Citogenética e Cultura de Tecidos Vegetais, Centro de Ciências Agrárias e Engenharias, Universidade Federal do Espírito Santo. ZIP: 29.500-000 Alegre – ES, Brazil.
[2]Laboratório de Citogenética e Citometria, Departamento de Biologia Geral, Universidade Federal de Viçosa. ZIP: 36.570-900 Viçosa – MG, Brazil.

*Corresponding author: joaopaulo.ueg@gmail.com e well.clarindo@ufv.br

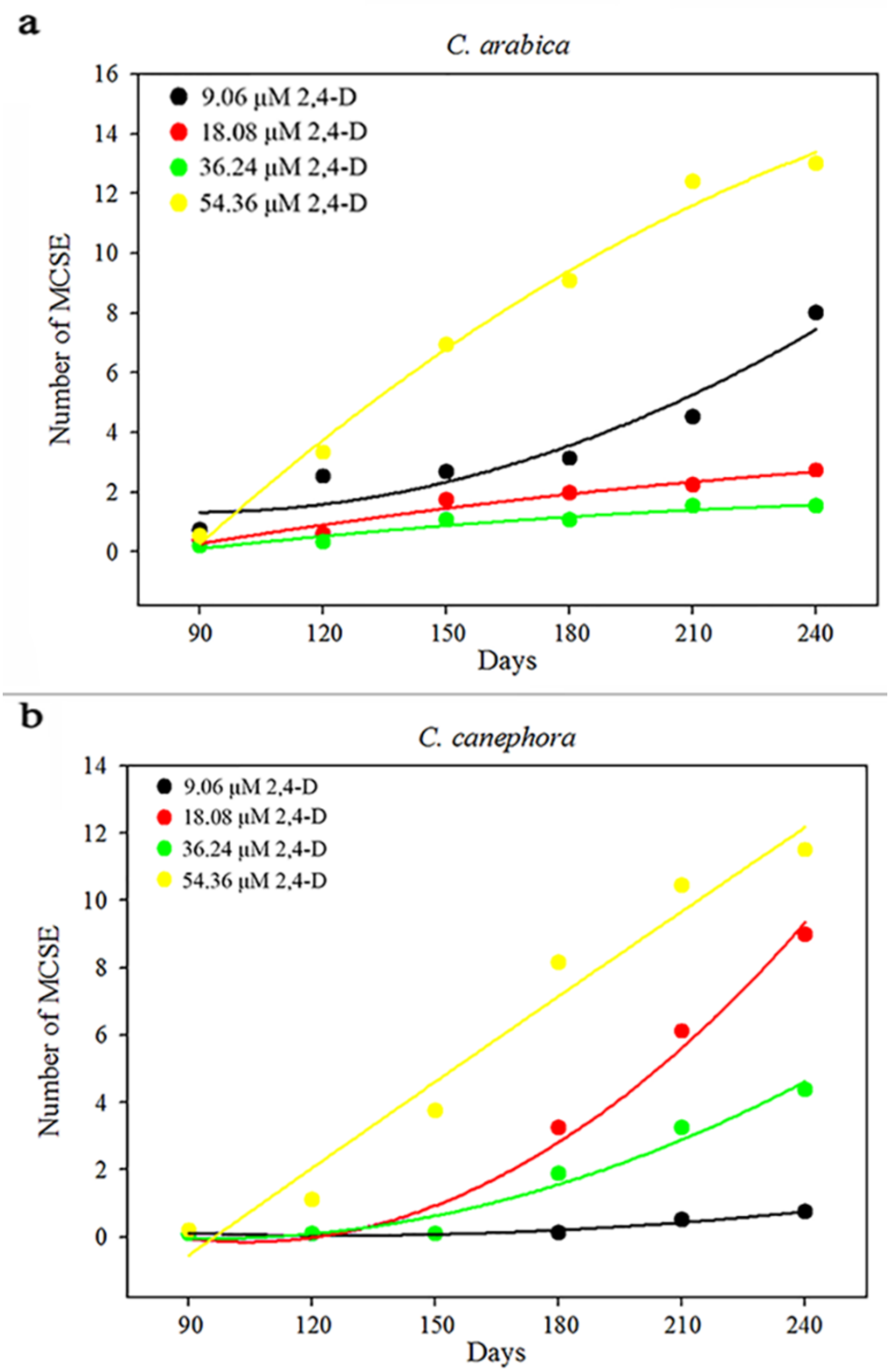


**Supplementary Figure 2** – MCSE regeneration in *C. arabica* and *C. canephora*. In *C. arabica*, the adjusted model was significant ($P < 0.05$) by the regression analysis for 9.06 ($Y = 0.2391X^2 – 0.4506X + 1.5490$, $R^2 = 92$), 18.08 ($Y = – 0.0361X^2 + 0.7322X – 0.4040$, $R^2 = 95$), 36.24 ($Y = – 0.0316X^2 + 0.5141X – 0.3650$, $R^2 = 93$) and 54.36 ($Y = – 0.2157X^2 + 4.1300X – 3.6400$, $R^2 = 99$) µM 2,4-D (**a**). In *C. canephora*, the adjusted model was significant ($P < 0.05$) by the regression analysis for 9.06 (Y =

$0.0488X^2 – 0.2133X + 0.2870$, $R^2 = 96$), 18.08 ($Y = 0.4621X^2 – 1.3576X + 0.8540$, $R^2 = 98$), 36.24 ($Y = 0.1986X^2 – 0.4594X + 0.2280$, $R^2 = 96$) and 54.36 ($Y = – 0.0116X^2 + 2.6227X – 3.1450$, $R^2 = 96$) μM 2,4-D (**b**).
