# Supplementary material for: Embryonic abnormalities and genotoxicity induced by 2,4-dichlorophenoxyacetic acid during indirect somatic embryogenesis in Coffea
Source: Sci Rep. 2023 Jun 15;13:9689. doi: 10.1038/s41598-023-36879-7 (PMC10272143; doi:10.1038/s41598-023-36879-7)
Supplement: Supplementary file 3 — Supplementary Figure 3. [file 41598_2023_36879_MOESM3_ESM.pdf]

ORIGINAL ARTICLE

Title: Embryonic abnormalities and genotoxicity induced by 2,4-dichlorophenoxyacetic acid during indirect somatic embryogenesis in *Coffea*

João Paulo de Morais Oliveira[1*], Alex Junior da Silva[2], Mariana Neves Catrinck[1], Wellington Ronildo Clarindo[2*]

[1]Laboratório de Citogenética e Cultura de Tecidos Vegetais, Centro de Ciências Agrárias e Engenharias, Universidade Federal do Espírito Santo. ZIP: 29.500-000 Alegre – ES, Brazil.
[2]Laboratório de Citogenética e Citometria, Departamento de Biologia Geral, Universidade Federal de Viçosa. ZIP: 36.570-900 Viçosa – MG, Brazil.

*Corresponding author: joaopaulo.ueg@gmail.com e well.clarindo@ufv.br

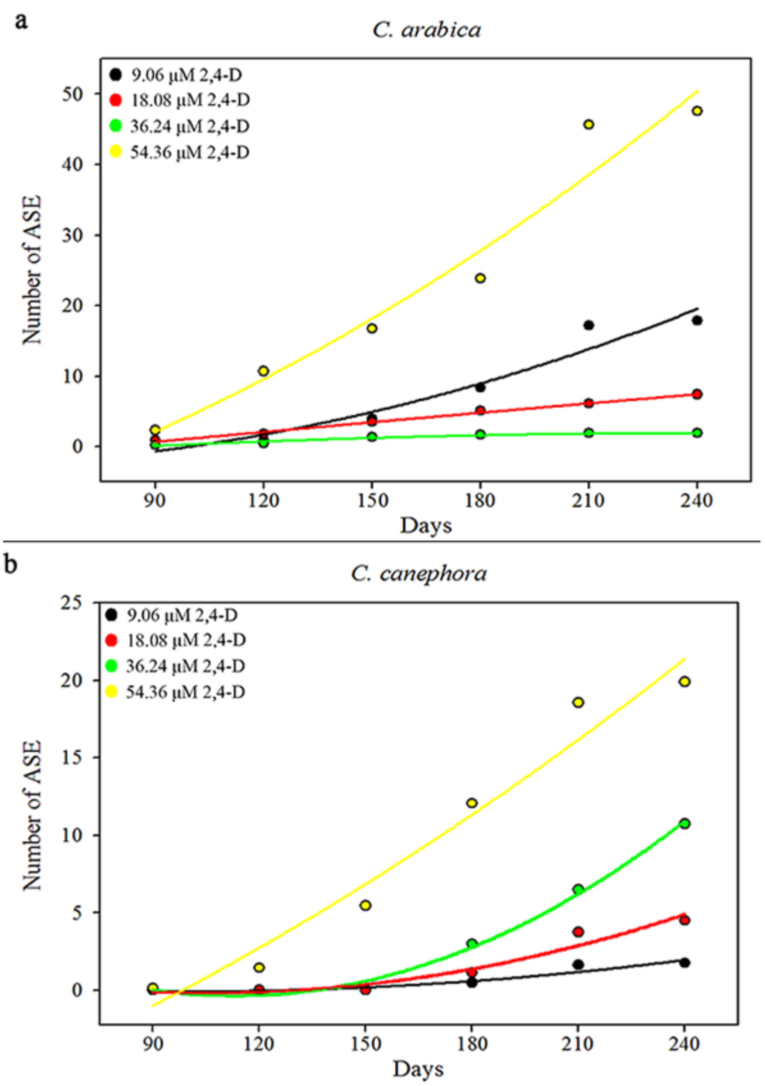

Supplementary Figure 3 – ASE regeneration from *C. arabica* and *C. canephora* friable callus. In *C. arabica*, the adjusted model was significant ($P < 0.05$) by the regression analysis for 9.06 ($Y = 0.4152X^2 + 1.1323X - 2.1950$, $R^2 = 94$), 18.08 ($Y = - 0.0123X^2 + 1.4422X - 0.7240$, $R^2 = 99$), 36.24 ($Y = - 0.0657X^2 + 0.8300X - 0.6300$, $R^2 = 95$) and 54.36 ($Y = 0.5470X^2 + 5.8264X - 4.2330$, $R^2 = 95$) µM 2,4-D (**a**). In *C. canephora*, the adjusted model was significant ($P < 0.05$) by the regression analysis for 9.06 ($Y = 0.0914X^2 - 0.2360X + 0.0860$, $R^2 = 91$), 18.08 ($Y = 0.2541X^2 - 0.7822X + 0.4470$, $R^2 = 94$), 36.24 ($Y = 0.6295X^2 - 2.2277X + 1.6250$, $R^2 = 99$) and 54.36 ($Y = 0{,}1830X^2 + 3.1945X - 4.3650$, $R^2 = 96$) µM 2,4-D (**b**).
