# Supplementary material for: Embryonic abnormalities and genotoxicity induced by 2,4-dichlorophenoxyacetic acid during indirect somatic embryogenesis in Coffea
Source: Sci Rep. 2023 Jun 15;13:9689. doi: 10.1038/s41598-023-36879-7 (PMC10272143; doi:10.1038/s41598-023-36879-7)
Supplement: Supplementary file 4 — Supplementary Table 1. [file 41598_2023_36879_MOESM4_ESM.pdf]

SI Table 1. All data generated and analysed about the friable callus induction (callogenesis) from *C. arabica* and *C. canephora* explants.

Coffea species	2,4-D (μM)	Days	Repetition	Number of Callus
C. arabica	9.06	15	1	0
C. arabica	9.06	15	2	0
C. arabica	9.06	15	3	0
C. arabica	9.06	15	4	0
C. arabica	9.06	15	5	0
C. arabica	9.06	15	6	0
C. arabica	9.06	15	7	0
C. arabica	9.06	15	8	0
C. arabica	9.06	15	9	0
C. arabica	9.06	15	10	0
C. arabica	9.06	30	1	3
C. arabica	9.06	30	2	0
C. arabica	9.06	30	3	5
C. arabica	9.06	30	4	2
C. arabica	9.06	30	5	4
C. arabica	9.06	30	6	5
C. arabica	9.06	30	7	3
C. arabica	9.06	30	8	5
C. arabica	9.06	30	9	4
C. arabica	9.06	30	10	5
C. arabica	9.06	45	1	3
C. arabica	9.06	45	2	1
C. arabica	9.06	45	3	5
C. arabica	9.06	45	4	5
C. arabica	9.06	45	5	5
C. arabica	9.06	45	6	5
C. arabica	9.06	45	7	5
C. arabica	9.06	45	8	5
C. arabica	9.06	45	9	5
C. arabica	9.06	45	10	5
C. arabica	9.06	60	1	4
C. arabica	9.06	60	2	5
C. arabica	9.06	60	3	5
C. arabica	9.06	60	4	5
C. arabica	9.06	60	5	5
C. arabica	9.06	60	6	5
C. arabica	9.06	60	7	5
C. arabica	9.06	60	8	5
C. arabica	9.06	60	9	5
C. arabica	9.06	60	10	5
C. arabica	9.06	75	1	4
C. arabica	9.06	75	2	5
C. arabica	9.06	75	3	5
C. arabica	9.06	75	4	5
C. arabica	9.06	75	5	5

C. arabica	9.06	75	6	5
C. arabica	9.06	75	7	5
C. arabica	9.06	75	8	5
C. arabica	9.06	75	9	5
C. arabica	9.06	75	10	5
C. arabica	9.06	90	1	4
C. arabica	9.06	90	2	5
C. arabica	9.06	90	3	5
C. arabica	9.06	90	4	5
C. arabica	9.06	90	5	5
C. arabica	9.06	90	6	5
C. arabica	9.06	90	7	5
C. arabica	9.06	90	8	5
C. arabica	9.06	90	9	5
C. arabica	9.06	90	10	5
C. arabica	18.08	15	1	0
C. arabica	18.08	15	2	0
C. arabica	18.08	15	3	0
C. arabica	18.08	15	4	0
C. arabica	18.08	15	5	0
C. arabica	18.08	15	6	0
C. arabica	18.08	15	7	0
C. arabica	18.08	15	8	0
C. arabica	18.08	15	9	0
C. arabica	18.08	15	10	0
C. arabica	18.08	30	1	5
C. arabica	18.08	30	2	5
C. arabica	18.08	30	3	5
C. arabica	18.08	30	4	5
C. arabica	18.08	30	5	5
C. arabica	18.08	30	6	5
C. arabica	18.08	30	7	5
C. arabica	18.08	30	8	5
C. arabica	18.08	30	9	5
C. arabica	18.08	30	10	5
C. arabica	18.08	45	1	5
C. arabica	18.08	45	2	5
C. arabica	18.08	45	3	5
C. arabica	18.08	45	4	5
C. arabica	18.08	45	5	5
C. arabica	18.08	45	6	5
C. arabica	18.08	45	7	5
C. arabica	18.08	45	8	5
C. arabica	18.08	45	9	5
C. arabica	18.08	45	10	5
C. arabica	18.08	60	1	5
C. arabica	18.08	60	2	5
C. arabica	18.08	60	3	5
C. arabica	18.08	60	4	5

C. arabica	18.08	60	5	5
C. arabica	18.08	60	6	5
C. arabica	18.08	60	7	5
C. arabica	18.08	60	8	5
C. arabica	18.08	60	9	5
C. arabica	18.08	60	10	5
C. arabica	18.08	75	1	5
C. arabica	18.08	75	2	5
C. arabica	18.08	75	3	5
C. arabica	18.08	75	4	5
C. arabica	18.08	75	5	5
C. arabica	18.08	75	6	5
C. arabica	18.08	75	7	5
C. arabica	18.08	75	8	5
C. arabica	18.08	75	9	5
C. arabica	18.08	75	10	5
C. arabica	18.08	90	1	5
C. arabica	18.08	90	2	5
C. arabica	18.08	90	3	5
C. arabica	18.08	90	4	5
C. arabica	18.08	90	5	5
C. arabica	18.08	90	6	5
C. arabica	18.08	90	7	5
C. arabica	18.08	90	8	5
C. arabica	18.08	90	9	5
C. arabica	18.08	90	10	5
C. arabica	36.24	15	1	0
C. arabica	36.24	15	2	0
C. arabica	36.24	15	3	0
C. arabica	36.24	15	4	0
C. arabica	36.24	15	5	0
C. arabica	36.24	15	6	0
C. arabica	36.24	15	7	0
C. arabica	36.24	15	8	0
C. arabica	36.24	15	9	0
C. arabica	36.24	15	10	0
C. arabica	36.24	30	1	4
C. arabica	36.24	30	2	5
C. arabica	36.24	30	3	5
C. arabica	36.24	30	4	5
C. arabica	36.24	30	5	5
C. arabica	36.24	30	6	3
C. arabica	36.24	30	7	5
C. arabica	36.24	30	8	5
C. arabica	36.24	30	9	2
C. arabica	36.24	30	10	5
C. arabica	36.24	45	1	5
C. arabica	36.24	45	2	5
C. arabica	36.24	45	3	5

C. arabica	36.24	45	4	5
C. arabica	36.24	45	5	5
C. arabica	36.24	45	6	3
C. arabica	36.24	45	7	5
C. arabica	36.24	45	8	5
C. arabica	36.24	45	9	3
C. arabica	36.24	45	10	5
C. arabica	36.24	60	1	5
C. arabica	36.24	60	2	5
C. arabica	36.24	60	3	5
C. arabica	36.24	60	4	5
C. arabica	36.24	60	5	5
C. arabica	36.24	60	6	4
C. arabica	36.24	60	7	5
C. arabica	36.24	60	8	5
C. arabica	36.24	60	9	5
C. arabica	36.24	60	10	5
C. arabica	36.24	75	1	5
C. arabica	36.24	75	2	5
C. arabica	36.24	75	3	5
C. arabica	36.24	75	4	5
C. arabica	36.24	75	5	5
C. arabica	36.24	75	6	4
C. arabica	36.24	75	7	5
C. arabica	36.24	75	8	5
C. arabica	36.24	75	9	5
C. arabica	36.24	75	10	5
C. arabica	36.24	90	1	5
C. arabica	36.24	90	2	5
C. arabica	36.24	90	3	5
C. arabica	36.24	90	4	5
C. arabica	36.24	90	5	5
C. arabica	36.24	90	6	4
C. arabica	36.24	90	7	5
C. arabica	36.24	90	8	5
C. arabica	36.24	90	9	5
C. arabica	36.24	90	10	5
C. arabica	54.36	15	1	0
C. arabica	54.36	15	2	0
C. arabica	54.36	15	3	0
C. arabica	54.36	15	4	0
C. arabica	54.36	15	5	0
C. arabica	54.36	15	6	0
C. arabica	54.36	15	7	0
C. arabica	54.36	15	8	0
C. arabica	54.36	15	9	0
C. arabica	54.36	15	10	0
C. arabica	54.36	30	1	3
C. arabica	54.36	30	2	3

C. arabica	54.36	30	3	4
C. arabica	54.36	30	4	4
C. arabica	54.36	30	5	2
C. arabica	54.36	30	6	5
C. arabica	54.36	30	7	5
C. arabica	54.36	30	8	3
C. arabica	54.36	30	9	4
C. arabica	54.36	30	10	4
C. arabica	54.36	45	1	5
C. arabica	54.36	45	2	5
C. arabica	54.36	45	3	5
C. arabica	54.36	45	4	5
C. arabica	54.36	45	5	3
C. arabica	54.36	45	6	5
C. arabica	54.36	45	7	5
C. arabica	54.36	45	8	5
C. arabica	54.36	45	9	5
C. arabica	54.36	45	10	5
C. arabica	54.36	60	1	5
C. arabica	54.36	60	2	5
C. arabica	54.36	60	3	5
C. arabica	54.36	60	4	5
C. arabica	54.36	60	5	5
C. arabica	54.36	60	6	5
C. arabica	54.36	60	7	5
C. arabica	54.36	60	8	5
C. arabica	54.36	60	9	5
C. arabica	54.36	60	10	5
C. arabica	54.36	75	1	5
C. arabica	54.36	75	2	5
C. arabica	54.36	75	3	5
C. arabica	54.36	75	4	5
C. arabica	54.36	75	5	5
C. arabica	54.36	75	6	5
C. arabica	54.36	75	7	5
C. arabica	54.36	75	8	5
C. arabica	54.36	75	9	5
C. arabica	54.36	75	10	5
C. arabica	54.36	90	1	5
C. arabica	54.36	90	2	5
C. arabica	54.36	90	3	5
C. arabica	54.36	90	4	5
C. arabica	54.36	90	5	5
C. arabica	54.36	90	6	5
C. arabica	54.36	90	7	5
C. arabica	54.36	90	8	5
C. arabica	54.36	90	9	5
C. arabica	54.36	90	10	5
C. canephora	9.06	15	1	0

C. canephora	9.06	15	2	0
C. canephora	9.06	15	3	0
C. canephora	9.06	15	4	0
C. canephora	9.06	15	5	0
C. canephora	9.06	15	6	0
C. canephora	9.06	15	7	0
C. canephora	9.06	15	8	0
C. canephora	9.06	15	9	0
C. canephora	9.06	15	10	0
C. canephora	9.06	30	1	0
C. canephora	9.06	30	2	0
C. canephora	9.06	30	3	0
C. canephora	9.06	30	4	0
C. canephora	9.06	30	5	0
C. canephora	9.06	30	6	0
C. canephora	9.06	30	7	0
C. canephora	9.06	30	8	0
C. canephora	9.06	30	9	1
C. canephora	9.06	30	10	1
C. canephora	9.06	45	1	0
C. canephora	9.06	45	2	0
C. canephora	9.06	45	3	0
C. canephora	9.06	45	4	0
C. canephora	9.06	45	5	0
C. canephora	9.06	45	6	0
C. canephora	9.06	45	7	0
C. canephora	9.06	45	8	0
C. canephora	9.06	45	9	2
C. canephora	9.06	45	10	2
C. canephora	9.06	60	1	0
C. canephora	9.06	60	2	0
C. canephora	9.06	60	3	0
C. canephora	9.06	60	4	0
C. canephora	9.06	60	5	0
C. canephora	9.06	60	6	0
C. canephora	9.06	60	7	0
C. canephora	9.06	60	8	0
C. canephora	9.06	60	9	2
C. canephora	9.06	60	10	3
C. canephora	9.06	75	1	0
C. canephora	9.06	75	2	0
C. canephora	9.06	75	3	0
C. canephora	9.06	75	4	0
C. canephora	9.06	75	5	0
C. canephora	9.06	75	6	0
C. canephora	9.06	75	7	0
C. canephora	9.06	75	8	0
C. canephora	9.06	75	9	2
C. canephora	9.06	75	10	3

C. canephora	9.06	90	1	0
C. canephora	9.06	90	2	0
C. canephora	9.06	90	3	0
C. canephora	9.06	90	4	0
C. canephora	9.06	90	5	0
C. canephora	9.06	90	6	0
C. canephora	9.06	90	7	0
C. canephora	9.06	90	8	0
C. canephora	9.06	90	9	2
C. canephora	9.06	90	10	3
C. canephora	18.08	15	1	0
C. canephora	18.08	15	2	0
C. canephora	18.08	15	3	0
C. canephora	18.08	15	4	0
C. canephora	18.08	15	5	0
C. canephora	18.08	15	6	0
C. canephora	18.08	15	7	0
C. canephora	18.08	15	8	0
C. canephora	18.08	15	9	0
C. canephora	18.08	15	10	0
C. canephora	18.08	30	1	2
C. canephora	18.08	30	2	0
C. canephora	18.08	30	3	2
C. canephora	18.08	30	4	2
C. canephora	18.08	30	5	0
C. canephora	18.08	30	6	0
C. canephora	18.08	30	7	2
C. canephora	18.08	30	8	3
C. canephora	18.08	30	9	3
C. canephora	18.08	30	10	1
C. canephora	18.08	45	1	2
C. canephora	18.08	45	2	0
C. canephora	18.08	45	3	2
C. canephora	18.08	45	4	2
C. canephora	18.08	45	5	0
C. canephora	18.08	45	6	0
C. canephora	18.08	45	7	2
C. canephora	18.08	45	8	3
C. canephora	18.08	45	9	3
C. canephora	18.08	45	10	1
C. canephora	18.08	60	1	2
C. canephora	18.08	60	2	0
C. canephora	18.08	60	3	2
C. canephora	18.08	60	4	3
C. canephora	18.08	60	5	1
C. canephora	18.08	60	6	0
C. canephora	18.08	60	7	3
C. canephora	18.08	60	8	4
C. canephora	18.08	60	9	3

C. canephora	18.08	60	10	2
C. canephora	18.08	75	1	2
C. canephora	18.08	75	2	0
C. canephora	18.08	75	3	2
C. canephora	18.08	75	4	3
C. canephora	18.08	75	5	1
C. canephora	18.08	75	6	0
C. canephora	18.08	75	7	3
C. canephora	18.08	75	8	4
C. canephora	18.08	75	9	3
C. canephora	18.08	75	10	2
C. canephora	18.08	90	1	2
C. canephora	18.08	90	2	0
C. canephora	18.08	90	3	2
C. canephora	18.08	90	4	3
C. canephora	18.08	90	5	1
C. canephora	18.08	90	6	0
C. canephora	18.08	90	7	3
C. canephora	18.08	90	8	4
C. canephora	18.08	90	9	3
C. canephora	18.08	90	10	2
C. canephora	36.24	15	1	0
C. canephora	36.24	15	2	0
C. canephora	36.24	15	3	0
C. canephora	36.24	15	4	0
C. canephora	36.24	15	5	0
C. canephora	36.24	15	6	0
C. canephora	36.24	15	7	0
C. canephora	36.24	15	8	0
C. canephora	36.24	15	9	0
C. canephora	36.24	15	10	0
C. canephora	36.24	30	1	0
C. canephora	36.24	30	2	0
C. canephora	36.24	30	3	0
C. canephora	36.24	30	4	3
C. canephora	36.24	30	5	0
C. canephora	36.24	30	6	4
C. canephora	36.24	30	7	0
C. canephora	36.24	30	8	5
C. canephora	36.24	30	9	5
C. canephora	36.24	30	10	5
C. canephora	36.24	45	1	0
C. canephora	36.24	45	2	0
C. canephora	36.24	45	3	0
C. canephora	36.24	45	4	4
C. canephora	36.24	45	5	0
C. canephora	36.24	45	6	4
C. canephora	36.24	45	7	0
C. canephora	36.24	45	8	5

C. canephora	36.24	45	9	5
C. canephora	36.24	45	10	5
C. canephora	36.24	60	1	0
C. canephora	36.24	60	2	0
C. canephora	36.24	60	3	0
C. canephora	36.24	60	4	4
C. canephora	36.24	60	5	0
C. canephora	36.24	60	6	4
C. canephora	36.24	60	7	0
C. canephora	36.24	60	8	5
C. canephora	36.24	60	9	5
C. canephora	36.24	60	10	5
C. canephora	36.24	75	1	0
C. canephora	36.24	75	2	0
C. canephora	36.24	75	3	0
C. canephora	36.24	75	4	4
C. canephora	36.24	75	5	0
C. canephora	36.24	75	6	4
C. canephora	36.24	75	7	0
C. canephora	36.24	75	8	5
C. canephora	36.24	75	9	5
C. canephora	36.24	75	10	5
C. canephora	36.24	90	1	0
C. canephora	36.24	90	2	0
C. canephora	36.24	90	3	0
C. canephora	36.24	90	4	4
C. canephora	36.24	90	5	0
C. canephora	36.24	90	6	4
C. canephora	36.24	90	7	0
C. canephora	36.24	90	8	5
C. canephora	36.24	90	9	5
C. canephora	36.24	90	10	5
C. canephora	54.36	15	1	0
C. canephora	54.36	15	2	0
C. canephora	54.36	15	3	0
C. canephora	54.36	15	4	0
C. canephora	54.36	15	5	0
C. canephora	54.36	15	6	0
C. canephora	54.36	15	7	0
C. canephora	54.36	15	8	0
C. canephora	54.36	15	9	0
C. canephora	54.36	15	10	0
C. canephora	54.36	30	1	4
C. canephora	54.36	30	2	3
C. canephora	54.36	30	3	5
C. canephora	54.36	30	4	5
C. canephora	54.36	30	5	4
C. canephora	54.36	30	6	5
C. canephora	54.36	30	7	4

C. canephora	54.36	30	8	5
C. canephora	54.36	30	9	5
C. canephora	54.36	30	10	4
C. canephora	54.36	45	1	5
C. canephora	54.36	45	2	5
C. canephora	54.36	45	3	5
C. canephora	54.36	45	4	5
C. canephora	54.36	45	5	5
C. canephora	54.36	45	6	5
C. canephora	54.36	45	7	5
C. canephora	54.36	45	8	5
C. canephora	54.36	45	9	5
C. canephora	54.36	45	10	5
C. canephora	54.36	60	1	5
C. canephora	54.36	60	2	5
C. canephora	54.36	60	3	5
C. canephora	54.36	60	4	5
C. canephora	54.36	60	5	5
C. canephora	54.36	60	6	5
C. canephora	54.36	60	7	5
C. canephora	54.36	60	8	5
C. canephora	54.36	60	9	5
C. canephora	54.36	60	10	5
C. canephora	54.36	75	1	5
C. canephora	54.36	75	2	5
C. canephora	54.36	75	3	5
C. canephora	54.36	75	4	5
C. canephora	54.36	75	5	5
C. canephora	54.36	75	6	5
C. canephora	54.36	75	7	5
C. canephora	54.36	75	8	5
C. canephora	54.36	75	9	5
C. canephora	54.36	75	10	5
C. canephora	54.36	90	1	5
C. canephora	54.36	90	2	5
C. canephora	54.36	90	3	5
C. canephora	54.36	90	4	5
C. canephora	54.36	90	5	5
C. canephora	54.36	90	6	5
C. canephora	54.36	90	7	5
C. canephora	54.36	90	8	5
C. canephora	54.36	90	9	5
C. canephora	54.36	90	10	5
