# Supplementary material for: Embryonic abnormalities and genotoxicity induced by 2,4-dichlorophenoxyacetic acid during indirect somatic embryogenesis in Coffea
Source: Sci Rep. 2023 Jun 15;13:9689. doi: 10.1038/s41598-023-36879-7 (PMC10272143; doi:10.1038/s41598-023-36879-7)
Supplement: Supplementary file 5 — Supplementary Table 2. [file 41598_2023_36879_MOESM5_ESM.pdf]

SI Table 2. All data generated and analysed about the mature cotyledon somatic embryo (MCSE) regenerated from *C. arabica* and *C. canephora* friable callus.

Coffea species	2,4-D (μM)	Days	Repetition	MCSE	Adjusted data
C. arabica	9.06	30	1	0	0.707106781
C. arabica	9.06	30	2	0	0.707106781
C. arabica	9.06	30	3	0	0.707106781
C. arabica	9.06	30	4	0	0.707106781
C. arabica	9.06	30	5	0	0.707106781
C. arabica	9.06	30	6	0	0.707106781
C. arabica	9.06	30	7	0	0.707106781
C. arabica	9.06	30	8	0	0.707106781
C. arabica	9.06	30	9	0	0.707106781
C. arabica	9.06	30	10	0	0.707106781
C. arabica	9.06	30	11	0	0.707106781
C. arabica	9.06	30	12	0	0.707106781
C. arabica	9.06	30	13	0	0.707106781
C. arabica	9.06	30	14	0	0.707106781
C. arabica	9.06	30	15	0	0.707106781
C. arabica	9.06	60	1	0	0.707106781
C. arabica	9.06	60	2	0	0.707106781
C. arabica	9.06	60	3	0	0.707106781
C. arabica	9.06	60	4	0	0.707106781
C. arabica	9.06	60	5	0	0.707106781
C. arabica	9.06	60	6	0	0.707106781
C. arabica	9.06	60	7	0	0.707106781
C. arabica	9.06	60	8	0	0.707106781
C. arabica	9.06	60	9	0	0.707106781
C. arabica	9.06	60	10	1	1.224744871
C. arabica	9.06	60	11	0	0.707106781
C. arabica	9.06	60	12	0	0.707106781
C. arabica	9.06	60	13	0	0.707106781
C. arabica	9.06	60	14	0	0.707106781
C. arabica	9.06	60	15	1	1.224744871
C. arabica	9.06	90	1	0	0.707106781
C. arabica	9.06	90	2	0	0.707106781
C. arabica	9.06	90	3	1	1.224744871
C. arabica	9.06	90	4	1	1.224744871
C. arabica	9.06	90	5	0	0.707106781
C. arabica	9.06	90	6	0	0.707106781
C. arabica	9.06	90	7	1	1.224744871
C. arabica	9.06	90	8	1	1.224744871
C. arabica	9.06	90	9	1	1.224744871
C. arabica	9.06	90	10	2	1.58113883
C. arabica	9.06	90	11	1	1.224744871
C. arabica	9.06	90	12	0	0.707106781
C. arabica	9.06	90	13	0	0.707106781
C. arabica	9.06	90	14	1	1.224744871
C. arabica	9.06	90	15	2	1.58113883
C. arabica	9.06	120	1	1	1.224744871

C. arabica	9.06	120	2	1	1.224744871
C. arabica	9.06	120	3	1	1.224744871
C. arabica	9.06	120	4	1	1.224744871
C. arabica	9.06	120	5	1	1.224744871
C. arabica	9.06	120	6	2	1.58113883
C. arabica	9.06	120	7	3	1.870828693
C. arabica	9.06	120	8	2	1.58113883
C. arabica	9.06	120	9	5	2.34520788
C. arabica	9.06	120	10	5	2.34520788
C. arabica	9.06	120	11	2	1.58113883
C. arabica	9.06	120	12	5	2.34520788
C. arabica	9.06	120	13	3	1.870828693
C. arabica	9.06	120	14	3	1.870828693
C. arabica	9.06	120	15	3	1.870828693
C. arabica	9.06	150	1	1	1.224744871
C. arabica	9.06	150	2	1	1.224744871
C. arabica	9.06	150	3	1	1.224744871
C. arabica	9.06	150	4	2	1.58113883
C. arabica	9.06	150	5	2	1.58113883
C. arabica	9.06	150	6	3	1.870828693
C. arabica	9.06	150	7	3	1.870828693
C. arabica	9.06	150	8	3	1.870828693
C. arabica	9.06	150	9	4	2.121320344
C. arabica	9.06	150	10	4	2.121320344
C. arabica	9.06	150	11	3	1.870828693
C. arabica	9.06	150	12	4	2.121320344
C. arabica	9.06	150	13	3	1.870828693
C. arabica	9.06	150	14	3	1.870828693
C. arabica	9.06	150	15	3	1.870828693
C. arabica	9.06	180	1	2	1.58113883
C. arabica	9.06	180	2	3	1.870828693
C. arabica	9.06	180	3	3	1.870828693
C. arabica	9.06	180	4	3	1.870828693
C. arabica	9.06	180	5	3	1.870828693
C. arabica	9.06	180	6	3	1.870828693
C. arabica	9.06	180	7	3	1.870828693
C. arabica	9.06	180	8	3	1.870828693
C. arabica	9.06	180	9	4	2.121320344
C. arabica	9.06	180	10	4	2.121320344
C. arabica	9.06	180	11	3	1.870828693
C. arabica	9.06	180	12	4	2.121320344
C. arabica	9.06	180	13	3	1.870828693
C. arabica	9.06	180	14	3	1.870828693
C. arabica	9.06	180	15	3	1.870828693
C. arabica	9.06	210	1	4	2.121320344
C. arabica	9.06	210	2	4	2.121320344
C. arabica	9.06	210	3	4	2.121320344
C. arabica	9.06	210	4	4	2.121320344
C. arabica	9.06	210	5	4	2.121320344

C. arabica	9.06	210	6	4	2.121320344
C. arabica	9.06	210	7	5	2.34520788
C. arabica	9.06	210	8	5	2.34520788
C. arabica	9.06	210	9	5	2.34520788
C. arabica	9.06	210	10	5	2.34520788
C. arabica	9.06	210	11	5	2.34520788
C. arabica	9.06	210	12	5	2.34520788
C. arabica	9.06	210	13	4	2.121320344
C. arabica	9.06	210	14	5	2.34520788
C. arabica	9.06	210	15	5	2.34520788
C. arabica	9.06	240	1	7	2.738612788
C. arabica	9.06	240	2	7	2.738612788
C. arabica	9.06	240	3	7	2.738612788
C. arabica	9.06	240	4	7	2.738612788
C. arabica	9.06	240	5	9	3.082207001
C. arabica	9.06	240	6	9	3.082207001
C. arabica	9.06	240	7	7	2.738612788
C. arabica	9.06	240	8	8	2.915475947
C. arabica	9.06	240	9	9	3.082207001
C. arabica	9.06	240	10	9	3.082207001
C. arabica	9.06	240	11	8	2.915475947
C. arabica	9.06	240	12	8	2.915475947
C. arabica	9.06	240	13	8	2.915475947
C. arabica	9.06	240	14	8	2.915475947
C. arabica	9.06	240	15	9	3.082207001
C. arabica	18.08	30	1	0	0.707106781
C. arabica	18.08	30	2	0	0.707106781
C. arabica	18.08	30	3	0	0.707106781
C. arabica	18.08	30	4	0	0.707106781
C. arabica	18.08	30	5	0	0.707106781
C. arabica	18.08	30	6	0	0.707106781
C. arabica	18.08	30	7	0	0.707106781
C. arabica	18.08	30	8	0	0.707106781
C. arabica	18.08	30	9	0	0.707106781
C. arabica	18.08	30	10	0	0.707106781
C. arabica	18.08	30	11	0	0.707106781
C. arabica	18.08	30	12	0	0.707106781
C. arabica	18.08	30	13	0	0.707106781
C. arabica	18.08	30	14	0	0.707106781
C. arabica	18.08	30	15	0	0.707106781
C. arabica	18.08	60	1	0	0.707106781
C. arabica	18.08	60	2	0	0.707106781
C. arabica	18.08	60	3	0	0.707106781
C. arabica	18.08	60	4	0	0.707106781
C. arabica	18.08	60	5	0	0.707106781
C. arabica	18.08	60	6	0	0.707106781
C. arabica	18.08	60	7	0	0.707106781
C. arabica	18.08	60	8	0	0.707106781
C. arabica	18.08	60	9	0	0.707106781

C. arabica	18.08	60	10	0	0.707106781
C. arabica	18.08	60	11	0	0.707106781
C. arabica	18.08	60	12	0	0.707106781
C. arabica	18.08	60	13	0	0.707106781
C. arabica	18.08	60	14	0	0.707106781
C. arabica	18.08	60	15	0	0.707106781
C. arabica	18.08	90	1	0	0.707106781
C. arabica	18.08	90	2	0	0.707106781
C. arabica	18.08	90	3	0	0.707106781
C. arabica	18.08	90	4	0	0.707106781
C. arabica	18.08	90	5	0	0.707106781
C. arabica	18.08	90	6	0	0.707106781
C. arabica	18.08	90	7	0	0.707106781
C. arabica	18.08	90	8	0	0.707106781
C. arabica	18.08	90	9	0	0.707106781
C. arabica	18.08	90	10	0	0.707106781
C. arabica	18.08	90	11	1	1.224744871
C. arabica	18.08	90	12	1	1.224744871
C. arabica	18.08	90	13	1	1.224744871
C. arabica	18.08	90	14	2	1.58113883
C. arabica	18.08	90	15	1	1.224744871
C. arabica	18.08	120	1	0	0.707106781
C. arabica	18.08	120	2	0	0.707106781
C. arabica	18.08	120	3	0	0.707106781
C. arabica	18.08	120	4	0	0.707106781
C. arabica	18.08	120	5	0	0.707106781
C. arabica	18.08	120	6	0	0.707106781
C. arabica	18.08	120	7	0	0.707106781
C. arabica	18.08	120	8	0	0.707106781
C. arabica	18.08	120	9	1	1.224744871
C. arabica	18.08	120	10	1	1.224744871
C. arabica	18.08	120	11	1	1.224744871
C. arabica	18.08	120	12	1	1.224744871
C. arabica	18.08	120	13	1	1.224744871
C. arabica	18.08	120	14	2	1.58113883
C. arabica	18.08	120	15	2	1.58113883
C. arabica	18.08	150	1	1	1.224744871
C. arabica	18.08	150	2	1	1.224744871
C. arabica	18.08	150	3	2	1.58113883
C. arabica	18.08	150	4	0	0.707106781
C. arabica	18.08	150	5	0	0.707106781
C. arabica	18.08	150	6	1	1.224744871
C. arabica	18.08	150	7	2	1.58113883
C. arabica	18.08	150	8	2	1.58113883
C. arabica	18.08	150	9	2	1.58113883
C. arabica	18.08	150	10	3	1.870828693
C. arabica	18.08	150	11	2	1.58113883
C. arabica	18.08	150	12	2	1.58113883
C. arabica	18.08	150	13	2	1.58113883

C. arabica	18.08	150	14	3	1.870828693
C. arabica	18.08	150	15	3	1.870828693
C. arabica	18.08	180	1	1	1.224744871
C. arabica	18.08	180	2	1	1.224744871
C. arabica	18.08	180	3	3	1.870828693
C. arabica	18.08	180	4	2	1.58113883
C. arabica	18.08	180	5	3	1.870828693
C. arabica	18.08	180	6	3	1.870828693
C. arabica	18.08	180	7	3	1.870828693
C. arabica	18.08	180	8	3	1.870828693
C. arabica	18.08	180	9	2	1.58113883
C. arabica	18.08	180	10	3	1.870828693
C. arabica	18.08	180	11	2	1.58113883
C. arabica	18.08	180	12	2	1.58113883
C. arabica	18.08	180	13	2	1.58113883
C. arabica	18.08	180	14	3	1.870828693
C. arabica	18.08	180	15	3	1.870828693
C. arabica	18.08	210	1	3	1.870828693
C. arabica	18.08	210	2	3	1.870828693
C. arabica	18.08	210	3	2	1.58113883
C. arabica	18.08	210	4	2	1.58113883
C. arabica	18.08	210	5	2	1.58113883
C. arabica	18.08	210	6	3	1.870828693
C. arabica	18.08	210	7	5	2.34520788
C. arabica	18.08	210	8	4	2.121320344
C. arabica	18.08	210	9	3	1.870828693
C. arabica	18.08	210	10	3	1.870828693
C. arabica	18.08	210	11	3	1.870828693
C. arabica	18.08	210	12	4	2.121320344
C. arabica	18.08	210	13	4	2.121320344
C. arabica	18.08	210	14	4	2.121320344
C. arabica	18.08	210	15	3	1.870828693
C. arabica	18.08	240	1	4	2.121320344
C. arabica	18.08	240	2	4	2.121320344
C. arabica	18.08	240	3	3	1.870828693
C. arabica	18.08	240	4	3	1.870828693
C. arabica	18.08	240	5	4	2.121320344
C. arabica	18.08	240	6	3	1.870828693
C. arabica	18.08	240	7	5	2.34520788
C. arabica	18.08	240	8	5	2.34520788
C. arabica	18.08	240	9	3	1.870828693
C. arabica	18.08	240	10	4	2.121320344
C. arabica	18.08	240	11	5	2.34520788
C. arabica	18.08	240	12	5	2.34520788
C. arabica	18.08	240	13	3	1.870828693
C. arabica	18.08	240	14	3	1.870828693
C. arabica	18.08	240	15	3	1.870828693
C. arabica	36.24	30	1	0	0.707106781
C. arabica	36.24	30	2	0	0.707106781

C. arabica	36.24	30	3	0	0.707106781
C. arabica	36.24	30	4	0	0.707106781
C. arabica	36.24	30	5	0	0.707106781
C. arabica	36.24	30	6	0	0.707106781
C. arabica	36.24	30	7	0	0.707106781
C. arabica	36.24	30	8	0	0.707106781
C. arabica	36.24	30	9	0	0.707106781
C. arabica	36.24	30	10	0	0.707106781
C. arabica	36.24	30	11	0	0.707106781
C. arabica	36.24	30	12	0	0.707106781
C. arabica	36.24	30	13	0	0.707106781
C. arabica	36.24	30	14	0	0.707106781
C. arabica	36.24	30	15	0	0.707106781
C. arabica	36.24	60	1	0	0.707106781
C. arabica	36.24	60	2	0	0.707106781
C. arabica	36.24	60	3	0	0.707106781
C. arabica	36.24	60	4	0	0.707106781
C. arabica	36.24	60	5	0	0.707106781
C. arabica	36.24	60	6	0	0.707106781
C. arabica	36.24	60	7	0	0.707106781
C. arabica	36.24	60	8	0	0.707106781
C. arabica	36.24	60	9	0	0.707106781
C. arabica	36.24	60	10	0	0.707106781
C. arabica	36.24	60	11	0	0.707106781
C. arabica	36.24	60	12	0	0.707106781
C. arabica	36.24	60	13	0	0.707106781
C. arabica	36.24	60	14	0	0.707106781
C. arabica	36.24	60	15	0	0.707106781
C. arabica	36.24	90	1	0	0.707106781
C. arabica	36.24	90	2	0	0.707106781
C. arabica	36.24	90	3	0	0.707106781
C. arabica	36.24	90	4	0	0.707106781
C. arabica	36.24	90	5	0	0.707106781
C. arabica	36.24	90	6	1	1.224744871
C. arabica	36.24	90	7	1	1.224744871
C. arabica	36.24	90	8	1	1.224744871
C. arabica	36.24	90	9	0	0.707106781
C. arabica	36.24	90	10	0	0.707106781
C. arabica	36.24	90	11	0	0.707106781
C. arabica	36.24	90	12	0	0.707106781
C. arabica	36.24	90	13	0	0.707106781
C. arabica	36.24	90	14	0	0.707106781
C. arabica	36.24	90	15	0	0.707106781
C. arabica	36.24	120	1	0	0.707106781
C. arabica	36.24	120	2	0	0.707106781
C. arabica	36.24	120	3	0	0.707106781
C. arabica	36.24	120	4	0	0.707106781
C. arabica	36.24	120	5	1	1.224744871
C. arabica	36.24	120	6	1	1.224744871

C. arabica	36.24	120	7	1	1.224744871
C. arabica	36.24	120	8	1	1.224744871
C. arabica	36.24	120	9	1	1.224744871
C. arabica	36.24	120	10	0	0.707106781
C. arabica	36.24	120	11	0	0.707106781
C. arabica	36.24	120	12	0	0.707106781
C. arabica	36.24	120	13	0	0.707106781
C. arabica	36.24	120	14	0	0.707106781
C. arabica	36.24	120	15	0	0.707106781
C. arabica	36.24	150	1	0	0.707106781
C. arabica	36.24	150	2	0	0.707106781
C. arabica	36.24	150	3	1	1.224744871
C. arabica	36.24	150	4	1	1.224744871
C. arabica	36.24	150	5	1	1.224744871
C. arabica	36.24	150	6	1	1.224744871
C. arabica	36.24	150	7	2	1.58113883
C. arabica	36.24	150	8	2	1.58113883
C. arabica	36.24	150	9	2	1.58113883
C. arabica	36.24	150	10	1	1.224744871
C. arabica	36.24	150	11	1	1.224744871
C. arabica	36.24	150	12	1	1.224744871
C. arabica	36.24	150	13	1	1.224744871
C. arabica	36.24	150	14	1	1.224744871
C. arabica	36.24	150	15	1	1.224744871
C. arabica	36.24	180	1	0	0.707106781
C. arabica	36.24	180	2	1	1.224744871
C. arabica	36.24	180	3	1	1.224744871
C. arabica	36.24	180	4	2	1.58113883
C. arabica	36.24	180	5	2	1.58113883
C. arabica	36.24	180	6	2	1.58113883
C. arabica	36.24	180	7	2	1.58113883
C. arabica	36.24	180	8	2	1.58113883
C. arabica	36.24	180	9	2	1.58113883
C. arabica	36.24	180	10	3	1.870828693
C. arabica	36.24	180	11	1	1.224744871
C. arabica	36.24	180	12	1	1.224744871
C. arabica	36.24	180	13	1	1.224744871
C. arabica	36.24	180	14	1	1.224744871
C. arabica	36.24	180	15	1	1.224744871
C. arabica	36.24	210	1	1	1.224744871
C. arabica	36.24	210	2	1	1.224744871
C. arabica	36.24	210	3	2	1.58113883
C. arabica	36.24	210	4	2	1.58113883
C. arabica	36.24	210	5	2	1.58113883
C. arabica	36.24	210	6	3	1.870828693
C. arabica	36.24	210	7	3	1.870828693
C. arabica	36.24	210	8	3	1.870828693
C. arabica	36.24	210	9	3	1.870828693
C. arabica	36.24	210	10	3	1.870828693

C. arabica	36.24	210	11	3	1.870828693
C. arabica	36.24	210	12	3	1.870828693
C. arabica	36.24	210	13	2	1.58113883
C. arabica	36.24	210	14	2	1.58113883
C. arabica	36.24	210	15	2	1.58113883
C. arabica	36.24	240	1	3	1.870828693
C. arabica	36.24	240	2	2	1.58113883
C. arabica	36.24	240	3	2	1.58113883
C. arabica	36.24	240	4	3	1.870828693
C. arabica	36.24	240	5	2	1.58113883
C. arabica	36.24	240	6	2	1.58113883
C. arabica	36.24	240	7	5	2.34520788
C. arabica	36.24	240	8	4	2.121320344
C. arabica	36.24	240	9	4	2.121320344
C. arabica	36.24	240	10	6	2.549509757
C. arabica	36.24	240	11	4	2.121320344
C. arabica	36.24	240	12	4	2.121320344
C. arabica	36.24	240	13	5	2.34520788
C. arabica	36.24	240	14	3	1.870828693
C. arabica	36.24	240	15	3	1.870828693
C. arabica	54.36	30	1	0	0.707106781
C. arabica	54.36	30	2	0	0.707106781
C. arabica	54.36	30	3	0	0.707106781
C. arabica	54.36	30	4	0	0.707106781
C. arabica	54.36	30	5	0	0.707106781
C. arabica	54.36	30	6	0	0.707106781
C. arabica	54.36	30	7	0	0.707106781
C. arabica	54.36	30	8	0	0.707106781
C. arabica	54.36	30	9	0	0.707106781
C. arabica	54.36	30	10	0	0.707106781
C. arabica	54.36	30	11	0	0.707106781
C. arabica	54.36	30	12	0	0.707106781
C. arabica	54.36	30	13	0	0.707106781
C. arabica	54.36	30	14	0	0.707106781
C. arabica	54.36	30	15	0	0.707106781
C. arabica	54.36	60	1	0	0.707106781
C. arabica	54.36	60	2	0	0.707106781
C. arabica	54.36	60	3	0	0.707106781
C. arabica	54.36	60	4	0	0.707106781
C. arabica	54.36	60	5	0	0.707106781
C. arabica	54.36	60	6	0	0.707106781
C. arabica	54.36	60	7	0	0.707106781
C. arabica	54.36	60	8	0	0.707106781
C. arabica	54.36	60	9	0	0.707106781
C. arabica	54.36	60	10	0	0.707106781
C. arabica	54.36	60	11	0	0.707106781
C. arabica	54.36	60	12	0	0.707106781
C. arabica	54.36	60	13	0	0.707106781
C. arabica	54.36	60	14	0	0.707106781

C. arabica	54.36	60	15	1	1.224744871
C. arabica	54.36	90	1	1	1.224744871
C. arabica	54.36	90	2	0	0.707106781
C. arabica	54.36	90	3	0	0.707106781
C. arabica	54.36	90	4	1	1.224744871
C. arabica	54.36	90	5	1	1.224744871
C. arabica	54.36	90	6	1	1.224744871
C. arabica	54.36	90	7	1	1.224744871
C. arabica	54.36	90	8	0	0.707106781
C. arabica	54.36	90	9	0	0.707106781
C. arabica	54.36	90	10	0	0.707106781
C. arabica	54.36	90	11	1	1.224744871
C. arabica	54.36	90	12	1	1.224744871
C. arabica	54.36	90	13	0	0.707106781
C. arabica	54.36	90	14	0	0.707106781
C. arabica	54.36	90	15	1	1.224744871
C. arabica	54.36	120	1	4	2.121320344
C. arabica	54.36	120	2	3	1.870828693
C. arabica	54.36	120	3	3	1.870828693
C. arabica	54.36	120	4	3	1.870828693
C. arabica	54.36	120	5	4	2.121320344
C. arabica	54.36	120	6	4	2.121320344
C. arabica	54.36	120	7	4	2.121320344
C. arabica	54.36	120	8	3	1.870828693
C. arabica	54.36	120	9	3	1.870828693
C. arabica	54.36	120	10	3	1.870828693
C. arabica	54.36	120	11	4	2.121320344
C. arabica	54.36	120	12	3	1.870828693
C. arabica	54.36	120	13	3	1.870828693
C. arabica	54.36	120	14	3	1.870828693
C. arabica	54.36	120	15	3	1.870828693
C. arabica	54.36	150	1	6	2.549509757
C. arabica	54.36	150	2	8	2.915475947
C. arabica	54.36	150	3	6	2.549509757
C. arabica	54.36	150	4	7	2.738612788
C. arabica	54.36	150	5	7	2.738612788
C. arabica	54.36	150	6	8	2.915475947
C. arabica	54.36	150	7	6	2.549509757
C. arabica	54.36	150	8	6	2.549509757
C. arabica	54.36	150	9	6	2.549509757
C. arabica	54.36	150	10	7	2.738612788
C. arabica	54.36	150	11	7	2.738612788
C. arabica	54.36	150	12	7	2.738612788
C. arabica	54.36	150	13	7	2.738612788
C. arabica	54.36	150	14	8	2.915475947
C. arabica	54.36	150	15	8	2.915475947
C. arabica	54.36	180	1	8	2.915475947
C. arabica	54.36	180	2	9	3.082207001
C. arabica	54.36	180	3	8	2.915475947

C. arabica	54.36	180	4	9	3.082207001
C. arabica	54.36	180	5	8	2.915475947
C. arabica	54.36	180	6	9	3.082207001
C. arabica	54.36	180	7	8	2.915475947
C. arabica	54.36	180	8	10	3.240370349
C. arabica	54.36	180	9	10	3.240370349
C. arabica	54.36	180	10	8	2.915475947
C. arabica	54.36	180	11	10	3.240370349
C. arabica	54.36	180	12	9	3.082207001
C. arabica	54.36	180	13	10	3.240370349
C. arabica	54.36	180	14	10	3.240370349
C. arabica	54.36	180	15	10	3.240370349
C. arabica	54.36	210	1	11	3.391164992
C. arabica	54.36	210	2	13	3.674234614
C. arabica	54.36	210	3	10	3.240370349
C. arabica	54.36	210	4	14	3.807886553
C. arabica	54.36	210	5	10	3.240370349
C. arabica	54.36	210	6	11	3.391164992
C. arabica	54.36	210	7	14	3.807886553
C. arabica	54.36	210	8	11	3.391164992
C. arabica	54.36	210	9	12	3.535533906
C. arabica	54.36	210	10	10	3.240370349
C. arabica	54.36	210	11	13	3.674234614
C. arabica	54.36	210	12	12	3.535533906
C. arabica	54.36	210	13	15	3.937003937
C. arabica	54.36	210	14	15	3.937003937
C. arabica	54.36	210	15	15	3.937003937
C. arabica	54.36	240	1	11	3.391164992
C. arabica	54.36	240	2	13	3.674234614
C. arabica	54.36	240	3	11	3.391164992
C. arabica	54.36	240	4	13	3.674234614
C. arabica	54.36	240	5	14	3.807886553
C. arabica	54.36	240	6	11	3.391164992
C. arabica	54.36	240	7	15	3.937003937
C. arabica	54.36	240	8	12	3.535533906
C. arabica	54.36	240	9	11	3.391164992
C. arabica	54.36	240	10	12	3.535533906
C. arabica	54.36	240	11	15	3.937003937
C. arabica	54.36	240	12	12	3.535533906
C. arabica	54.36	240	13	15	3.937003937
C. arabica	54.36	240	14	15	3.937003937
C. arabica	54.36	240	15	15	3.937003937
C. canephora	9.06	30	1	0	0.707106781
C. canephora	9.06	30	2	0	0.707106781
C. canephora	9.06	30	3	0	0.707106781
C. canephora	9.06	30	4	0	0.707106781
C. canephora	9.06	30	5	0	0.707106781
C. canephora	9.06	30	6	0	0.707106781
C. canephora	9.06	30	7	0	0.707106781

C. canephora	9.06	30	8	0	0.707106781
C. canephora	9.06	60	1	0	0.707106781
C. canephora	9.06	60	2	0	0.707106781
C. canephora	9.06	60	3	0	0.707106781
C. canephora	9.06	60	4	0	0.707106781
C. canephora	9.06	60	5	0	0.707106781
C. canephora	9.06	60	6	0	0.707106781
C. canephora	9.06	60	7	0	0.707106781
C. canephora	9.06	60	8	0	0.707106781
C. canephora	9.06	90	1	0	0.707106781
C. canephora	9.06	90	2	0	0.707106781
C. canephora	9.06	90	3	0	0.707106781
C. canephora	9.06	90	4	0	0.707106781
C. canephora	9.06	90	5	0	0.707106781
C. canephora	9.06	90	6	0	0.707106781
C. canephora	9.06	90	7	0	0.707106781
C. canephora	9.06	90	8	0	0.707106781
C. canephora	9.06	120	1	0	0.707106781
C. canephora	9.06	120	2	0	0.707106781
C. canephora	9.06	120	3	0	0.707106781
C. canephora	9.06	120	4	0	0.707106781
C. canephora	9.06	120	5	0	0.707106781
C. canephora	9.06	120	6	0	0.707106781
C. canephora	9.06	120	7	0	0.707106781
C. canephora	9.06	120	8	0	0.707106781
C. canephora	9.06	150	1	0	0.707106781
C. canephora	9.06	150	2	0	0.707106781
C. canephora	9.06	150	3	0	0.707106781
C. canephora	9.06	150	4	0	0.707106781
C. canephora	9.06	150	5	0	0.707106781
C. canephora	9.06	150	6	0	0.707106781
C. canephora	9.06	150	7	0	0.707106781
C. canephora	9.06	150	8	0	0.707106781
C. canephora	9.06	180	1	1	1.224744871
C. canephora	9.06	180	2	0	0.707106781
C. canephora	9.06	180	3	0	0.707106781
C. canephora	9.06	180	4	0	0.707106781
C. canephora	9.06	180	5	0	0.707106781
C. canephora	9.06	180	6	0	0.707106781
C. canephora	9.06	180	7	0	0.707106781
C. canephora	9.06	180	8	0	0.707106781
C. canephora	9.06	210	1	1	1.224744871
C. canephora	9.06	210	2	1	1.224744871
C. canephora	9.06	210	3	1	1.224744871
C. canephora	9.06	210	4	1	1.224744871
C. canephora	9.06	210	5	0	0.707106781
C. canephora	9.06	210	6	0	0.707106781
C. canephora	9.06	210	7	0	0.707106781
C. canephora	9.06	210	8	0	0.707106781

C. canephora	9.06	240	1	2	1.58113883
C. canephora	9.06	240	2	1	1.224744871
C. canephora	9.06	240	3	1	1.224744871
C. canephora	9.06	240	4	1	1.224744871
C. canephora	9.06	240	5	1	1.224744871
C. canephora	9.06	240	6	0	0.707106781
C. canephora	9.06	240	7	0	0.707106781
C. canephora	9.06	240	8	0	0.707106781
C. canephora	18.08	30	1	0	0.707106781
C. canephora	18.08	30	2	0	0.707106781
C. canephora	18.08	30	3	0	0.707106781
C. canephora	18.08	30	4	0	0.707106781
C. canephora	18.08	30	5	0	0.707106781
C. canephora	18.08	30	6	0	0.707106781
C. canephora	18.08	30	7	0	0.707106781
C. canephora	18.08	30	8	0	0.707106781
C. canephora	18.08	60	1	0	0.707106781
C. canephora	18.08	60	2	0	0.707106781
C. canephora	18.08	60	3	0	0.707106781
C. canephora	18.08	60	4	0	0.707106781
C. canephora	18.08	60	5	0	0.707106781
C. canephora	18.08	60	6	0	0.707106781
C. canephora	18.08	60	7	0	0.707106781
C. canephora	18.08	60	8	0	0.707106781
C. canephora	18.08	90	1	0	0.707106781
C. canephora	18.08	90	2	0	0.707106781
C. canephora	18.08	90	3	0	0.707106781
C. canephora	18.08	90	4	0	0.707106781
C. canephora	18.08	90	5	0	0.707106781
C. canephora	18.08	90	6	0	0.707106781
C. canephora	18.08	90	7	0	0.707106781
C. canephora	18.08	90	8	0	0.707106781
C. canephora	18.08	120	1	0	0.707106781
C. canephora	18.08	120	2	0	0.707106781
C. canephora	18.08	120	3	0	0.707106781
C. canephora	18.08	120	4	0	0.707106781
C. canephora	18.08	120	5	0	0.707106781
C. canephora	18.08	120	6	1	1.224744871
C. canephora	18.08	120	7	1	1.224744871
C. canephora	18.08	120	8	0	0.707106781
C. canephora	18.08	150	1	0	0.707106781
C. canephora	18.08	150	2	0	0.707106781
C. canephora	18.08	150	3	1	1.224744871
C. canephora	18.08	150	4	1	1.224744871
C. canephora	18.08	150	5	2	1.58113883
C. canephora	18.08	150	6	1	1.224744871
C. canephora	18.08	150	7	1	1.224744871
C. canephora	18.08	150	8	1	1.224744871
C. canephora	18.08	180	1	0	0.707106781

C. canephora	18.08	180	2	1	1.224744871
C. canephora	18.08	180	3	1	1.224744871
C. canephora	18.08	180	4	2	1.58113883
C. canephora	18.08	180	5	2	1.58113883
C. canephora	18.08	180	6	3	1.870828693
C. canephora	18.08	180	7	3	1.870828693
C. canephora	18.08	180	8	3	1.870828693
C. canephora	18.08	210	1	3	1.870828693
C. canephora	18.08	210	2	3	1.870828693
C. canephora	18.08	210	3	5	2.34520788
C. canephora	18.08	210	4	4	2.121320344
C. canephora	18.08	210	5	3	1.870828693
C. canephora	18.08	210	6	5	2.34520788
C. canephora	18.08	210	7	3	1.870828693
C. canephora	18.08	210	8	4	2.121320344
C. canephora	18.08	240	1	4	2.121320344
C. canephora	18.08	240	2	4	2.121320344
C. canephora	18.08	240	3	3	1.870828693
C. canephora	18.08	240	4	5	2.34520788
C. canephora	18.08	240	5	5	2.34520788
C. canephora	18.08	240	6	5	2.34520788
C. canephora	18.08	240	7	3	1.870828693
C. canephora	18.08	240	8	3	1.870828693
C. canephora	36.24	30	1	0	0.707106781
C. canephora	36.24	30	2	0	0.707106781
C. canephora	36.24	30	3	0	0.707106781
C. canephora	36.24	30	4	0	0.707106781
C. canephora	36.24	30	5	0	0.707106781
C. canephora	36.24	30	6	0	0.707106781
C. canephora	36.24	30	7	0	0.707106781
C. canephora	36.24	30	8	0	0.707106781
C. canephora	36.24	60	1	0	0.707106781
C. canephora	36.24	60	2	0	0.707106781
C. canephora	36.24	60	3	0	0.707106781
C. canephora	36.24	60	4	0	0.707106781
C. canephora	36.24	60	5	0	0.707106781
C. canephora	36.24	60	6	0	0.707106781
C. canephora	36.24	60	7	0	0.707106781
C. canephora	36.24	60	8	0	0.707106781
C. canephora	36.24	90	1	0	0.707106781
C. canephora	36.24	90	2	0	0.707106781
C. canephora	36.24	90	3	0	0.707106781
C. canephora	36.24	90	4	0	0.707106781
C. canephora	36.24	90	5	0	0.707106781
C. canephora	36.24	90	6	0	0.707106781
C. canephora	36.24	90	7	0	0.707106781
C. canephora	36.24	90	8	0	0.707106781
C. canephora	36.24	120	1	0	0.707106781
C. canephora	36.24	120	2	0	0.707106781

C. canephora	36.24	120	3	0	0.707106781
C. canephora	36.24	120	4	0	0.707106781
C. canephora	36.24	120	5	0	0.707106781
C. canephora	36.24	120	6	0	0.707106781
C. canephora	36.24	120	7	0	0.707106781
C. canephora	36.24	120	8	0	0.707106781
C. canephora	36.24	150	1	0	0.707106781
C. canephora	36.24	150	2	0	0.707106781
C. canephora	36.24	150	3	0	0.707106781
C. canephora	36.24	150	4	0	0.707106781
C. canephora	36.24	150	5	0	0.707106781
C. canephora	36.24	150	6	0	0.707106781
C. canephora	36.24	150	7	0	0.707106781
C. canephora	36.24	150	8	0	0.707106781
C. canephora	36.24	180	1	1	1.224744871
C. canephora	36.24	180	2	1	1.224744871
C. canephora	36.24	180	3	1	1.224744871
C. canephora	36.24	180	4	0	0.707106781
C. canephora	36.24	180	5	1	1.224744871
C. canephora	36.24	180	6	2	1.58113883
C. canephora	36.24	180	7	1	1.224744871
C. canephora	36.24	180	8	2	1.58113883
C. canephora	36.24	210	1	3	1.870828693
C. canephora	36.24	210	2	1	1.224744871
C. canephora	36.24	210	3	2	1.58113883
C. canephora	36.24	210	4	2	1.58113883
C. canephora	36.24	210	5	3	1.870828693
C. canephora	36.24	210	6	3	1.870828693
C. canephora	36.24	210	7	3	1.870828693
C. canephora	36.24	210	8	3	1.870828693
C. canephora	36.24	240	1	5	2.34520788
C. canephora	36.24	240	2	5	2.34520788
C. canephora	36.24	240	3	4	2.121320344
C. canephora	36.24	240	4	4	2.121320344
C. canephora	36.24	240	5	3	1.870828693
C. canephora	36.24	240	6	3	1.870828693
C. canephora	36.24	240	7	4	2.121320344
C. canephora	36.24	240	8	4	2.121320344
C. canephora	54.36	30	1	0	0.707106781
C. canephora	54.36	30	2	0	0.707106781
C. canephora	54.36	30	3	0	0.707106781
C. canephora	54.36	30	4	0	0.707106781
C. canephora	54.36	30	5	0	0.707106781
C. canephora	54.36	30	6	0	0.707106781
C. canephora	54.36	30	7	0	0.707106781
C. canephora	54.36	30	8	0	0.707106781
C. canephora	54.36	30	9	0	0.707106781
C. canephora	54.36	30	10	0	0.707106781
C. canephora	54.36	30	11	0	0.707106781

C. canephora	54.36	30	12	0	0.707106781
C. canephora	54.36	30	13	0	0.707106781
C. canephora	54.36	30	14	0	0.707106781
C. canephora	54.36	30	15	0	0.707106781
C. canephora	54.36	30	16	0	0.707106781
C. canephora	54.36	30	17	0	0.707106781
C. canephora	54.36	30	18	0	0.707106781
C. canephora	54.36	30	19	0	0.707106781
C. canephora	54.36	30	20	0	0.707106781
C. canephora	54.36	60	1	0	0.707106781
C. canephora	54.36	60	2	0	0.707106781
C. canephora	54.36	60	3	0	0.707106781
C. canephora	54.36	60	4	0	0.707106781
C. canephora	54.36	60	5	0	0.707106781
C. canephora	54.36	60	6	0	0.707106781
C. canephora	54.36	60	7	0	0.707106781
C. canephora	54.36	60	8	0	0.707106781
C. canephora	54.36	60	9	0	0.707106781
C. canephora	54.36	60	10	0	0.707106781
C. canephora	54.36	60	11	0	0.707106781
C. canephora	54.36	60	12	0	0.707106781
C. canephora	54.36	60	13	0	0.707106781
C. canephora	54.36	60	14	0	0.707106781
C. canephora	54.36	60	15	0	0.707106781
C. canephora	54.36	60	16	0	0.707106781
C. canephora	54.36	60	17	0	0.707106781
C. canephora	54.36	60	18	0	0.707106781
C. canephora	54.36	60	19	0	0.707106781
C. canephora	54.36	60	20	0	0.707106781
C. canephora	54.36	90	1	0	0.707106781
C. canephora	54.36	90	2	0	0.707106781
C. canephora	54.36	90	3	0	0.707106781
C. canephora	54.36	90	4	0	0.707106781
C. canephora	54.36	90	5	0	0.707106781
C. canephora	54.36	90	6	0	0.707106781
C. canephora	54.36	90	7	0	0.707106781
C. canephora	54.36	90	8	0	0.707106781
C. canephora	54.36	90	9	0	0.707106781
C. canephora	54.36	90	10	0	0.707106781
C. canephora	54.36	90	11	0	0.707106781
C. canephora	54.36	90	12	0	0.707106781
C. canephora	54.36	90	13	1	1.224744871
C. canephora	54.36	90	14	1	1.224744871
C. canephora	54.36	90	15	0	0.707106781
C. canephora	54.36	90	16	0	0.707106781
C. canephora	54.36	90	17	0	0.707106781
C. canephora	54.36	90	18	0	0.707106781
C. canephora	54.36	90	19	1	1.224744871
C. canephora	54.36	90	20	1	1.224744871

C. canephora	54.36	120	1	1	1.224744871
C. canephora	54.36	120	2	1	1.224744871
C. canephora	54.36	120	3	1	1.224744871
C. canephora	54.36	120	4	1	1.224744871
C. canephora	54.36	120	5	0	0.707106781
C. canephora	54.36	120	6	0	0.707106781
C. canephora	54.36	120	7	0	0.707106781
C. canephora	54.36	120	8	0	0.707106781
C. canephora	54.36	120	9	1	1.224744871
C. canephora	54.36	120	10	1	1.224744871
C. canephora	54.36	120	11	2	1.58113883
C. canephora	54.36	120	12	1	1.224744871
C. canephora	54.36	120	13	2	1.58113883
C. canephora	54.36	120	14	2	1.58113883
C. canephora	54.36	120	15	2	1.58113883
C. canephora	54.36	120	16	1	1.224744871
C. canephora	54.36	120	17	2	1.58113883
C. canephora	54.36	120	18	1	1.224744871
C. canephora	54.36	120	19	1	1.224744871
C. canephora	54.36	120	20	2	1.58113883
C. canephora	54.36	150	1	5	2.34520788
C. canephora	54.36	150	2	5	2.34520788
C. canephora	54.36	150	3	4	2.121320344
C. canephora	54.36	150	4	4	2.121320344
C. canephora	54.36	150	5	3	1.870828693
C. canephora	54.36	150	6	3	1.870828693
C. canephora	54.36	150	7	3	1.870828693
C. canephora	54.36	150	8	3	1.870828693
C. canephora	54.36	150	9	0	0.707106781
C. canephora	54.36	150	10	4	2.121320344
C. canephora	54.36	150	11	4	2.121320344
C. canephora	54.36	150	12	4	2.121320344
C. canephora	54.36	150	13	4	2.121320344
C. canephora	54.36	150	14	4	2.121320344
C. canephora	54.36	150	15	4	2.121320344
C. canephora	54.36	150	16	4	2.121320344
C. canephora	54.36	150	17	5	2.34520788
C. canephora	54.36	150	18	4	2.121320344
C. canephora	54.36	150	19	4	2.121320344
C. canephora	54.36	150	20	4	2.121320344
C. canephora	54.36	180	1	6	2.549509757
C. canephora	54.36	180	2	8	2.915475947
C. canephora	54.36	180	3	8	2.915475947
C. canephora	54.36	180	4	8	2.915475947
C. canephora	54.36	180	5	6	2.549509757
C. canephora	54.36	180	6	9	3.082207001
C. canephora	54.36	180	7	8	2.915475947
C. canephora	54.36	180	8	8	2.915475947
C. canephora	54.36	180	9	8	2.915475947

C. canephora	54.36	180	10	10	3.240370349
C. canephora	54.36	180	11	8	2.915475947
C. canephora	54.36	180	12	9	3.082207001
C. canephora	54.36	180	13	9	3.082207001
C. canephora	54.36	180	14	9	3.082207001
C. canephora	54.36	180	15	8	2.915475947
C. canephora	54.36	180	16	8	2.915475947
C. canephora	54.36	180	17	9	3.082207001
C. canephora	54.36	180	18	8	2.915475947
C. canephora	54.36	180	19	8	2.915475947
C. canephora	54.36	180	20	8	2.915475947
C. canephora	54.36	210	1	8	2.915475947
C. canephora	54.36	210	2	11	3.391164992
C. canephora	54.36	210	3	8	2.915475947
C. canephora	54.36	210	4	10	3.240370349
C. canephora	54.36	210	5	11	3.391164992
C. canephora	54.36	210	6	10	3.240370349
C. canephora	54.36	210	7	8	2.915475947
C. canephora	54.36	210	8	9	3.082207001
C. canephora	54.36	210	9	9	3.082207001
C. canephora	54.36	210	10	10	3.240370349
C. canephora	54.36	210	11	11	3.391164992
C. canephora	54.36	210	12	9	3.082207001
C. canephora	54.36	210	13	13	3.674234614
C. canephora	54.36	210	14	10	3.240370349
C. canephora	54.36	210	15	13	3.674234614
C. canephora	54.36	210	16	12	3.535533906
C. canephora	54.36	210	17	10	3.240370349
C. canephora	54.36	210	18	12	3.535533906
C. canephora	54.36	210	19	12	3.535533906
C. canephora	54.36	210	20	13	3.674234614
C. canephora	54.36	240	1	10	3.240370349
C. canephora	54.36	240	2	13	3.674234614
C. canephora	54.36	240	3	10	3.240370349
C. canephora	54.36	240	4	13	3.674234614
C. canephora	54.36	240	5	11	3.391164992
C. canephora	54.36	240	6	11	3.391164992
C. canephora	54.36	240	7	10	3.240370349
C. canephora	54.36	240	8	10	3.240370349
C. canephora	54.36	240	9	10	3.240370349
C. canephora	54.36	240	10	10	3.240370349
C. canephora	54.36	240	11	10	3.240370349
C. canephora	54.36	240	12	10	3.240370349
C. canephora	54.36	240	13	10	3.240370349
C. canephora	54.36	240	14	13	3.674234614
C. canephora	54.36	240	15	13	3.674234614
C. canephora	54.36	240	16	11	3.391164992
C. canephora	54.36	240	17	13	3.674234614
C. canephora	54.36	240	18	13	3.674234614

C. canephora	54.36	240	19	15	3.937003937
C. canephora	54.36	240	20	14	3.807886553
