# Supplementary material for: Embryonic abnormalities and genotoxicity induced by 2,4-dichlorophenoxyacetic acid during indirect somatic embryogenesis in Coffea
Source: Sci Rep. 2023 Jun 15;13:9689. doi: 10.1038/s41598-023-36879-7 (PMC10272143; doi:10.1038/s41598-023-36879-7)
Supplement: Supplementary file 6 — Supplementary Table 3. [file 41598_2023_36879_MOESM6_ESM.pdf]

SI Table 3. All data generated and analysed about the abnormal somatic embryo (ASE) regenerated from *C. arabica* and *C. canephora* friable callus.

| *Coffea* species | 2,4-D (µM) | Days | Repetition | ASE | Adjusted data |
|---|---|---|---|---|---|
| *C. arabica* | 9.06 | 30 | 1 | 0 | 0.707106781 |
| *C. arabica* | 9.06 | 30 | 2 | 0 | 0.707106781 |
| *C. arabica* | 9.06 | 30 | 3 | 0 | 0.707106781 |
| *C. arabica* | 9.06 | 30 | 4 | 0 | 0.707106781 |
| *C. arabica* | 9.06 | 30 | 5 | 0 | 0.707106781 |
| *C. arabica* | 9.06 | 30 | 6 | 0 | 0.707106781 |
| *C. arabica* | 9.06 | 30 | 7 | 0 | 0.707106781 |
| *C. arabica* | 9.06 | 30 | 8 | 0 | 0.707106781 |
| *C. arabica* | 9.06 | 30 | 9 | 0 | 0.707106781 |
| *C. arabica* | 9.06 | 30 | 10 | 0 | 0.707106781 |
| *C. arabica* | 9.06 | 30 | 11 | 0 | 0.707106781 |
| *C. arabica* | 9.06 | 30 | 12 | 0 | 0.707106781 |
| *C. arabica* | 9.06 | 30 | 13 | 0 | 0.707106781 |
| *C. arabica* | 9.06 | 30 | 14 | 0 | 0.707106781 |
| *C. arabica* | 9.06 | 30 | 15 | 0 | 0.707106781 |
| *C. arabica* | 9.06 | 60 | 1 | 0 | 0.707106781 |
| *C. arabica* | 9.06 | 60 | 2 | 0 | 0.707106781 |
| *C. arabica* | 9.06 | 60 | 3 | 0 | 0.707106781 |
| *C. arabica* | 9.06 | 60 | 4 | 0 | 0.707106781 |
| *C. arabica* | 9.06 | 60 | 5 | 0 | 0.707106781 |
| *C. arabica* | 9.06 | 60 | 6 | 0 | 0.707106781 |
| *C. arabica* | 9.06 | 60 | 7 | 0 | 0.707106781 |
| *C. arabica* | 9.06 | 60 | 8 | 0 | 0.707106781 |
| *C. arabica* | 9.06 | 60 | 9 | 0 | 0.707106781 |
| *C. arabica* | 9.06 | 60 | 10 | 0 | 0.707106781 |
| *C. arabica* | 9.06 | 60 | 11 | 0 | 0.707106781 |
| *C. arabica* | 9.06 | 60 | 12 | 0 | 0.707106781 |
| *C. arabica* | 9.06 | 60 | 13 | 0 | 0.707106781 |
| *C. arabica* | 9.06 | 60 | 14 | 0 | 0.707106781 |
| *C. arabica* | 9.06 | 60 | 15 | 0 | 0.707106781 |
| *C. arabica* | 9.06 | 90 | 1 | 0 | 0.707106781 |
| *C. arabica* | 9.06 | 90 | 2 | 0 | 0.707106781 |
| *C. arabica* | 9.06 | 90 | 3 | 0 | 0.707106781 |
| *C. arabica* | 9.06 | 90 | 4 | 0 | 0.707106781 |
| *C. arabica* | 9.06 | 90 | 5 | 0 | 0.707106781 |
| *C. arabica* | 9.06 | 90 | 6 | 0 | 0.707106781 |
| *C. arabica* | 9.06 | 90 | 7 | 1 | 1.224744871 |
| *C. arabica* | 9.06 | 90 | 8 | 1 | 1.224744871 |
| *C. arabica* | 9.06 | 90 | 9 | 0 | 0.707106781 |
| *C. arabica* | 9.06 | 90 | 10 | 1 | 1.224744871 |
| *C. arabica* | 9.06 | 90 | 11 | 0 | 0.707106781 |
| *C. arabica* | 9.06 | 90 | 12 | 0 | 0.707106781 |
| *C. arabica* | 9.06 | 90 | 13 | 0 | 0.707106781 |
| *C. arabica* | 9.06 | 90 | 14 | 0 | 0.707106781 |
| *C. arabica* | 9.06 | 90 | 15 | 0 | 0.707106781 |
| *C. arabica* | 9.06 | 120 | 1 | 0 | 0.707106781 |

| | | | | | |
|---|---|---|---|---|---|
| *C. arabica* | 9.06 | 120 | 2 | 0 | 0.707106781 |
| *C. arabica* | 9.06 | 120 | 3 | 1 | 1.224744871 |
| *C. arabica* | 9.06 | 120 | 4 | 1 | 1.224744871 |
| *C. arabica* | 9.06 | 120 | 5 | 1 | 1.224744871 |
| *C. arabica* | 9.06 | 120 | 6 | 1 | 1.224744871 |
| *C. arabica* | 9.06 | 120 | 7 | 1 | 1.224744871 |
| *C. arabica* | 9.06 | 120 | 8 | 1 | 1.224744871 |
| *C. arabica* | 9.06 | 120 | 9 | 1 | 1.224744871 |
| *C. arabica* | 9.06 | 120 | 10 | 2 | 1.58113883 |
| *C. arabica* | 9.06 | 120 | 11 | 1 | 1.224744871 |
| *C. arabica* | 9.06 | 120 | 12 | 1 | 1.224744871 |
| *C. arabica* | 9.06 | 120 | 13 | 1 | 1.224744871 |
| *C. arabica* | 9.06 | 120 | 14 | 1 | 1.224744871 |
| *C. arabica* | 9.06 | 120 | 15 | 1 | 1.224744871 |
| *C. arabica* | 9.06 | 150 | 1 | 0 | 0.707106781 |
| *C. arabica* | 9.06 | 150 | 2 | 4 | 2.121320344 |
| *C. arabica* | 9.06 | 150 | 3 | 4 | 2.121320344 |
| *C. arabica* | 9.06 | 150 | 4 | 4 | 2.121320344 |
| *C. arabica* | 9.06 | 150 | 5 | 4 | 2.121320344 |
| *C. arabica* | 9.06 | 150 | 6 | 4 | 2.121320344 |
| *C. arabica* | 9.06 | 150 | 7 | 4 | 2.121320344 |
| *C. arabica* | 9.06 | 150 | 8 | 5 | 2.34520788 |
| *C. arabica* | 9.06 | 150 | 9 | 4 | 2.121320344 |
| *C. arabica* | 9.06 | 150 | 10 | 4 | 2.121320344 |
| *C. arabica* | 9.06 | 150 | 11 | 5 | 2.34520788 |
| *C. arabica* | 9.06 | 150 | 12 | 5 | 2.34520788 |
| *C. arabica* | 9.06 | 150 | 13 | 4 | 2.121320344 |
| *C. arabica* | 9.06 | 150 | 14 | 4 | 2.121320344 |
| *C. arabica* | 9.06 | 150 | 15 | 4 | 2.121320344 |
| *C. arabica* | 9.06 | 180 | 1 | 7 | 2.738612788 |
| *C. arabica* | 9.06 | 180 | 2 | 8 | 2.915475947 |
| *C. arabica* | 9.06 | 180 | 3 | 9 | 3.082207001 |
| *C. arabica* | 9.06 | 180 | 4 | 8 | 2.915475947 |
| *C. arabica* | 9.06 | 180 | 5 | 8 | 2.915475947 |
| *C. arabica* | 9.06 | 180 | 6 | 9 | 3.082207001 |
| *C. arabica* | 9.06 | 180 | 7 | 8 | 2.915475947 |
| *C. arabica* | 9.06 | 180 | 8 | 9 | 3.082207001 |
| *C. arabica* | 9.06 | 180 | 9 | 9 | 3.082207001 |
| *C. arabica* | 9.06 | 180 | 10 | 9 | 3.082207001 |
| *C. arabica* | 9.06 | 180 | 11 | 8 | 2.915475947 |
| *C. arabica* | 9.06 | 180 | 12 | 8 | 2.915475947 |
| *C. arabica* | 9.06 | 180 | 13 | 8 | 2.915475947 |
| *C. arabica* | 9.06 | 180 | 14 | 8 | 2.915475947 |
| *C. arabica* | 9.06 | 180 | 15 | 9 | 3.082207001 |
| *C. arabica* | 9.06 | 210 | 1 | 15 | 3.937003937 |
| *C. arabica* | 9.06 | 210 | 2 | 16 | 4.062019202 |
| *C. arabica* | 9.06 | 210 | 3 | 16 | 4.062019202 |
| *C. arabica* | 9.06 | 210 | 4 | 16 | 4.062019202 |
| *C. arabica* | 9.06 | 210 | 5 | 18 | 4.301162634 |

| | | | | | |
|---|---|---|---|---|---|
| *C. arabica* | 9.06 | 210 | 6 | 17 | 4.183300133 |
| *C. arabica* | 9.06 | 210 | 7 | 19 | 4.415880433 |
| *C. arabica* | 9.06 | 210 | 8 | 17 | 4.183300133 |
| *C. arabica* | 9.06 | 210 | 9 | 16 | 4.062019202 |
| *C. arabica* | 9.06 | 210 | 10 | 17 | 4.183300133 |
| *C. arabica* | 9.06 | 210 | 11 | 18 | 4.301162634 |
| *C. arabica* | 9.06 | 210 | 12 | 20 | 4.527692569 |
| *C. arabica* | 9.06 | 210 | 13 | 20 | 4.527692569 |
| *C. arabica* | 9.06 | 210 | 14 | 15 | 3.937003937 |
| *C. arabica* | 9.06 | 210 | 15 | 17 | 4.183300133 |
| *C. arabica* | 9.06 | 240 | 1 | 15 | 3.937003937 |
| *C. arabica* | 9.06 | 240 | 2 | 16 | 4.062019202 |
| *C. arabica* | 9.06 | 240 | 3 | 17 | 4.183300133 |
| *C. arabica* | 9.06 | 240 | 4 | 17 | 4.183300133 |
| *C. arabica* | 9.06 | 240 | 5 | 18 | 4.301162634 |
| *C. arabica* | 9.06 | 240 | 6 | 20 | 4.527692569 |
| *C. arabica* | 9.06 | 240 | 7 | 19 | 4.415880433 |
| *C. arabica* | 9.06 | 240 | 8 | 18 | 4.301162634 |
| *C. arabica* | 9.06 | 240 | 9 | 17 | 4.183300133 |
| *C. arabica* | 9.06 | 240 | 10 | 18 | 4.301162634 |
| *C. arabica* | 9.06 | 240 | 11 | 18 | 4.301162634 |
| *C. arabica* | 9.06 | 240 | 12 | 20 | 4.527692569 |
| *C. arabica* | 9.06 | 240 | 13 | 20 | 4.527692569 |
| *C. arabica* | 9.06 | 240 | 14 | 17 | 4.183300133 |
| *C. arabica* | 9.06 | 240 | 15 | 18 | 4.301162634 |
| *C. arabica* | 18.08 | 30 | 1 | 0 | 0.707106781 |
| *C. arabica* | 18.08 | 30 | 2 | 0 | 0.707106781 |
| *C. arabica* | 18.08 | 30 | 3 | 0 | 0.707106781 |
| *C. arabica* | 18.08 | 30 | 4 | 0 | 0.707106781 |
| *C. arabica* | 18.08 | 30 | 5 | 0 | 0.707106781 |
| *C. arabica* | 18.08 | 30 | 6 | 0 | 0.707106781 |
| *C. arabica* | 18.08 | 30 | 7 | 0 | 0.707106781 |
| *C. arabica* | 18.08 | 30 | 8 | 0 | 0.707106781 |
| *C. arabica* | 18.08 | 30 | 9 | 0 | 0.707106781 |
| *C. arabica* | 18.08 | 30 | 10 | 0 | 0.707106781 |
| *C. arabica* | 18.08 | 30 | 11 | 0 | 0.707106781 |
| *C. arabica* | 18.08 | 30 | 12 | 0 | 0.707106781 |
| *C. arabica* | 18.08 | 30 | 13 | 0 | 0.707106781 |
| *C. arabica* | 18.08 | 30 | 14 | 0 | 0.707106781 |
| *C. arabica* | 18.08 | 30 | 15 | 0 | 0.707106781 |
| *C. arabica* | 18.08 | 60 | 1 | 0 | 0.707106781 |
| *C. arabica* | 18.08 | 60 | 2 | 0 | 0.707106781 |
| *C. arabica* | 18.08 | 60 | 3 | 0 | 0.707106781 |
| *C. arabica* | 18.08 | 60 | 4 | 0 | 0.707106781 |
| *C. arabica* | 18.08 | 60 | 5 | 0 | 0.707106781 |
| *C. arabica* | 18.08 | 60 | 6 | 0 | 0.707106781 |
| *C. arabica* | 18.08 | 60 | 7 | 0 | 0.707106781 |
| *C. arabica* | 18.08 | 60 | 8 | 0 | 0.707106781 |
| *C. arabica* | 18.08 | 60 | 9 | 0 | 0.707106781 |

| | | | | | |
|---|---|---|---|---|---|
| *C. arabica* | 18.08 | 60 | 10 | 0 | 0.707106781 |
| *C. arabica* | 18.08 | 60 | 11 | 0 | 0.707106781 |
| *C. arabica* | 18.08 | 60 | 12 | 0 | 0.707106781 |
| *C. arabica* | 18.08 | 60 | 13 | 0 | 0.707106781 |
| *C. arabica* | 18.08 | 60 | 14 | 0 | 0.707106781 |
| *C. arabica* | 18.08 | 60 | 15 | 0 | 0.707106781 |
| *C. arabica* | 18.08 | 90 | 1 | 0 | 0.707106781 |
| *C. arabica* | 18.08 | 90 | 2 | 0 | 0.707106781 |
| *C. arabica* | 18.08 | 90 | 3 | 1 | 1.224744871 |
| *C. arabica* | 18.08 | 90 | 4 | 1 | 1.224744871 |
| *C. arabica* | 18.08 | 90 | 5 | 1 | 1.224744871 |
| *C. arabica* | 18.08 | 90 | 6 | 1 | 1.224744871 |
| *C. arabica* | 18.08 | 90 | 7 | 1 | 1.224744871 |
| *C. arabica* | 18.08 | 90 | 8 | 1 | 1.224744871 |
| *C. arabica* | 18.08 | 90 | 9 | 1 | 1.224744871 |
| *C. arabica* | 18.08 | 90 | 10 | 1 | 1.224744871 |
| *C. arabica* | 18.08 | 90 | 11 | 0 | 0.707106781 |
| *C. arabica* | 18.08 | 90 | 12 | 0 | 0.707106781 |
| *C. arabica* | 18.08 | 90 | 13 | 1 | 1.224744871 |
| *C. arabica* | 18.08 | 90 | 14 | 2 | 1.58113883 |
| *C. arabica* | 18.08 | 90 | 15 | 2 | 1.58113883 |
| *C. arabica* | 18.08 | 120 | 1 | 1 | 1.224744871 |
| *C. arabica* | 18.08 | 120 | 2 | 1 | 1.224744871 |
| *C. arabica* | 18.08 | 120 | 3 | 2 | 1.58113883 |
| *C. arabica* | 18.08 | 120 | 4 | 1 | 1.224744871 |
| *C. arabica* | 18.08 | 120 | 5 | 1 | 1.224744871 |
| *C. arabica* | 18.08 | 120 | 6 | 3 | 1.870828693 |
| *C. arabica* | 18.08 | 120 | 7 | 2 | 1.58113883 |
| *C. arabica* | 18.08 | 120 | 8 | 2 | 1.58113883 |
| *C. arabica* | 18.08 | 120 | 9 | 2 | 1.58113883 |
| *C. arabica* | 18.08 | 120 | 10 | 2 | 1.58113883 |
| *C. arabica* | 18.08 | 120 | 11 | 2 | 1.58113883 |
| *C. arabica* | 18.08 | 120 | 12 | 1 | 1.224744871 |
| *C. arabica* | 18.08 | 120 | 13 | 2 | 1.58113883 |
| *C. arabica* | 18.08 | 120 | 14 | 2 | 1.58113883 |
| *C. arabica* | 18.08 | 120 | 15 | 3 | 1.870828693 |
| *C. arabica* | 18.08 | 150 | 1 | 3 | 1.870828693 |
| *C. arabica* | 18.08 | 150 | 2 | 2 | 1.58113883 |
| *C. arabica* | 18.08 | 150 | 3 | 3 | 1.870828693 |
| *C. arabica* | 18.08 | 150 | 4 | 3 | 1.870828693 |
| *C. arabica* | 18.08 | 150 | 5 | 3 | 1.870828693 |
| *C. arabica* | 18.08 | 150 | 6 | 4 | 2.121320344 |
| *C. arabica* | 18.08 | 150 | 7 | 3 | 1.870828693 |
| *C. arabica* | 18.08 | 150 | 8 | 5 | 2.34520788 |
| *C. arabica* | 18.08 | 150 | 9 | 5 | 2.34520788 |
| *C. arabica* | 18.08 | 150 | 10 | 3 | 1.870828693 |
| *C. arabica* | 18.08 | 150 | 11 | 3 | 1.870828693 |
| *C. arabica* | 18.08 | 150 | 12 | 4 | 2.121320344 |
| *C. arabica* | 18.08 | 150 | 13 | 4 | 2.121320344 |

| | | | | | |
|---|---|---|---|---|---|
| *C. arabica* | 18.08 | 150 | 14 | 4 | 2.121320344 |
| *C. arabica* | 18.08 | 150 | 15 | 3 | 1.870828693 |
| *C. arabica* | 18.08 | 180 | 1 | 4 | 2.121320344 |
| *C. arabica* | 18.08 | 180 | 2 | 5 | 2.34520788 |
| *C. arabica* | 18.08 | 180 | 3 | 4 | 2.121320344 |
| *C. arabica* | 18.08 | 180 | 4 | 4 | 2.121320344 |
| *C. arabica* | 18.08 | 180 | 5 | 5 | 2.34520788 |
| *C. arabica* | 18.08 | 180 | 6 | 5 | 2.34520788 |
| *C. arabica* | 18.08 | 180 | 7 | 5 | 2.34520788 |
| *C. arabica* | 18.08 | 180 | 8 | 6 | 2.549509757 |
| *C. arabica* | 18.08 | 180 | 9 | 5 | 2.34520788 |
| *C. arabica* | 18.08 | 180 | 10 | 6 | 2.549509757 |
| *C. arabica* | 18.08 | 180 | 11 | 6 | 2.549509757 |
| *C. arabica* | 18.08 | 180 | 12 | 5 | 2.34520788 |
| *C. arabica* | 18.08 | 180 | 13 | 6 | 2.549509757 |
| *C. arabica* | 18.08 | 180 | 14 | 5 | 2.34520788 |
| *C. arabica* | 18.08 | 180 | 15 | 5 | 2.34520788 |
| *C. arabica* | 18.08 | 210 | 1 | 6 | 2.549509757 |
| *C. arabica* | 18.08 | 210 | 2 | 6 | 2.549509757 |
| *C. arabica* | 18.08 | 210 | 3 | 6 | 2.549509757 |
| *C. arabica* | 18.08 | 210 | 4 | 6 | 2.549509757 |
| *C. arabica* | 18.08 | 210 | 5 | 6 | 2.549509757 |
| *C. arabica* | 18.08 | 210 | 6 | 6 | 2.549509757 |
| *C. arabica* | 18.08 | 210 | 7 | 6 | 2.549509757 |
| *C. arabica* | 18.08 | 210 | 8 | 6 | 2.549509757 |
| *C. arabica* | 18.08 | 210 | 9 | 6 | 2.549509757 |
| *C. arabica* | 18.08 | 210 | 10 | 6 | 2.549509757 |
| *C. arabica* | 18.08 | 210 | 11 | 6 | 2.549509757 |
| *C. arabica* | 18.08 | 210 | 12 | 6 | 2.549509757 |
| *C. arabica* | 18.08 | 210 | 13 | 7 | 2.738612788 |
| *C. arabica* | 18.08 | 210 | 14 | 7 | 2.738612788 |
| *C. arabica* | 18.08 | 210 | 15 | 6 | 2.549509757 |
| *C. arabica* | 18.08 | 240 | 1 | 7 | 2.738612788 |
| *C. arabica* | 18.08 | 240 | 2 | 7 | 2.738612788 |
| *C. arabica* | 18.08 | 240 | 3 | 7 | 2.738612788 |
| *C. arabica* | 18.08 | 240 | 4 | 8 | 2.915475947 |
| *C. arabica* | 18.08 | 240 | 5 | 7 | 2.738612788 |
| *C. arabica* | 18.08 | 240 | 6 | 8 | 2.915475947 |
| *C. arabica* | 18.08 | 240 | 7 | 8 | 2.915475947 |
| *C. arabica* | 18.08 | 240 | 8 | 7 | 2.738612788 |
| *C. arabica* | 18.08 | 240 | 9 | 7 | 2.738612788 |
| *C. arabica* | 18.08 | 240 | 10 | 7 | 2.738612788 |
| *C. arabica* | 18.08 | 240 | 11 | 7 | 2.738612788 |
| *C. arabica* | 18.08 | 240 | 12 | 8 | 2.915475947 |
| *C. arabica* | 18.08 | 240 | 13 | 7 | 2.738612788 |
| *C. arabica* | 18.08 | 240 | 14 | 8 | 2.915475947 |
| *C. arabica* | 18.08 | 240 | 15 | 8 | 2.915475947 |
| *C. arabica* | 36.24 | 30 | 1 | 0 | 0.707106781 |
| *C. arabica* | 36.24 | 30 | 2 | 0 | 0.707106781 |

| | | | | | |
|---|---|---|---|---|---|
| *C. arabica* | 36.24 | 30 | 3 | 0 | 0.707106781 |
| *C. arabica* | 36.24 | 30 | 4 | 0 | 0.707106781 |
| *C. arabica* | 36.24 | 30 | 5 | 0 | 0.707106781 |
| *C. arabica* | 36.24 | 30 | 6 | 0 | 0.707106781 |
| *C. arabica* | 36.24 | 30 | 7 | 0 | 0.707106781 |
| *C. arabica* | 36.24 | 30 | 8 | 0 | 0.707106781 |
| *C. arabica* | 36.24 | 30 | 9 | 0 | 0.707106781 |
| *C. arabica* | 36.24 | 30 | 10 | 0 | 0.707106781 |
| *C. arabica* | 36.24 | 30 | 11 | 0 | 0.707106781 |
| *C. arabica* | 36.24 | 30 | 12 | 0 | 0.707106781 |
| *C. arabica* | 36.24 | 30 | 13 | 0 | 0.707106781 |
| *C. arabica* | 36.24 | 30 | 14 | 0 | 0.707106781 |
| *C. arabica* | 36.24 | 30 | 15 | 0 | 0.707106781 |
| *C. arabica* | 36.24 | 60 | 1 | 0 | 0.707106781 |
| *C. arabica* | 36.24 | 60 | 2 | 0 | 0.707106781 |
| *C. arabica* | 36.24 | 60 | 3 | 0 | 0.707106781 |
| *C. arabica* | 36.24 | 60 | 4 | 0 | 0.707106781 |
| *C. arabica* | 36.24 | 60 | 5 | 0 | 0.707106781 |
| *C. arabica* | 36.24 | 60 | 6 | 0 | 0.707106781 |
| *C. arabica* | 36.24 | 60 | 7 | 0 | 0.707106781 |
| *C. arabica* | 36.24 | 60 | 8 | 0 | 0.707106781 |
| *C. arabica* | 36.24 | 60 | 9 | 0 | 0.707106781 |
| *C. arabica* | 36.24 | 60 | 10 | 0 | 0.707106781 |
| *C. arabica* | 36.24 | 60 | 11 | 0 | 0.707106781 |
| *C. arabica* | 36.24 | 60 | 12 | 0 | 0.707106781 |
| *C. arabica* | 36.24 | 60 | 13 | 0 | 0.707106781 |
| *C. arabica* | 36.24 | 60 | 14 | 0 | 0.707106781 |
| *C. arabica* | 36.24 | 60 | 15 | 0 | 0.707106781 |
| *C. arabica* | 36.24 | 90 | 1 | 0 | 0.707106781 |
| *C. arabica* | 36.24 | 90 | 2 | 0 | 0.707106781 |
| *C. arabica* | 36.24 | 90 | 3 | 1 | 1.224744871 |
| *C. arabica* | 36.24 | 90 | 4 | 1 | 1.224744871 |
| *C. arabica* | 36.24 | 90 | 5 | 1 | 1.224744871 |
| *C. arabica* | 36.24 | 90 | 6 | 1 | 1.224744871 |
| *C. arabica* | 36.24 | 90 | 7 | 0 | 0.707106781 |
| *C. arabica* | 36.24 | 90 | 8 | 0 | 0.707106781 |
| *C. arabica* | 36.24 | 90 | 9 | 0 | 0.707106781 |
| *C. arabica* | 36.24 | 90 | 10 | 0 | 0.707106781 |
| *C. arabica* | 36.24 | 90 | 11 | 0 | 0.707106781 |
| *C. arabica* | 36.24 | 90 | 12 | 0 | 0.707106781 |
| *C. arabica* | 36.24 | 90 | 13 | 0 | 0.707106781 |
| *C. arabica* | 36.24 | 90 | 14 | 0 | 0.707106781 |
| *C. arabica* | 36.24 | 90 | 15 | 0 | 0.707106781 |
| *C. arabica* | 36.24 | 120 | 1 | 1 | 1.224744871 |
| *C. arabica* | 36.24 | 120 | 2 | 1 | 1.224744871 |
| *C. arabica* | 36.24 | 120 | 3 | 1 | 1.224744871 |
| *C. arabica* | 36.24 | 120 | 4 | 1 | 1.224744871 |
| *C. arabica* | 36.24 | 120 | 5 | 1 | 1.224744871 |
| *C. arabica* | 36.24 | 120 | 6 | 1 | 1.224744871 |

| | | | | | |
|---|---|---|---|---|---|
| *C. arabica* | 36.24 | 120 | 7 | 1 | 1.224744871 |
| *C. arabica* | 36.24 | 120 | 8 | 0 | 0.707106781 |
| *C. arabica* | 36.24 | 120 | 9 | 0 | 0.707106781 |
| *C. arabica* | 36.24 | 120 | 10 | 0 | 0.707106781 |
| *C. arabica* | 36.24 | 120 | 11 | 0 | 0.707106781 |
| *C. arabica* | 36.24 | 120 | 12 | 0 | 0.707106781 |
| *C. arabica* | 36.24 | 120 | 13 | 0 | 0.707106781 |
| *C. arabica* | 36.24 | 120 | 14 | 0 | 0.707106781 |
| *C. arabica* | 36.24 | 120 | 15 | 0 | 0.707106781 |
| *C. arabica* | 36.24 | 150 | 1 | 1 | 1.224744871 |
| *C. arabica* | 36.24 | 150 | 2 | 1 | 1.224744871 |
| *C. arabica* | 36.24 | 150 | 3 | 1 | 1.224744871 |
| *C. arabica* | 36.24 | 150 | 4 | 2 | 1.58113883 |
| *C. arabica* | 36.24 | 150 | 5 | 2 | 1.58113883 |
| *C. arabica* | 36.24 | 150 | 6 | 2 | 1.58113883 |
| *C. arabica* | 36.24 | 150 | 7 | 2 | 1.58113883 |
| *C. arabica* | 36.24 | 150 | 8 | 2 | 1.58113883 |
| *C. arabica* | 36.24 | 150 | 9 | 1 | 1.224744871 |
| *C. arabica* | 36.24 | 150 | 10 | 1 | 1.224744871 |
| *C. arabica* | 36.24 | 150 | 11 | 1 | 1.224744871 |
| *C. arabica* | 36.24 | 150 | 12 | 1 | 1.224744871 |
| *C. arabica* | 36.24 | 150 | 13 | 2 | 1.58113883 |
| *C. arabica* | 36.24 | 150 | 14 | 1 | 1.224744871 |
| *C. arabica* | 36.24 | 150 | 15 | 1 | 1.224744871 |
| *C. arabica* | 36.24 | 180 | 1 | 1 | 1.224744871 |
| *C. arabica* | 36.24 | 180 | 2 | 1 | 1.224744871 |
| *C. arabica* | 36.24 | 180 | 3 | 1 | 1.224744871 |
| *C. arabica* | 36.24 | 180 | 4 | 2 | 1.58113883 |
| *C. arabica* | 36.24 | 180 | 5 | 2 | 1.58113883 |
| *C. arabica* | 36.24 | 180 | 6 | 2 | 1.58113883 |
| *C. arabica* | 36.24 | 180 | 7 | 2 | 1.58113883 |
| *C. arabica* | 36.24 | 180 | 8 | 2 | 1.58113883 |
| *C. arabica* | 36.24 | 180 | 9 | 2 | 1.58113883 |
| *C. arabica* | 36.24 | 180 | 10 | 2 | 1.58113883 |
| *C. arabica* | 36.24 | 180 | 11 | 2 | 1.58113883 |
| *C. arabica* | 36.24 | 180 | 12 | 1 | 1.224744871 |
| *C. arabica* | 36.24 | 180 | 13 | 2 | 1.58113883 |
| *C. arabica* | 36.24 | 180 | 14 | 2 | 1.58113883 |
| *C. arabica* | 36.24 | 180 | 15 | 1 | 1.224744871 |
| *C. arabica* | 36.24 | 210 | 1 | 1 | 1.224744871 |
| *C. arabica* | 36.24 | 210 | 2 | 1 | 1.224744871 |
| *C. arabica* | 36.24 | 210 | 3 | 2 | 1.58113883 |
| *C. arabica* | 36.24 | 210 | 4 | 2 | 1.58113883 |
| *C. arabica* | 36.24 | 210 | 5 | 2 | 1.58113883 |
| *C. arabica* | 36.24 | 210 | 6 | 2 | 1.58113883 |
| *C. arabica* | 36.24 | 210 | 7 | 3 | 1.870828693 |
| *C. arabica* | 36.24 | 210 | 8 | 2 | 1.58113883 |
| *C. arabica* | 36.24 | 210 | 9 | 2 | 1.58113883 |
| *C. arabica* | 36.24 | 210 | 10 | 2 | 1.58113883 |

| | | | | | |
|---|---|---|---|---|---|
| *C. arabica* | 36.24 | 210 | 11 | 2 | 1.58113883 |
| *C. arabica* | 36.24 | 210 | 12 | 2 | 1.58113883 |
| *C. arabica* | 36.24 | 210 | 13 | 2 | 1.58113883 |
| *C. arabica* | 36.24 | 210 | 14 | 2 | 1.58113883 |
| *C. arabica* | 36.24 | 210 | 15 | 2 | 1.58113883 |
| *C. arabica* | 36.24 | 240 | 1 | 1 | 1.224744871 |
| *C. arabica* | 36.24 | 240 | 2 | 1 | 1.224744871 |
| *C. arabica* | 36.24 | 240 | 3 | 2 | 1.58113883 |
| *C. arabica* | 36.24 | 240 | 4 | 2 | 1.58113883 |
| *C. arabica* | 36.24 | 240 | 5 | 2 | 1.58113883 |
| *C. arabica* | 36.24 | 240 | 6 | 2 | 1.58113883 |
| *C. arabica* | 36.24 | 240 | 7 | 3 | 1.870828693 |
| *C. arabica* | 36.24 | 240 | 8 | 2 | 1.58113883 |
| *C. arabica* | 36.24 | 240 | 9 | 2 | 1.58113883 |
| *C. arabica* | 36.24 | 240 | 10 | 2 | 1.58113883 |
| *C. arabica* | 36.24 | 240 | 11 | 2 | 1.58113883 |
| *C. arabica* | 36.24 | 240 | 12 | 2 | 1.58113883 |
| *C. arabica* | 36.24 | 240 | 13 | 2 | 1.58113883 |
| *C. arabica* | 36.24 | 240 | 14 | 2 | 1.58113883 |
| *C. arabica* | 36.24 | 240 | 15 | 2 | 1.58113883 |
| *C. arabica* | 54.36 | 30 | 1 | 0 | 0.707106781 |
| *C. arabica* | 54.36 | 30 | 2 | 0 | 0.707106781 |
| *C. arabica* | 54.36 | 30 | 3 | 0 | 0.707106781 |
| *C. arabica* | 54.36 | 30 | 4 | 0 | 0.707106781 |
| *C. arabica* | 54.36 | 30 | 5 | 0 | 0.707106781 |
| *C. arabica* | 54.36 | 30 | 6 | 0 | 0.707106781 |
| *C. arabica* | 54.36 | 30 | 7 | 0 | 0.707106781 |
| *C. arabica* | 54.36 | 30 | 8 | 0 | 0.707106781 |
| *C. arabica* | 54.36 | 30 | 9 | 0 | 0.707106781 |
| *C. arabica* | 54.36 | 30 | 10 | 0 | 0.707106781 |
| *C. arabica* | 54.36 | 30 | 11 | 0 | 0.707106781 |
| *C. arabica* | 54.36 | 30 | 12 | 0 | 0.707106781 |
| *C. arabica* | 54.36 | 30 | 13 | 0 | 0.707106781 |
| *C. arabica* | 54.36 | 30 | 14 | 0 | 0.707106781 |
| *C. arabica* | 54.36 | 30 | 15 | 0 | 0.707106781 |
| *C. arabica* | 54.36 | 60 | 1 | 1 | 1.224744871 |
| *C. arabica* | 54.36 | 60 | 2 | 1 | 1.224744871 |
| *C. arabica* | 54.36 | 60 | 3 | 1 | 1.224744871 |
| *C. arabica* | 54.36 | 60 | 4 | 1 | 1.224744871 |
| *C. arabica* | 54.36 | 60 | 5 | 1 | 1.224744871 |
| *C. arabica* | 54.36 | 60 | 6 | 0 | 0.707106781 |
| *C. arabica* | 54.36 | 60 | 7 | 1 | 1.224744871 |
| *C. arabica* | 54.36 | 60 | 8 | 0 | 0.707106781 |
| *C. arabica* | 54.36 | 60 | 9 | 1 | 1.224744871 |
| *C. arabica* | 54.36 | 60 | 10 | 0 | 0.707106781 |
| *C. arabica* | 54.36 | 60 | 11 | 0 | 0.707106781 |
| *C. arabica* | 54.36 | 60 | 12 | 0 | 0.707106781 |
| *C. arabica* | 54.36 | 60 | 13 | 0 | 0.707106781 |
| *C. arabica* | 54.36 | 60 | 14 | 0 | 0.707106781 |

| | | | | | |
|---|---|---|---|---|---|
| *C. arabica* | 54.36 | 60 | 15 | 0 | 0.707106781 |
| *C. arabica* | 54.36 | 90 | 1 | 2 | 1.58113883 |
| *C. arabica* | 54.36 | 90 | 2 | 3 | 1.870828693 |
| *C. arabica* | 54.36 | 90 | 3 | 3 | 1.870828693 |
| *C. arabica* | 54.36 | 90 | 4 | 2 | 1.58113883 |
| *C. arabica* | 54.36 | 90 | 5 | 3 | 1.870828693 |
| *C. arabica* | 54.36 | 90 | 6 | 3 | 1.870828693 |
| *C. arabica* | 54.36 | 90 | 7 | 3 | 1.870828693 |
| *C. arabica* | 54.36 | 90 | 8 | 2 | 1.58113883 |
| *C. arabica* | 54.36 | 90 | 9 | 2 | 1.58113883 |
| *C. arabica* | 54.36 | 90 | 10 | 2 | 1.58113883 |
| *C. arabica* | 54.36 | 90 | 11 | 2 | 1.58113883 |
| *C. arabica* | 54.36 | 90 | 12 | 2 | 1.58113883 |
| *C. arabica* | 54.36 | 90 | 13 | 2 | 1.58113883 |
| *C. arabica* | 54.36 | 90 | 14 | 2 | 1.58113883 |
| *C. arabica* | 54.36 | 90 | 15 | 2 | 1.58113883 |
| *C. arabica* | 54.36 | 120 | 1 | 10 | 3.240370349 |
| *C. arabica* | 54.36 | 120 | 2 | 12 | 3.535533906 |
| *C. arabica* | 54.36 | 120 | 3 | 12 | 3.535533906 |
| *C. arabica* | 54.36 | 120 | 4 | 11 | 3.391164992 |
| *C. arabica* | 54.36 | 120 | 5 | 11 | 3.391164992 |
| *C. arabica* | 54.36 | 120 | 6 | 9 | 3.082207001 |
| *C. arabica* | 54.36 | 120 | 7 | 14 | 3.807886553 |
| *C. arabica* | 54.36 | 120 | 8 | 10 | 3.240370349 |
| *C. arabica* | 54.36 | 120 | 9 | 10 | 3.240370349 |
| *C. arabica* | 54.36 | 120 | 10 | 9 | 3.082207001 |
| *C. arabica* | 54.36 | 120 | 11 | 10 | 3.240370349 |
| *C. arabica* | 54.36 | 120 | 12 | 9 | 3.082207001 |
| *C. arabica* | 54.36 | 120 | 13 | 10 | 3.240370349 |
| *C. arabica* | 54.36 | 120 | 14 | 10 | 3.240370349 |
| *C. arabica* | 54.36 | 120 | 15 | 13 | 3.674234614 |
| *C. arabica* | 54.36 | 150 | 1 | 12 | 3.535533906 |
| *C. arabica* | 54.36 | 150 | 2 | 17 | 4.183300133 |
| *C. arabica* | 54.36 | 150 | 3 | 14 | 3.807886553 |
| *C. arabica* | 54.36 | 150 | 4 | 20 | 4.527692569 |
| *C. arabica* | 54.36 | 150 | 5 | 22 | 4.74341649 |
| *C. arabica* | 54.36 | 150 | 6 | 14 | 3.807886553 |
| *C. arabica* | 54.36 | 150 | 7 | 15 | 3.937003937 |
| *C. arabica* | 54.36 | 150 | 8 | 21 | 4.636809248 |
| *C. arabica* | 54.36 | 150 | 9 | 15 | 3.937003937 |
| *C. arabica* | 54.36 | 150 | 10 | 15 | 3.937003937 |
| *C. arabica* | 54.36 | 150 | 11 | 16 | 4.062019202 |
| *C. arabica* | 54.36 | 150 | 12 | 20 | 4.527692569 |
| *C. arabica* | 54.36 | 150 | 13 | 12 | 3.535533906 |
| *C. arabica* | 54.36 | 150 | 14 | 17 | 4.183300133 |
| *C. arabica* | 54.36 | 150 | 15 | 21 | 4.636809248 |
| *C. arabica* | 54.36 | 180 | 1 | 26 | 5.14781507 |
| *C. arabica* | 54.36 | 180 | 2 | 27 | 5.244044241 |
| *C. arabica* | 54.36 | 180 | 3 | 19 | 4.415880433 |

| | | | | | |
|---|---|---|---|---|---|
| *C. arabica* | 54.36 | 180 | 4 | 28 | 5.338539126 |
| *C. arabica* | 54.36 | 180 | 5 | 27 | 5.244044241 |
| *C. arabica* | 54.36 | 180 | 6 | 24 | 4.949747468 |
| *C. arabica* | 54.36 | 180 | 7 | 21 | 4.636809248 |
| *C. arabica* | 54.36 | 180 | 8 | 21 | 4.636809248 |
| *C. arabica* | 54.36 | 180 | 9 | 25 | 5.049752469 |
| *C. arabica* | 54.36 | 180 | 10 | 24 | 4.949747468 |
| *C. arabica* | 54.36 | 180 | 11 | 22 | 4.74341649 |
| *C. arabica* | 54.36 | 180 | 12 | 23 | 4.847679857 |
| *C. arabica* | 54.36 | 180 | 13 | 20 | 4.527692569 |
| *C. arabica* | 54.36 | 180 | 14 | 26 | 5.14781507 |
| *C. arabica* | 54.36 | 180 | 15 | 25 | 5.049752469 |
| *C. arabica* | 54.36 | 210 | 1 | 48 | 6.964194139 |
| *C. arabica* | 54.36 | 210 | 2 | 45 | 6.745368782 |
| *C. arabica* | 54.36 | 210 | 3 | 46 | 6.819090848 |
| *C. arabica* | 54.36 | 210 | 4 | 45 | 6.745368782 |
| *C. arabica* | 54.36 | 210 | 5 | 41 | 6.442049363 |
| *C. arabica* | 54.36 | 210 | 6 | 44 | 6.670832032 |
| *C. arabica* | 54.36 | 210 | 7 | 50 | 7.106335202 |
| *C. arabica* | 54.36 | 210 | 8 | 49 | 7.03562364 |
| *C. arabica* | 54.36 | 210 | 9 | 42 | 6.519202405 |
| *C. arabica* | 54.36 | 210 | 10 | 41 | 6.442049363 |
| *C. arabica* | 54.36 | 210 | 11 | 47 | 6.892024376 |
| *C. arabica* | 54.36 | 210 | 12 | 48 | 6.964194139 |
| *C. arabica* | 54.36 | 210 | 13 | 48 | 6.964194139 |
| *C. arabica* | 54.36 | 210 | 14 | 46 | 6.819090848 |
| *C. arabica* | 54.36 | 210 | 15 | 44 | 6.670832032 |
| *C. arabica* | 54.36 | 240 | 1 | 48 | 6.964194139 |
| *C. arabica* | 54.36 | 240 | 2 | 45 | 6.745368782 |
| *C. arabica* | 54.36 | 240 | 3 | 47 | 6.892024376 |
| *C. arabica* | 54.36 | 240 | 4 | 49 | 7.03562364 |
| *C. arabica* | 54.36 | 240 | 5 | 45 | 6.745368782 |
| *C. arabica* | 54.36 | 240 | 6 | 47 | 6.892024376 |
| *C. arabica* | 54.36 | 240 | 7 | 52 | 7.245688373 |
| *C. arabica* | 54.36 | 240 | 8 | 49 | 7.03562364 |
| *C. arabica* | 54.36 | 240 | 9 | 43 | 6.595452979 |
| *C. arabica* | 54.36 | 240 | 10 | 44 | 6.670832032 |
| *C. arabica* | 54.36 | 240 | 11 | 47 | 6.892024376 |
| *C. arabica* | 54.36 | 240 | 12 | 51 | 7.176350047 |
| *C. arabica* | 54.36 | 240 | 13 | 50 | 7.106335202 |
| *C. arabica* | 54.36 | 240 | 14 | 48 | 6.964194139 |
| *C. arabica* | 54.36 | 240 | 15 | 48 | 6.964194139 |
| *C. canephora* | 9.06 | 30 | 1 | 0 | 0.707106781 |
| *C. canephora* | 9.06 | 30 | 2 | 0 | 0.707106781 |
| *C. canephora* | 9.06 | 30 | 3 | 0 | 0.707106781 |
| *C. canephora* | 9.06 | 30 | 4 | 0 | 0.707106781 |
| *C. canephora* | 9.06 | 30 | 5 | 0 | 0.707106781 |
| *C. canephora* | 9.06 | 30 | 6 | 0 | 0.707106781 |
| *C. canephora* | 9.06 | 30 | 7 | 0 | 0.707106781 |

| | | | | | |
|---|---|---|---|---|---|
| *C. canephora* | 9.06 | 30 | 8 | 0 | 0.707106781 |
| *C. canephora* | 9.06 | 60 | 1 | 0 | 0.707106781 |
| *C. canephora* | 9.06 | 60 | 2 | 0 | 0.707106781 |
| *C. canephora* | 9.06 | 60 | 3 | 0 | 0.707106781 |
| *C. canephora* | 9.06 | 60 | 4 | 0 | 0.707106781 |
| *C. canephora* | 9.06 | 60 | 5 | 0 | 0.707106781 |
| *C. canephora* | 9.06 | 60 | 6 | 0 | 0.707106781 |
| *C. canephora* | 9.06 | 60 | 7 | 0 | 0.707106781 |
| *C. canephora* | 9.06 | 60 | 8 | 0 | 0.707106781 |
| *C. canephora* | 9.06 | 90 | 1 | 0 | 0.707106781 |
| *C. canephora* | 9.06 | 90 | 2 | 0 | 0.707106781 |
| *C. canephora* | 9.06 | 90 | 3 | 0 | 0.707106781 |
| *C. canephora* | 9.06 | 90 | 4 | 0 | 0.707106781 |
| *C. canephora* | 9.06 | 90 | 5 | 0 | 0.707106781 |
| *C. canephora* | 9.06 | 90 | 6 | 0 | 0.707106781 |
| *C. canephora* | 9.06 | 90 | 7 | 0 | 0.707106781 |
| *C. canephora* | 9.06 | 90 | 8 | 0 | 0.707106781 |
| *C. canephora* | 9.06 | 120 | 1 | 0 | 0.707106781 |
| *C. canephora* | 9.06 | 120 | 2 | 0 | 0.707106781 |
| *C. canephora* | 9.06 | 120 | 3 | 0 | 0.707106781 |
| *C. canephora* | 9.06 | 120 | 4 | 0 | 0.707106781 |
| *C. canephora* | 9.06 | 120 | 5 | 0 | 0.707106781 |
| *C. canephora* | 9.06 | 120 | 6 | 0 | 0.707106781 |
| *C. canephora* | 9.06 | 120 | 7 | 0 | 0.707106781 |
| *C. canephora* | 9.06 | 120 | 8 | 0 | 0.707106781 |
| *C. canephora* | 9.06 | 150 | 1 | 0 | 0.707106781 |
| *C. canephora* | 9.06 | 150 | 2 | 0 | 0.707106781 |
| *C. canephora* | 9.06 | 150 | 3 | 0 | 0.707106781 |
| *C. canephora* | 9.06 | 150 | 4 | 0 | 0.707106781 |
| *C. canephora* | 9.06 | 150 | 5 | 0 | 0.707106781 |
| *C. canephora* | 9.06 | 150 | 6 | 0 | 0.707106781 |
| *C. canephora* | 9.06 | 150 | 7 | 0 | 0.707106781 |
| *C. canephora* | 9.06 | 150 | 8 | 0 | 0.707106781 |
| *C. canephora* | 9.06 | 180 | 1 | 1 | 1.224744871 |
| *C. canephora* | 9.06 | 180 | 2 | 1 | 1.224744871 |
| *C. canephora* | 9.06 | 180 | 3 | 1 | 1.224744871 |
| *C. canephora* | 9.06 | 180 | 4 | 1 | 1.224744871 |
| *C. canephora* | 9.06 | 180 | 5 | 0 | 0.707106781 |
| *C. canephora* | 9.06 | 180 | 6 | 0 | 0.707106781 |
| *C. canephora* | 9.06 | 180 | 7 | 0 | 0.707106781 |
| *C. canephora* | 9.06 | 180 | 8 | 0 | 0.707106781 |
| *C. canephora* | 9.06 | 210 | 1 | 2 | 1.58113883 |
| *C. canephora* | 9.06 | 210 | 2 | 2 | 1.58113883 |
| *C. canephora* | 9.06 | 210 | 3 | 2 | 1.58113883 |
| *C. canephora* | 9.06 | 210 | 4 | 2 | 1.58113883 |
| *C. canephora* | 9.06 | 210 | 5 | 1 | 1.224744871 |
| *C. canephora* | 9.06 | 210 | 6 | 1 | 1.224744871 |
| *C. canephora* | 9.06 | 210 | 7 | 1 | 1.224744871 |
| *C. canephora* | 9.06 | 210 | 8 | 2 | 1.58113883 |

| | | | | | |
|---|---|---|---|---|---|
| *C. canephora* | 9.06 | 240 | 1 | 3 | 1.870828693 |
| *C. canephora* | 9.06 | 240 | 2 | 2 | 1.58113883 |
| *C. canephora* | 9.06 | 240 | 3 | 2 | 1.58113883 |
| *C. canephora* | 9.06 | 240 | 4 | 2 | 1.58113883 |
| *C. canephora* | 9.06 | 240 | 5 | 1 | 1.224744871 |
| *C. canephora* | 9.06 | 240 | 6 | 1 | 1.224744871 |
| *C. canephora* | 9.06 | 240 | 7 | 1 | 1.224744871 |
| *C. canephora* | 9.06 | 240 | 8 | 2 | 1.58113883 |
| *C. canephora* | 18.08 | 30 | 1 | 0 | 0.707106781 |
| *C. canephora* | 18.08 | 30 | 2 | 0 | 0.707106781 |
| *C. canephora* | 18.08 | 30 | 3 | 0 | 0.707106781 |
| *C. canephora* | 18.08 | 30 | 4 | 0 | 0.707106781 |
| *C. canephora* | 18.08 | 30 | 5 | 0 | 0.707106781 |
| *C. canephora* | 18.08 | 30 | 6 | 0 | 0.707106781 |
| *C. canephora* | 18.08 | 30 | 7 | 0 | 0.707106781 |
| *C. canephora* | 18.08 | 30 | 8 | 0 | 0.707106781 |
| *C. canephora* | 18.08 | 60 | 1 | 0 | 0.707106781 |
| *C. canephora* | 18.08 | 60 | 2 | 0 | 0.707106781 |
| *C. canephora* | 18.08 | 60 | 3 | 0 | 0.707106781 |
| *C. canephora* | 18.08 | 60 | 4 | 0 | 0.707106781 |
| *C. canephora* | 18.08 | 60 | 5 | 0 | 0.707106781 |
| *C. canephora* | 18.08 | 60 | 6 | 0 | 0.707106781 |
| *C. canephora* | 18.08 | 60 | 7 | 0 | 0.707106781 |
| *C. canephora* | 18.08 | 60 | 8 | 0 | 0.707106781 |
| *C. canephora* | 18.08 | 90 | 1 | 0 | 0.707106781 |
| *C. canephora* | 18.08 | 90 | 2 | 0 | 0.707106781 |
| *C. canephora* | 18.08 | 90 | 3 | 0 | 0.707106781 |
| *C. canephora* | 18.08 | 90 | 4 | 0 | 0.707106781 |
| *C. canephora* | 18.08 | 90 | 5 | 0 | 0.707106781 |
| *C. canephora* | 18.08 | 90 | 6 | 0 | 0.707106781 |
| *C. canephora* | 18.08 | 90 | 7 | 0 | 0.707106781 |
| *C. canephora* | 18.08 | 90 | 8 | 0 | 0.707106781 |
| *C. canephora* | 18.08 | 120 | 1 | 0 | 0.707106781 |
| *C. canephora* | 18.08 | 120 | 2 | 0 | 0.707106781 |
| *C. canephora* | 18.08 | 120 | 3 | 0 | 0.707106781 |
| *C. canephora* | 18.08 | 120 | 4 | 0 | 0.707106781 |
| *C. canephora* | 18.08 | 120 | 5 | 0 | 0.707106781 |
| *C. canephora* | 18.08 | 120 | 6 | 0 | 0.707106781 |
| *C. canephora* | 18.08 | 120 | 7 | 0 | 0.707106781 |
| *C. canephora* | 18.08 | 120 | 8 | 0 | 0.707106781 |
| *C. canephora* | 18.08 | 150 | 1 | 0 | 0.707106781 |
| *C. canephora* | 18.08 | 150 | 2 | 0 | 0.707106781 |
| *C. canephora* | 18.08 | 150 | 3 | 0 | 0.707106781 |
| *C. canephora* | 18.08 | 150 | 4 | 0 | 0.707106781 |
| *C. canephora* | 18.08 | 150 | 5 | 0 | 0.707106781 |
| *C. canephora* | 18.08 | 150 | 6 | 0 | 0.707106781 |
| *C. canephora* | 18.08 | 150 | 7 | 0 | 0.707106781 |
| *C. canephora* | 18.08 | 150 | 8 | 0 | 0.707106781 |
| *C. canephora* | 18.08 | 180 | 1 | 3 | 1.870828693 |

| | | | | | |
|---|---|---|---|---|---|
| *C. canephora* | 18.08 | 180 | 2 | 3 | 1.870828693 |
| *C. canephora* | 18.08 | 180 | 3 | 3 | 1.870828693 |
| *C. canephora* | 18.08 | 180 | 4 | 3 | 1.870828693 |
| *C. canephora* | 18.08 | 180 | 5 | 4 | 2.121320344 |
| *C. canephora* | 18.08 | 180 | 6 | 3 | 1.870828693 |
| *C. canephora* | 18.08 | 180 | 7 | 3 | 1.870828693 |
| *C. canephora* | 18.08 | 180 | 8 | 2 | 1.58113883 |
| *C. canephora* | 18.08 | 210 | 1 | 6 | 2.549509757 |
| *C. canephora* | 18.08 | 210 | 2 | 6 | 2.549509757 |
| *C. canephora* | 18.08 | 210 | 3 | 7 | 2.738612788 |
| *C. canephora* | 18.08 | 210 | 4 | 7 | 2.738612788 |
| *C. canephora* | 18.08 | 210 | 5 | 7 | 2.738612788 |
| *C. canephora* | 18.08 | 210 | 6 | 7 | 2.738612788 |
| *C. canephora* | 18.08 | 210 | 7 | 6 | 2.549509757 |
| *C. canephora* | 18.08 | 210 | 8 | 6 | 2.549509757 |
| *C. canephora* | 18.08 | 240 | 1 | 11 | 3.391164992 |
| *C. canephora* | 18.08 | 240 | 2 | 10 | 3.240370349 |
| *C. canephora* | 18.08 | 240 | 3 | 11 | 3.391164992 |
| *C. canephora* | 18.08 | 240 | 4 | 10 | 3.240370349 |
| *C. canephora* | 18.08 | 240 | 5 | 13 | 3.674234614 |
| *C. canephora* | 18.08 | 240 | 6 | 10 | 3.240370349 |
| *C. canephora* | 18.08 | 240 | 7 | 10 | 3.240370349 |
| *C. canephora* | 18.08 | 240 | 8 | 11 | 3.391164992 |
| *C. canephora* | 36.24 | 30 | 1 | 0 | 0.707106781 |
| *C. canephora* | 36.24 | 30 | 2 | 0 | 0.707106781 |
| *C. canephora* | 36.24 | 30 | 3 | 0 | 0.707106781 |
| *C. canephora* | 36.24 | 30 | 4 | 0 | 0.707106781 |
| *C. canephora* | 36.24 | 30 | 5 | 0 | 0.707106781 |
| *C. canephora* | 36.24 | 30 | 6 | 0 | 0.707106781 |
| *C. canephora* | 36.24 | 30 | 7 | 0 | 0.707106781 |
| *C. canephora* | 36.24 | 30 | 8 | 0 | 0.707106781 |
| *C. canephora* | 36.24 | 60 | 1 | 0 | 0.707106781 |
| *C. canephora* | 36.24 | 60 | 2 | 0 | 0.707106781 |
| *C. canephora* | 36.24 | 60 | 3 | 0 | 0.707106781 |
| *C. canephora* | 36.24 | 60 | 4 | 0 | 0.707106781 |
| *C. canephora* | 36.24 | 60 | 5 | 0 | 0.707106781 |
| *C. canephora* | 36.24 | 60 | 6 | 0 | 0.707106781 |
| *C. canephora* | 36.24 | 60 | 7 | 0 | 0.707106781 |
| *C. canephora* | 36.24 | 60 | 8 | 0 | 0.707106781 |
| *C. canephora* | 36.24 | 90 | 1 | 0 | 0.707106781 |
| *C. canephora* | 36.24 | 90 | 2 | 0 | 0.707106781 |
| *C. canephora* | 36.24 | 90 | 3 | 0 | 0.707106781 |
| *C. canephora* | 36.24 | 90 | 4 | 0 | 0.707106781 |
| *C. canephora* | 36.24 | 90 | 5 | 0 | 0.707106781 |
| *C. canephora* | 36.24 | 90 | 6 | 0 | 0.707106781 |
| *C. canephora* | 36.24 | 90 | 7 | 0 | 0.707106781 |
| *C. canephora* | 36.24 | 90 | 8 | 0 | 0.707106781 |
| *C. canephora* | 36.24 | 120 | 1 | 0 | 0.707106781 |
| *C. canephora* | 36.24 | 120 | 2 | 0 | 0.707106781 |

| | | | | | |
|---|---|---|---|---|---|
| *C. canephora* | 36.24 | 120 | 3 | 0 | 0.707106781 |
| *C. canephora* | 36.24 | 120 | 4 | 0 | 0.707106781 |
| *C. canephora* | 36.24 | 120 | 5 | 0 | 0.707106781 |
| *C. canephora* | 36.24 | 120 | 6 | 0 | 0.707106781 |
| *C. canephora* | 36.24 | 120 | 7 | 0 | 0.707106781 |
| *C. canephora* | 36.24 | 120 | 8 | 0 | 0.707106781 |
| *C. canephora* | 36.24 | 150 | 1 | 0 | 0.707106781 |
| *C. canephora* | 36.24 | 150 | 2 | 0 | 0.707106781 |
| *C. canephora* | 36.24 | 150 | 3 | 0 | 0.707106781 |
| *C. canephora* | 36.24 | 150 | 4 | 0 | 0.707106781 |
| *C. canephora* | 36.24 | 150 | 5 | 0 | 0.707106781 |
| *C. canephora* | 36.24 | 150 | 6 | 0 | 0.707106781 |
| *C. canephora* | 36.24 | 150 | 7 | 0 | 0.707106781 |
| *C. canephora* | 36.24 | 150 | 8 | 0 | 0.707106781 |
| *C. canephora* | 36.24 | 180 | 1 | 2 | 1.58113883 |
| *C. canephora* | 36.24 | 180 | 2 | 2 | 1.58113883 |
| *C. canephora* | 36.24 | 180 | 3 | 2 | 1.58113883 |
| *C. canephora* | 36.24 | 180 | 4 | 1 | 1.224744871 |
| *C. canephora* | 36.24 | 180 | 5 | 1 | 1.224744871 |
| *C. canephora* | 36.24 | 180 | 6 | 0 | 0.707106781 |
| *C. canephora* | 36.24 | 180 | 7 | 0 | 0.707106781 |
| *C. canephora* | 36.24 | 180 | 8 | 1 | 1.224744871 |
| *C. canephora* | 36.24 | 210 | 1 | 4 | 2.121320344 |
| *C. canephora* | 36.24 | 210 | 2 | 4 | 2.121320344 |
| *C. canephora* | 36.24 | 210 | 3 | 4 | 2.121320344 |
| *C. canephora* | 36.24 | 210 | 4 | 3 | 1.870828693 |
| *C. canephora* | 36.24 | 210 | 5 | 3 | 1.870828693 |
| *C. canephora* | 36.24 | 210 | 6 | 4 | 2.121320344 |
| *C. canephora* | 36.24 | 210 | 7 | 4 | 2.121320344 |
| *C. canephora* | 36.24 | 210 | 8 | 4 | 2.121320344 |
| *C. canephora* | 36.24 | 240 | 1 | 6 | 2.549509757 |
| *C. canephora* | 36.24 | 240 | 2 | 4 | 2.121320344 |
| *C. canephora* | 36.24 | 240 | 3 | 4 | 2.121320344 |
| *C. canephora* | 36.24 | 240 | 4 | 4 | 2.121320344 |
| *C. canephora* | 36.24 | 240 | 5 | 5 | 2.34520788 |
| *C. canephora* | 36.24 | 240 | 6 | 5 | 2.34520788 |
| *C. canephora* | 36.24 | 240 | 7 | 4 | 2.121320344 |
| *C. canephora* | 36.24 | 240 | 8 | 4 | 2.121320344 |
| *C. canephora* | 54.36 | 30 | 1 | 0 | 0.707106781 |
| *C. canephora* | 54.36 | 30 | 2 | 0 | 0.707106781 |
| *C. canephora* | 54.36 | 30 | 3 | 0 | 0.707106781 |
| *C. canephora* | 54.36 | 30 | 4 | 0 | 0.707106781 |
| *C. canephora* | 54.36 | 30 | 5 | 0 | 0.707106781 |
| *C. canephora* | 54.36 | 30 | 6 | 0 | 0.707106781 |
| *C. canephora* | 54.36 | 30 | 7 | 0 | 0.707106781 |
| *C. canephora* | 54.36 | 30 | 8 | 0 | 0.707106781 |
| *C. canephora* | 54.36 | 30 | 9 | 0 | 0.707106781 |
| *C. canephora* | 54.36 | 30 | 10 | 0 | 0.707106781 |
| *C. canephora* | 54.36 | 30 | 11 | 0 | 0.707106781 |

| | | | | | |
|---|---|---|---|---|---|
| *C. canephora* | 54.36 | 30 | 12 | 0 | 0.707106781 |
| *C. canephora* | 54.36 | 30 | 13 | 0 | 0.707106781 |
| *C. canephora* | 54.36 | 30 | 14 | 0 | 0.707106781 |
| *C. canephora* | 54.36 | 30 | 15 | 0 | 0.707106781 |
| *C. canephora* | 54.36 | 30 | 16 | 0 | 0.707106781 |
| *C. canephora* | 54.36 | 30 | 17 | 0 | 0.707106781 |
| *C. canephora* | 54.36 | 30 | 18 | 0 | 0.707106781 |
| *C. canephora* | 54.36 | 30 | 19 | 0 | 0.707106781 |
| *C. canephora* | 54.36 | 30 | 20 | 0 | 0.707106781 |
| *C. canephora* | 54.36 | 60 | 1 | 0 | 0.707106781 |
| *C. canephora* | 54.36 | 60 | 2 | 0 | 0.707106781 |
| *C. canephora* | 54.36 | 60 | 3 | 0 | 0.707106781 |
| *C. canephora* | 54.36 | 60 | 4 | 0 | 0.707106781 |
| *C. canephora* | 54.36 | 60 | 5 | 0 | 0.707106781 |
| *C. canephora* | 54.36 | 60 | 6 | 0 | 0.707106781 |
| *C. canephora* | 54.36 | 60 | 7 | 0 | 0.707106781 |
| *C. canephora* | 54.36 | 60 | 8 | 0 | 0.707106781 |
| *C. canephora* | 54.36 | 60 | 9 | 0 | 0.707106781 |
| *C. canephora* | 54.36 | 60 | 10 | 0 | 0.707106781 |
| *C. canephora* | 54.36 | 60 | 11 | 0 | 0.707106781 |
| *C. canephora* | 54.36 | 60 | 12 | 0 | 0.707106781 |
| *C. canephora* | 54.36 | 60 | 13 | 0 | 0.707106781 |
| *C. canephora* | 54.36 | 60 | 14 | 0 | 0.707106781 |
| *C. canephora* | 54.36 | 60 | 15 | 0 | 0.707106781 |
| *C. canephora* | 54.36 | 60 | 16 | 0 | 0.707106781 |
| *C. canephora* | 54.36 | 60 | 17 | 0 | 0.707106781 |
| *C. canephora* | 54.36 | 60 | 18 | 0 | 0.707106781 |
| *C. canephora* | 54.36 | 60 | 19 | 0 | 0.707106781 |
| *C. canephora* | 54.36 | 60 | 20 | 0 | 0.707106781 |
| *C. canephora* | 54.36 | 90 | 1 | 0 | 0.707106781 |
| *C. canephora* | 54.36 | 90 | 2 | 0 | 0.707106781 |
| *C. canephora* | 54.36 | 90 | 3 | 1 | 1.224744871 |
| *C. canephora* | 54.36 | 90 | 4 | 1 | 1.224744871 |
| *C. canephora* | 54.36 | 90 | 5 | 1 | 1.224744871 |
| *C. canephora* | 54.36 | 90 | 6 | 0 | 0.707106781 |
| *C. canephora* | 54.36 | 90 | 7 | 0 | 0.707106781 |
| *C. canephora* | 54.36 | 90 | 8 | 0 | 0.707106781 |
| *C. canephora* | 54.36 | 90 | 9 | 0 | 0.707106781 |
| *C. canephora* | 54.36 | 90 | 10 | 0 | 0.707106781 |
| *C. canephora* | 54.36 | 90 | 11 | 0 | 0.707106781 |
| *C. canephora* | 54.36 | 90 | 12 | 0 | 0.707106781 |
| *C. canephora* | 54.36 | 90 | 13 | 0 | 0.707106781 |
| *C. canephora* | 54.36 | 90 | 14 | 0 | 0.707106781 |
| *C. canephora* | 54.36 | 90 | 15 | 0 | 0.707106781 |
| *C. canephora* | 54.36 | 90 | 16 | 0 | 0.707106781 |
| *C. canephora* | 54.36 | 90 | 17 | 0 | 0.707106781 |
| *C. canephora* | 54.36 | 90 | 18 | 0 | 0.707106781 |
| *C. canephora* | 54.36 | 90 | 19 | 0 | 0.707106781 |
| *C. canephora* | 54.36 | 90 | 20 | 0 | 0.707106781 |

| | | | | | |
|---|---|---|---|---|---|
| *C. canephora* | 54.36 | 120 | 1 | 2 | 1.58113883 |
| *C. canephora* | 54.36 | 120 | 2 | 2 | 1.58113883 |
| *C. canephora* | 54.36 | 120 | 3 | 2 | 1.58113883 |
| *C. canephora* | 54.36 | 120 | 4 | 3 | 1.870828693 |
| *C. canephora* | 54.36 | 120 | 5 | 2 | 1.58113883 |
| *C. canephora* | 54.36 | 120 | 6 | 1 | 1.224744871 |
| *C. canephora* | 54.36 | 120 | 7 | 1 | 1.224744871 |
| *C. canephora* | 54.36 | 120 | 8 | 1 | 1.224744871 |
| *C. canephora* | 54.36 | 120 | 9 | 1 | 1.224744871 |
| *C. canephora* | 54.36 | 120 | 10 | 1 | 1.224744871 |
| *C. canephora* | 54.36 | 120 | 11 | 2 | 1.58113883 |
| *C. canephora* | 54.36 | 120 | 12 | 0 | 0.707106781 |
| *C. canephora* | 54.36 | 120 | 13 | 3 | 1.870828693 |
| *C. canephora* | 54.36 | 120 | 14 | 1 | 1.224744871 |
| *C. canephora* | 54.36 | 120 | 15 | 1 | 1.224744871 |
| *C. canephora* | 54.36 | 120 | 16 | 3 | 1.870828693 |
| *C. canephora* | 54.36 | 120 | 17 | 3 | 1.870828693 |
| *C. canephora* | 54.36 | 120 | 18 | 0 | 0.707106781 |
| *C. canephora* | 54.36 | 120 | 19 | 0 | 0.707106781 |
| *C. canephora* | 54.36 | 120 | 20 | 0 | 0.707106781 |
| *C. canephora* | 54.36 | 150 | 1 | 5 | 2.34520788 |
| *C. canephora* | 54.36 | 150 | 2 | 6 | 2.549509757 |
| *C. canephora* | 54.36 | 150 | 3 | 5 | 2.34520788 |
| *C. canephora* | 54.36 | 150 | 4 | 6 | 2.549509757 |
| *C. canephora* | 54.36 | 150 | 5 | 5 | 2.34520788 |
| *C. canephora* | 54.36 | 150 | 6 | 5 | 2.34520788 |
| *C. canephora* | 54.36 | 150 | 7 | 5 | 2.34520788 |
| *C. canephora* | 54.36 | 150 | 8 | 5 | 2.34520788 |
| *C. canephora* | 54.36 | 150 | 9 | 5 | 2.34520788 |
| *C. canephora* | 54.36 | 150 | 10 | 5 | 2.34520788 |
| *C. canephora* | 54.36 | 150 | 11 | 5 | 2.34520788 |
| *C. canephora* | 54.36 | 150 | 12 | 5 | 2.34520788 |
| *C. canephora* | 54.36 | 150 | 13 | 5 | 2.34520788 |
| *C. canephora* | 54.36 | 150 | 14 | 7 | 2.738612788 |
| *C. canephora* | 54.36 | 150 | 15 | 6 | 2.549509757 |
| *C. canephora* | 54.36 | 150 | 16 | 6 | 2.549509757 |
| *C. canephora* | 54.36 | 150 | 17 | 6 | 2.549509757 |
| *C. canephora* | 54.36 | 150 | 18 | 6 | 2.549509757 |
| *C. canephora* | 54.36 | 150 | 19 | 5 | 2.34520788 |
| *C. canephora* | 54.36 | 150 | 20 | 6 | 2.549509757 |
| *C. canephora* | 54.36 | 180 | 1 | 10 | 3.240370349 |
| *C. canephora* | 54.36 | 180 | 2 | 12 | 3.535533906 |
| *C. canephora* | 54.36 | 180 | 3 | 11 | 3.391164992 |
| *C. canephora* | 54.36 | 180 | 4 | 15 | 3.937003937 |
| *C. canephora* | 54.36 | 180 | 5 | 13 | 3.674234614 |
| *C. canephora* | 54.36 | 180 | 6 | 12 | 3.535533906 |
| *C. canephora* | 54.36 | 180 | 7 | 13 | 3.674234614 |
| *C. canephora* | 54.36 | 180 | 8 | 11 | 3.391164992 |
| *C. canephora* | 54.36 | 180 | 9 | 10 | 3.240370349 |

| | | | | | |
|---|---|---|---|---|---|
| *C. canephora* | 54.36 | 180 | 10 | 10 | 3.240370349 |
| *C. canephora* | 54.36 | 180 | 11 | 12 | 3.535533906 |
| *C. canephora* | 54.36 | 180 | 12 | 11 | 3.391164992 |
| *C. canephora* | 54.36 | 180 | 13 | 15 | 3.937003937 |
| *C. canephora* | 54.36 | 180 | 14 | 11 | 3.391164992 |
| *C. canephora* | 54.36 | 180 | 15 | 12 | 3.535533906 |
| *C. canephora* | 54.36 | 180 | 16 | 15 | 3.937003937 |
| *C. canephora* | 54.36 | 180 | 17 | 11 | 3.391164992 |
| *C. canephora* | 54.36 | 180 | 18 | 14 | 3.807886553 |
| *C. canephora* | 54.36 | 180 | 19 | 11 | 3.391164992 |
| *C. canephora* | 54.36 | 180 | 20 | 12 | 3.535533906 |
| *C. canephora* | 54.36 | 210 | 1 | 18 | 4.301162634 |
| *C. canephora* | 54.36 | 210 | 2 | 23 | 4.847679857 |
| *C. canephora* | 54.36 | 210 | 3 | 18 | 4.301162634 |
| *C. canephora* | 54.36 | 210 | 4 | 24 | 4.949747468 |
| *C. canephora* | 54.36 | 210 | 5 | 16 | 4.062019202 |
| *C. canephora* | 54.36 | 210 | 6 | 18 | 4.301162634 |
| *C. canephora* | 54.36 | 210 | 7 | 17 | 4.183300133 |
| *C. canephora* | 54.36 | 210 | 8 | 16 | 4.062019202 |
| *C. canephora* | 54.36 | 210 | 9 | 16 | 4.062019202 |
| *C. canephora* | 54.36 | 210 | 10 | 19 | 4.415880433 |
| *C. canephora* | 54.36 | 210 | 11 | 22 | 4.74341649 |
| *C. canephora* | 54.36 | 210 | 12 | 17 | 4.183300133 |
| *C. canephora* | 54.36 | 210 | 13 | 19 | 4.415880433 |
| *C. canephora* | 54.36 | 210 | 14 | 16 | 4.062019202 |
| *C. canephora* | 54.36 | 210 | 15 | 16 | 4.062019202 |
| *C. canephora* | 54.36 | 210 | 16 | 21 | 4.636809248 |
| *C. canephora* | 54.36 | 210 | 17 | 18 | 4.301162634 |
| *C. canephora* | 54.36 | 210 | 18 | 18 | 4.301162634 |
| *C. canephora* | 54.36 | 210 | 19 | 20 | 4.527692569 |
| *C. canephora* | 54.36 | 210 | 20 | 19 | 4.415880433 |
| *C. canephora* | 54.36 | 240 | 1 | 20 | 4.527692569 |
| *C. canephora* | 54.36 | 240 | 2 | 23 | 4.847679857 |
| *C. canephora* | 54.36 | 240 | 3 | 22 | 4.74341649 |
| *C. canephora* | 54.36 | 240 | 4 | 24 | 4.949747468 |
| *C. canephora* | 54.36 | 240 | 5 | 19 | 4.415880433 |
| *C. canephora* | 54.36 | 240 | 6 | 18 | 4.301162634 |
| *C. canephora* | 54.36 | 240 | 7 | 17 | 4.183300133 |
| *C. canephora* | 54.36 | 240 | 8 | 18 | 4.301162634 |
| *C. canephora* | 54.36 | 240 | 9 | 18 | 4.301162634 |
| *C. canephora* | 54.36 | 240 | 10 | 19 | 4.415880433 |
| *C. canephora* | 54.36 | 240 | 11 | 22 | 4.74341649 |
| *C. canephora* | 54.36 | 240 | 12 | 18 | 4.301162634 |
| *C. canephora* | 54.36 | 240 | 13 | 19 | 4.415880433 |
| *C. canephora* | 54.36 | 240 | 14 | 18 | 4.301162634 |
| *C. canephora* | 54.36 | 240 | 15 | 18 | 4.301162634 |
| *C. canephora* | 54.36 | 240 | 16 | 21 | 4.636809248 |
| *C. canephora* | 54.36 | 240 | 17 | 18 | 4.301162634 |
| *C. canephora* | 54.36 | 240 | 18 | 18 | 4.301162634 |

| | | | | | |
|---|---|---|---|---|---|
| *C. canephora* | 54.36 | 240 | 19 | 21 | 4.636809248 |
| *C. canephora* | 54.36 | 240 | 20 | 27 | 5.244044241 |
