# Supplementary material for: Embryonic abnormalities and genotoxicity induced by 2,4-dichlorophenoxyacetic acid during indirect somatic embryogenesis in Coffea
Source: Sci Rep. 2023 Jun 15;13:9689. doi: 10.1038/s41598-023-36879-7 (PMC10272143; doi:10.1038/s41598-023-36879-7)
Supplement: Supplementary file 7 — Supplementary Table 4. [file 41598_2023_36879_MOESM7_ESM.pdf]

SI Table 4. All data generated and analysed about the 5-mC% measured for the plant explant donors, friable callus, embryogenic friable callus, mature cotyledon somatic embryo (MCSE) and abnormal somatic embryo (ASE) of *C. arabica* and *C. canephora*.

Plant explant donor		
Coffea species	Repetition	5-mC%
C. arabica	1	23.00
C. arabica	2	18.40
C. arabica	3	20.60
C. canephora	1	23.00
C. canephora	2	18.40
C. canephora	3	20.60

Friable callus			
Coffea species	Treatment	Repetition	5-mC%
C. arabica	9.06	1	15.70
C. arabica	9.06	2	23.50
C. arabica	9.06	3	11.80
C. arabica	9.06	4	12.10
C. arabica	9.06	5	25.40
C. arabica	18.08	1	25.20
C. arabica	18.08	2	31.60
C. arabica	18.08	3	16.10
C. arabica	18.08	4	16.60
C. arabica	18.08	5	19.30
C. arabica	36.24	1	18.50
C. arabica	36.24	2	13.30
C. arabica	36.24	3	17.00
C. arabica	36.24	4	15.60
C. arabica	36.24	5	13.80
C. arabica	54.36	1	11.40
C. arabica	54.36	2	11.70
C. arabica	54.36	3	12.20
C. arabica	54.36	4	11.30
C. arabica	54.36	5	10.80
C. canephora	9.06	1	23.40
C. canephora	9.06	2	22.80
C. canephora	9.06	3	23.90
C. canephora	9.06	4	18.60
C. canephora	9.06	5	17.40
C. canephora	18.08	1	12.10
C. canephora	18.08	2	11.20
C. canephora	18.08	3	16.00
C. canephora	18.08	4	9.60
C. canephora	18.08	5	15.30
C. canephora	36.24	1	11.80
C. canephora	36.24	2	15.00
C. canephora	36.24	3	18.00
C. canephora	36.24	4	15.60
C. canephora	36.24	5	12.60

C. canephora	54.36	1	19.40
C. canephora	54.36	2	25.60
C. canephora	54.36	3	21.50
C. canephora	54.36	4	28.10
C. canephora	54.36	5	26.40

Friable embryogenic callus			
Coffea species	2,4-D (μM)	Repetition	5-mC%
C. arabica	9.06	1	23.70
C. arabica	9.06	2	25.70
C. arabica	9.06	3	30.60
C. arabica	18.08	1	27.50
C. arabica	18.08	2	30.40
C. arabica	18.08	3	27.10
C. arabica	36.24	1	19.80
C. arabica	36.24	2	37.40
C. arabica	36.24	3	33.20
C. arabica	54.36	1	18.20
C. arabica	54.36	2	19.20
C. arabica	54.36	3	28.50
C. canephora	9.06	1	42.60
C. canephora	9.06	2	48.50
C. canephora	9.06	3	43.60
C. canephora	18.08	1	39.20
C. canephora	18.08	2	40.20
C. canephora	18.08	3	45.90
C. canephora	36.24	1	44.50
C. canephora	36.24	2	45.70
C. canephora	36.24	3	40.60
C. canephora	54.36	1	48.20
C. canephora	54.36	2	44.70
C. canephora	54.36	3	43.90

Somatic embryo			
Coffea species	Somatic embryo (2,4-D μM)	Repetition	5-mC%
C. arabica	MCSE (control)	1	9
C. arabica	MCSE (control)	2	8.4
C. arabica	MCSE (control)	3	9.4
C. arabica	ASE (9.06)	1	9.80
C. arabica	ASE (9.06)	2	11.20
C. arabica	ASE (9.06)	3	12.80
C. arabica	ASE (18.08)	1	12.80
C. arabica	ASE (18.08)	2	17.60
C. arabica	ASE (18.08)	3	15.70
C. arabica	ASE (36.24)	1	16.50
C. arabica	ASE (36.24)	2	22.90
C. arabica	ASE (36.24)	3	15.80
C. arabica	ASE (54.36)	1	27.20
C. arabica	ASE (54.36)	2	22.90

C. arabica	ASE (54.36)	3	23.00
C. canephora	MCSE (control)	1	13
C. canephora	MCSE (control)	2	10.23
C. canephora	MCSE (control)	3	9.85
C. canephora	ASE (9.06)	1	NA
C. canephora	ASE (9.06)	2	NA
C. canephora	ASE (9.06)	3	NA
C. canephora	ASE (18.08)	1	NA
C. canephora	ASE (18.08)	2	NA
C. canephora	ASE (18.08)	3	NA
C. canephora	ASE (36.24)	1	NA
C. canephora	ASE (36.24)	2	NA
C. canephora	ASE (36.24)	3	NA
C. canephora	ASE (54.36)	1	19.30
C. canephora	ASE (54.36)	2	18.00
C. canephora	ASE (54.36)	3	28.60
