# Supplementary material for: Embryonic abnormalities and genotoxicity induced by 2,4-dichlorophenoxyacetic acid during indirect somatic embryogenesis in Coffea
Source: Sci Rep. 2023 Jun 15;13:9689. doi: 10.1038/s41598-023-36879-7 (PMC10272143; doi:10.1038/s41598-023-36879-7)
Supplement: Supplementary file 8 — Supplementary Table 5. [file 41598_2023_36879_MOESM8_ESM.pdf]

SI Table 5. All data generated and analysed about Comet Assay (nuclei with DNA damage) measured for mature cotyledon somatic embryo (MCSE) and abnormal somatic embryo (ASE) of *C. arabica* and *C. canephora*.

Coffea species	Somatic embryo (2,4-D μM)	Repetition	Nuclei with DNA damage	DNA damage (%)
C. arabica	MCSE (control)	1	7	2.333333333
C. arabica	MCSE (control)	2	8	2.666666667
C. arabica	MCSE (control)	3	12	3.833333333
C. arabica	MCSE (control)	4	12	3.833333333
C. arabica	ASE (9.06)	1	50	16.66666667
C. arabica	ASE (9.06)	2	44	14.5
C. arabica	ASE (9.06)	3	55	18.33333333
C. arabica	ASE (9.06)	4	50	16.66666667
C. arabica	ASE (18.08)	1	57	19
C. arabica	ASE (18.08)	2	82	27.33333333
C. arabica	ASE (18.08)	3	54	18
C. arabica	ASE (18.08)	4	95	31.66666667
C. arabica	ASE (36.24)	1	72	23.83333333
C. arabica	ASE (36.24)	2	78	25.83333333
C. arabica	ASE (36.24)	3	81	26.83333333
C. arabica	ASE (36.24)	4	67	22.16666667
C. arabica	ASE (54.36)	1	90	29.83333333
C. arabica	ASE (54.36)	2	72	23.83333333
C. arabica	ASE (54.36)	3	80	26.66666667
C. arabica	ASE (54.36)	4	92	30.66666667
C. canephora	MCSE (control)	1	13	4.166666667
C. canephora	MCSE (control)	2	17	5.5
C. canephora	MCSE (control)	3	13	4.166666667
C. canephora	MCSE (control)	4	20	6.5
C. canephora	ASE (9.06)	1	86	28.5
C. canephora	ASE (9.06)	2	86	28.5
C. canephora	ASE (9.06)	3	96	32
C. canephora	ASE (9.06)	4	108	36
C. canephora	ASE (18.08)	1	105	34.83333333
C. canephora	ASE (18.08)	2	100	33.16666667
C. canephora	ASE (18.08)	3	96	31.83333333
C. canephora	ASE (18.08)	4	92	30.5
C. canephora	ASE (36.24)	1	105	35
C. canephora	ASE (36.24)	2	90	29.83333333
C. canephora	ASE (36.24)	3	101	33.5
C. canephora	ASE (36.24)	4	120	40
C. canephora	ASE (54.36)	1	99	33
C. canephora	ASE (54.36)	2	92	30.5
C. canephora	ASE (54.36)	3	115	38.33333333
C. canephora	ASE (54.36)	4	111	36.83333333
